# Cullin-RING ligases employ geometrically optimized catalytic partners for substrate targeting

**DOI:** 10.1016/j.molcel.2024.01.022

**Published:** 2024-02-20

**Authors:** Jerry Li, Nicholas Purser, Joanna Liwocha, Daniel C. Scott, Holly A. Byers, Barbara Steigenberger, Spencer Hill, Ishita Tripathi-Giesgen, Trent Hinkle, Fynn M. Hansen, J. Rajan Prabu, Senthil K. Radhakrishnan, Donald S. Kirkpatrick, Kurt M. Reichermeier, Brenda A. Schulman, Gary Kleiger

**Affiliations:** 1 Department of Chemistry and Biochemistry, University of Nevada, Las Vegas, Las Vegas, NV 89154, USA; 2 Department of Molecular Machines and Signaling, Max Planck Institute of Biochemistry, Martinsried 82152, Germany; 3 Department of Structural Biology, St. Jude Children’s Research Hospital, Memphis, TN 38105, USA; 4 Department of Pathology, Virginia Commonwealth University, Richmond, VA 23298, USA; 5 Mass Spectrometry Core Facility, Max Planck Institute of Biochemistry, Martinsried 82152, Germany; 6 Genentech, 1 DNA Way, South San Francisco, CA 94080, USA; 7 Department of Proteomics and Signal Transduction, Max Planck Institute of Biochemistry, Martinsried 82152, Germany; 8 Present address: Orion Medicines, Brisbane, CA 94005, USA; 9 Present address: University of Texas Southwestern Medical Center, Department of Internal Medicine, Division of Pulmonary & Critical Care Medicine, Dallas, TX 75390, USA; 10 These authors contributed equally; 11 Lead contact

## Abstract

Cullin-RING ligases (CRLs) ubiquitylate specific substrates selected from other cellular proteins. Substrate discrimination and ubiquitin transferase activity were thought to be strictly separated. Substrates are recognized by substrate receptors, such as Fbox or BCbox proteins. Meanwhile, CRLs employ assorted ubiquitin-carrying enzymes (UCEs, which are a collection of E2 and ARIH-family E3s) specialized for either initial substrate ubiquitylation (priming) or forging poly-ubiquitin chains. We discovered specific human CRL-UCE pairings governing substrate priming. The results reveal pairing of CUL2-based CRLs and UBE2R-family UCEs in cells, essential for efficient PROTAC-induced neo-substrate degradation. Despite UBE2R2’s intrinsic programming to catalyze poly-ubiquitylation, CUL2 employs this UCE for geometrically precise PROTAC-dependent ubiquitylation of a neo-substrate and for rapid priming of substrates recruited to diverse receptors. Cryo-EM structures illuminate how CUL2-based CRLs engage UBE2R2 to activate substrate ubiquitylation. Thus, pairing with a specific UCE overcomes E2 catalytic limitations to drive substrate ubiquitylation and targeted protein degradation.

## In brief

How do E3 ligases target substrates and degrader-recruited neo-substrates for degradation? Although E3s typically harbor substrate binding sites, Li et al. demonstrate that ubiquitin-carrying enzymes promoting catalysis may also participate in substrate selection. Avid E3-E2 interactions help to juxtapose the activated ubiquitin with the substrate.

## Graphical Abstract

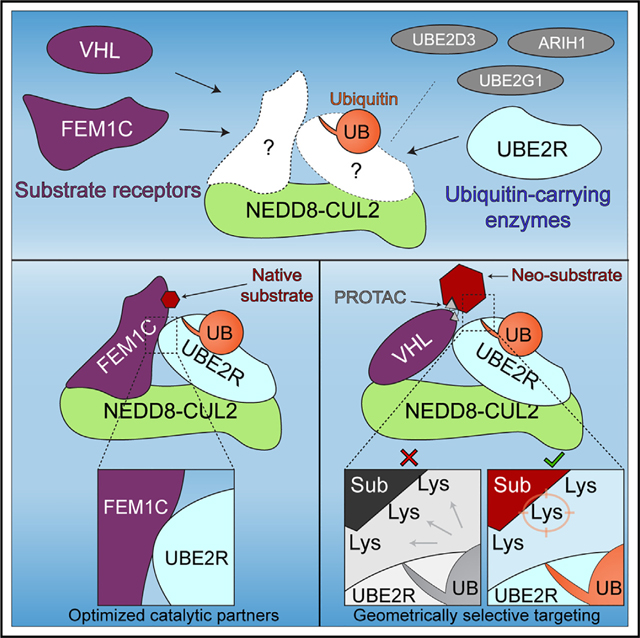

## INTRODUCTION

Cullin-RING ubiquitin ligases (CRLs) comprise one of the most important cellular regulatory systems.^1^ Mutations in specific CRLs cause developmental disorders, hypertension, and numerous cancers.^2^ CRLs are hijacked by pathogens to promote infections.^3^ And CRLs are central to a multi-billion-dollar targeted protein degradation (TPD) industry, whereby small molecules direct E3s to ubiquitylate proteins (termed neo-substrates) with roles in human disease.^4–9^ Thus, defining the molecular mechanisms of substrate targeting by CRLs is of fundamental importance.

CRLs exert broad regulation by forming a very large family (≈300 members in humans) of modular multi-protein complexes with similar architectures. Substrate receptor modules contain a subunit that directly binds to the protein substrate. The scaffolding module contains an elongated cullin-family (or “CUL”) protein that on one end connects to the substrate receptor and, on the other, to a RING domain-containing RBX-family protein. The substrate receptor-cullin-RING complex nomenclature, CRL#^X^, describes the holoenzyme, where “#” refers to the human CUL paralog and “X” to the substrate receptor. A CRL’s E3 ligase activity is controlled by linkage of the ubiquitin-like protein NEDD8 to the cullin subunit.^10,11^ Neddylation and deneddylation are regulated by two large factors, CSN and CAND1.^12–16^ In the absence of substrate, NEDD8 is removed by CSN, and CAND1 both inhibits and often dismantles the inactive CRL.^17,18^

Ever since the CRL architecture was elucidated more than 25 years ago, substrate targeting was thought to be exclusively determined by the identity of a CRL’s substrate receptor, such as Fbox, BCbox, BTB, and DCAF proteins.^19^ For example, distinct Fbox proteins (together with the SKP1 adaptor) regulate the cell cycle, various signaling pathways, and circadian rhythms by recruiting specific substrates to neddylated CUL1-RBX1.^20^ Similarly, distinct BCbox proteins (together with ELONGIN B/C adaptors) are mutated in diseases^21,22^ and regulate hypoxic and redox stress responses^23–25^ by recruiting specific substrates to neddylated CUL2-RBX1.

Catalysis of ubiquitylation has been considered as a strictly separate function from substrate targeting that is executed when the RING domain bound to a neddylated cullin encounters a ubiquitin-carrying enzyme (UCE). We use the term UCE, first suggested by Cecile Pickart and Irwin Rose,^26^ since the collection of such enzymes employed by CRL RING domains to perform ubiquitylation include ARIH-family E3s as well as various E2s. Although early studies in yeast identified a single UCE, Cdc34, as responsible for ubiquitylation by CRL1-family E3s,^27,28^ the relatively inefficient direct modification of CRL1 substrates in reactions by human orthologs (UBE2R1 and UBE2R2) led to discovery of eight UCEs functioning with human CRLs.^29–37^

Roles of the assorted human UCEs have been largely defined by studies of CRL1- and CRL4-family E3s. The widely held dogma is that some UCEs (UBE2D-family E2s and ARIH-family E3s) directly modify proteins in a so-called “priming” reaction that modifies the substrate with the initial ubiquitin.^30–35,38–40^ Others (UBE2R-family and UBE2G1 E2s) are thought to be hard-wired to extend poly-ubiquitin chains linked through ubiquitin’s Lys48^36,41–43^ to promote proteasomal degradation.^44^ When thioester-bonded to ubiquitin (hereafter E2~ubiquitin), such chain-forming E2s intrinsically form the catalytic closed conformation but are restricted from nefarious substrate priming by their inactivity toward lysines other than from ubiquitin. As a whole, the cohort of E2~ubiquitin representatives that can adopt the closed conformation in the absence of E3 build linkage-specific poly-ubiquitin chains on their own,^45–47^ and their active sites are thought to be complemented by ubiquitin performing substrate-assisted catalysis.^43,45^ For such E2s that exclusively perform reactions “extending” a substrate’s poly-ubiquitin chain, the E3 is thought to primarily direct chain-building activity toward ubiquitin-primed substrates. For CUL1 complexes, the multiplicity of UCEs across diverse enzyme families that catalyze either priming or extending are functionally redundant and allow for robust ubiquitin-mediated degradation even in the absence of the entire UBE2R family of E2s.^37^

Although ubiquitin priming of CRL1 substrates has been well defined,^38,39^ including structurally, the extent to which these mechanisms apply to CRL2-family E3s is largely unknown. Yet, CRL2s are of particular interest, from responsibility of the foundational member—CRL2^VHL^—for von Hippel-Lindau disease,^23,48,49^ to emerging roles of several CRL2 E3s in recognizing degrons at protein N or C termini,^50,51^ as well as their employment by proteolysis targeting chimeras (PROTACs) that induce proximity between E3s and neo-substrates, causing the latter’s degradation.^52–55^ Indeed, tens of human BCbox proteins are thought to be substrate receptors functioning with CUL2-RBX1.^56,57^

Here, using a combination of biochemical, cell biological, and structural approaches, we unexpectedly discovered that the UCEs catalyzing the crucial first step of ubiquitin-mediated regulation—priming of substrate—differ between CUL1- and CUL2-based CRLs. Pursuing regulation by human CRL2s and UBE2R-family E2s revealed that substrate—and degrader-recruited neo-substrate—targeting is not solely determined by the substrate receptor but also depends on distinct UCE pairing. The results demonstrate that specific UCEs function with subgroups of the CRLs and reveal how CUL2-based CRLs can reprogram the robust poly-ubiquitin chain-forming activity of the E2 UBE2R2 toward geometrically optimized substrate priming.

## RESULTS

### UBE2R2 displays specificity with CRL2s in comparison with other CRL-dependent UCEs

Prior studies of neddylated CRL1s and CRL4s led to two major conclusions. First, CRL1s preferentially prime substrates with ARIH1 and/or a UBE2D-family E2.^30,32^ Second, although UBE2R-family E2s are inefficient at priming CRL substrates,^33,37^ they forge Lys48-linked poly-ubiquitin chains on their own^36,58,59^ and extremely efficiently onto primed CRL1 substrates.^60^ For example, UBE2D-family members promote rapid substrate priming in combination with CRL1^FBXW1^ in comparison with ARIH1,^38^ whereas ARIH1 is far more efficient at priming with CRL1^FBXW7^ than UBE2D E2s.^39^

**Figure 1.**
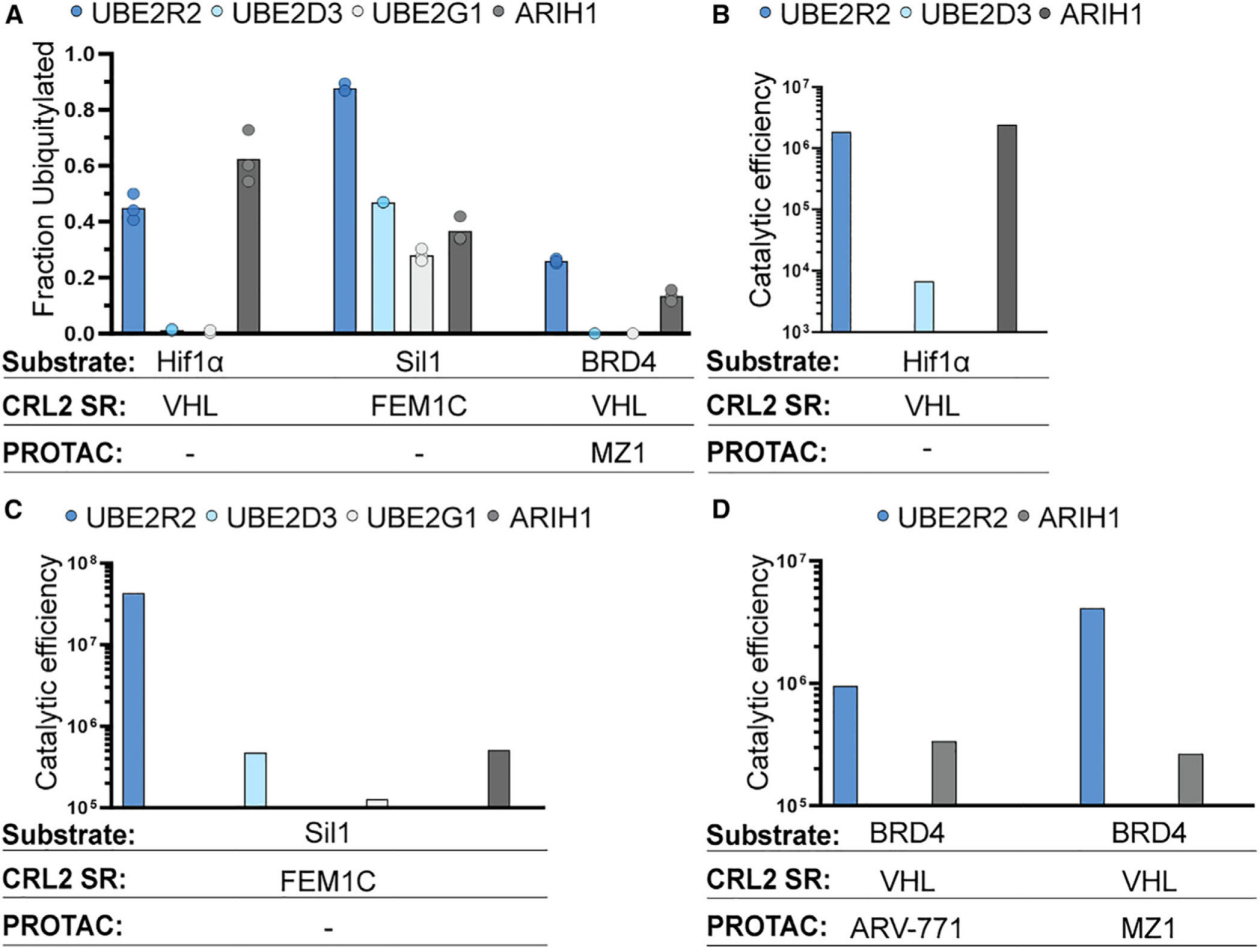
CRL2s selectively function with UBE2R2 in comparison with other UCEs (A) Graph showing the fractions of Hif1α peptide, Sil1 peptide, or recombinant BRD4 neo-substrate (spanning residues 346–460) ubiquitylated in reactions with UBE2R2 (blue), UBE2D3 (light blue), UBE2G1 (silver), or ARIH1 (gray). Reactions with Hif1α and Sil1 peptides contained neddylated CRL2^VHL^ and neddylated CRL2^FEM1C^, respectively, whereas BRD4 was recruited to neddylated CRL2^VHL^ by the PROTAC MZ1. (B) Bar graph comparing the catalytic efficiencies of UBE2R2, UBE2D3, and ARIH1 for Hif1α peptide substrate ubiquitylation. Catalytic efficiency (M^−1^s^−1^) is the ratio of the average values of *k*_*obs*_ and *K*_*m*_. (C) Same as in (B), but with Sil1 peptide substrate and neddylated CRL2^FEM1C^. (D) Same as in (B), but with BRD4 (346–460) neo-substrate and neddylated CRL2^VHL^ recruited by PROTACs ARV-771 or MZ1. All experiments from (A) to (D) were completed in triplicate technical replicates. See also Figure S1 and Table S1.

Since CRL2s have not been examined for UCE preference, we tested representative members from ARIH, UBE2D, UBE2G, and UBE2R families for ubiquitin priming of substrates recruited to various neddylated E3s: Hif1α peptide with CRL2^VHL^, Sil1 C-degron peptide with CRL2^FEM1C^, and PROTAC-mediated BRD4 neo-substrate with CRL2^VHL^. Unexpectedly, the results strikingly differed from expectations established from the studies of other CRLs,^34,37,38,43,62^ in that UBE2R2 was superior in a majority of cases (Figure 1A).

To further explore potential preferences of CRLs for UCEs, the rates of ubiquitin priming of substrate, *k_obs_*, were estimated using pre-steady-state kinetic ubiquitylation assays. The Michaelis constant (*K_m_*) values of UCEs for CRL complexes may also inform on specificity as an apparent binding constant. Indeed, the ratio of *k_obs_* and *K_m_* is related to *k_cat_*/*K_m_* (aka catalytic efficiency, M^−1^s^−1^), which is also referred to as a specificity constant.

In contrast to CRL1 E3s, the catalytic efficiencies of UBE2R2 and ARIH1 were comparable for CRL2^VHL^-dependent ubiquitin priming of Hif1α peptide (Figures 1B and S1A; Table S1). Consistent with these results, we previously had shown that the ablation of UBE2R1 and UBE2R2 in HEK293T cells resulted in the stabilization of HIF1α protein.^37^ UBE2R2 also forged chains onto primed Hif1α peptide with millisecond kinetics (Figures S1B–S1D; Table S1), also consistent with UBE2R2’s established function of rapid poly-ubiquitin chain formation onto primed CRL1-bound substrates.^37,60^ On the other hand, UBE2D3—widely used to prime RING E3 substrates—showed a 270-fold lower efficiency for the same CRL2^VHL^-substrate complex.

The kinetics with other CRL2s and substrates revealed a consistent, astonishing preference for UBE2R2 over the other UCEs. For instance, the catalytic efficiency of UBE2R2 was 80-fold greater for the neddylated CRL2^FEM1C^-Sil1 peptide complex in comparison with ARIH1, and even greater preferences were observed relative to UBE2D3 and UBE2G1 (Figures 1C, S1E, and S1F; Table S1).

The CRL2 trend for priming substrates with UBE2R2 was not limited to natural substrates but also extended to PROTAC-dependent targeting of a neo-substrate of neddylated CRL2^VHL^. For instance, the catalytic efficiency of UBE2R2 for MZ1-mediated BRD4 substrate priming^54^ was nearly 20-fold greater than with ARIH1 (Figures 1D and S1G). Interestingly, the distinct PROTAC ARV-771 (that differs with MZ1 primarily in the linker region between the neo-substrate-targeting warhead and CRL-binding component)^52^ led to a more modest 3-fold advantage for UBE2R2 (Figures 1D and S1H). In summary, although ARIH1, as well as in a single case UBE2D3,^37–39^ display substrate priming efficiencies that are far greater than UBE2R2 with CRL1s, the CRL2s tested are typically more efficient with UBE2R2 compared with the other UCEs.

### Functional linkage between UBE2R-family UCEs and CRL2s in cells

To query functional connection between UBE2R E2s and specific E3s, we probed the entire cellular system of CRLs for auto-degradation. Briefly, among the elaborate mechanisms enabling cells to adapt the CRL system as needed for cellular regulation, many CRLs auto-ubiquitylate and promote degradation of their constituent substrate receptors in the absence of substrates or neo-substrates.^63,64^ Thus, changes in substrate receptor abundance are detected at a total proteome level when ubiquitylation by a given CRL is perturbed.^65,66^ Accordingly, if CRL-UCE specificity existed and was non-redundant, the genetic ablation of the UCE would relieve substrate receptor auto-degradation and result in increased levels in knockout cells.

**Figure 2.**
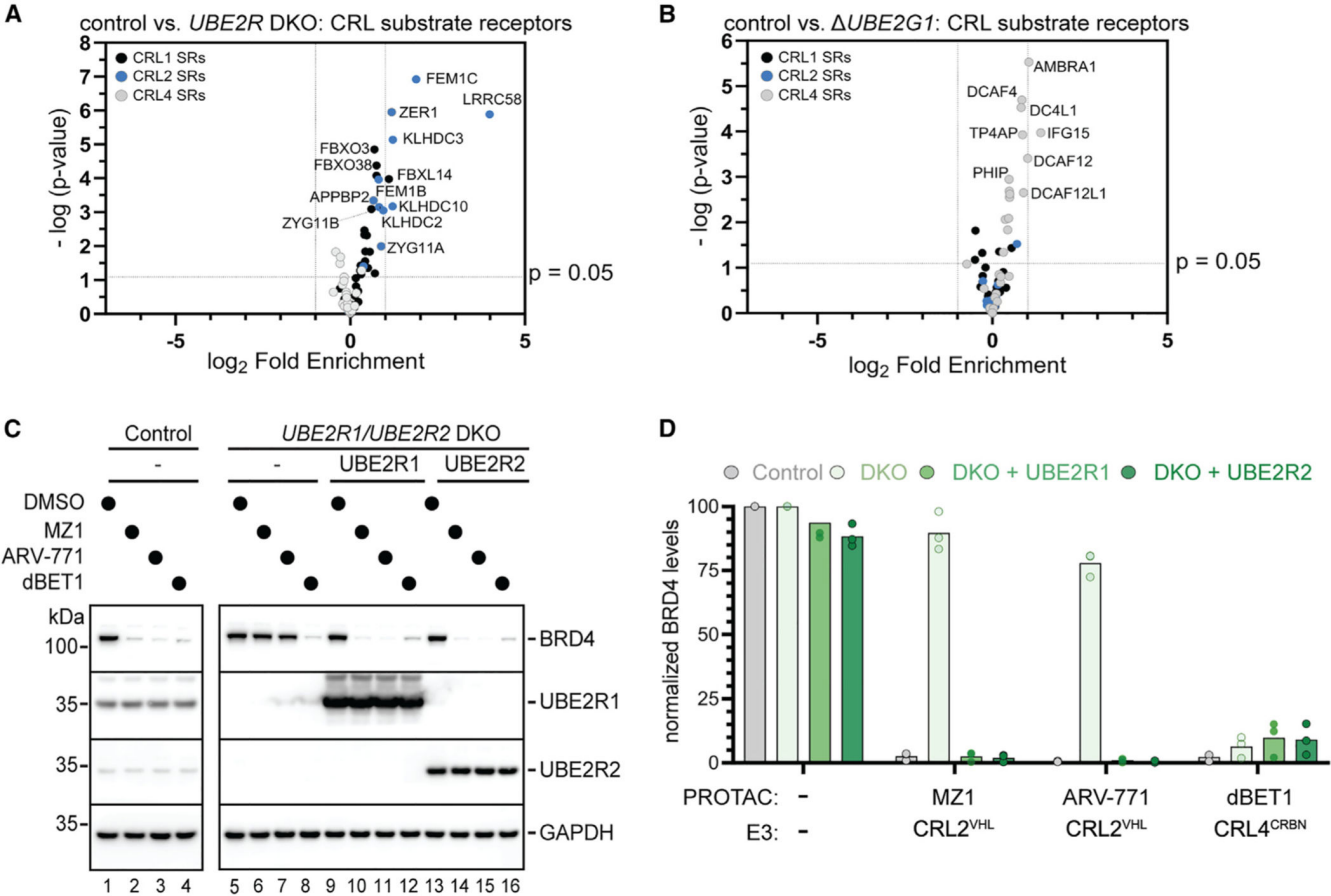
CRL2s employ UBE2R1 and UBE2R2 in human cells for both substrate receptor auto-regulation and efficient neo-substrate degradation (A) Selective volcano plot showing the stabilities of CRL substrate receptor proteins (for CRL1s in black, CRL2s in blue, and CRL4s in gray) in *UBE2R1*/*UBE2R2* double knockout (DKO) cells relative to control. (B) Same as in (A), but for *UBE2G1* knockout cells. Triplicate biological samples were analyzed for each comparison. (C) Representative western blots comparing protein levels upon treatment with PROTACs in control or *UBE2R1*/*UBE2R2* DKO HEK293T cells. Notice that ectopic expression of either UBE2R1 (lanes 9–12) or UBE2R2 (lanes 13–16) completely restored BRD4 degradation with MZ1 or ARV-771. (D) Graph of the BRD4 protein levels as shown in (C). The matching CRL (E3) is shown for each PROTAC. Datapoints reflect triplicate technical replicates performed using control (clone D5) and DKO (clone A10) cell lines. See also Figure S2 and Excel File S1.

The levels of proteins from either control or *UBE2R1*/*UBE2R2* double knockout (DKO) HEK293T cells were compared by global proteomics and tandem mass tagging (TMT) liquid chromatography-mass spectrometry (LC-MS). 121 proteins were identified with statistical significance and levels that were at least 50% higher in the DKO cells relative to control (p < 0.05; Figure S2A; Excel File S1). Nine of the topmost 10 stabilized CRL substrate receptors belonged to CRL2s, whereas only five CRL1 and no CRL4 substrate receptors were among the 121 proteins (Figure 2A).

Since it had been reported that UBE2G1 functions with a CUL4-based CRL in combination with small molecule inducers of TPD,^34,35^ global proteomics were again performed comparing *UBE2G1* knockout with control cells to further explore potential CRL-UCE specificity (Figure S2B; Excel File S1). In stark contrast with the results for *UBE2R1/UBE2R2* DKO cells, seven of the eight CRL substrate receptors with levels that were at least 50% higher in *UBE2G1* knockout cells belonged to the CRL4 subfamily, with only one CRL2 substrate receptor also being identified (Figure 2B). In summary, comparing the cellular protein levels between control and UCE knockout cells suggested physiological CRL pairings between UBE2R-family E2s and CRL2s and between UBE2G1 and CRL4s.

### UBE2R-family E2s are necessary for efficient neo-substrate degradation by CRL2-dependent degraders

An exciting function of CRL2s is their applicability to TPD.^4–9^ The strong cellular and biochemical link between UBE2R2 and CRL2s prompted our investigation into whether PROTAC-mediated protein degradation is also sensitive to the presence of UBE2R1 and UBE2R2 proteins in cells. PROTACs ARV-771^52^ and MZ1^54^ were selected since they employ CRL2^VHL^ for ubiquitylation of the neo-substrates BRD2–4, whereas dBET1 was chosen as a negative control due it targeting the same neo-substrates but through a CRL4.^53^

Although all three PROTACs led to the complete degradation of BRD2–4 in control HEK293T cells, neo-substrate levels in *UBE2R1/UBE2R2* DKO cells persisted despite treatment with ARV-771 or MZ1 (Figures 2C, 2D, and S2C–S2F). The effect was highly specific for CRL2s as dBET1 treatment still resulted in efficient BRD2–4 degradation. Ectopic expression of UBE2R1 or UBE2R2 in the DKO cells both restored PROTAC-dependent BRD2–4 degradation and reversed stabilization of two CRL2-dependent substrate receptor proteins that had been identified in this study by proteomics (Figure S2G). On the other hand, the small interfering RNA (siRNA)-mediated knockdown of ARIH1 had no effect on the degradation of BRD4 (Figure S2H).

**Figure 3.**
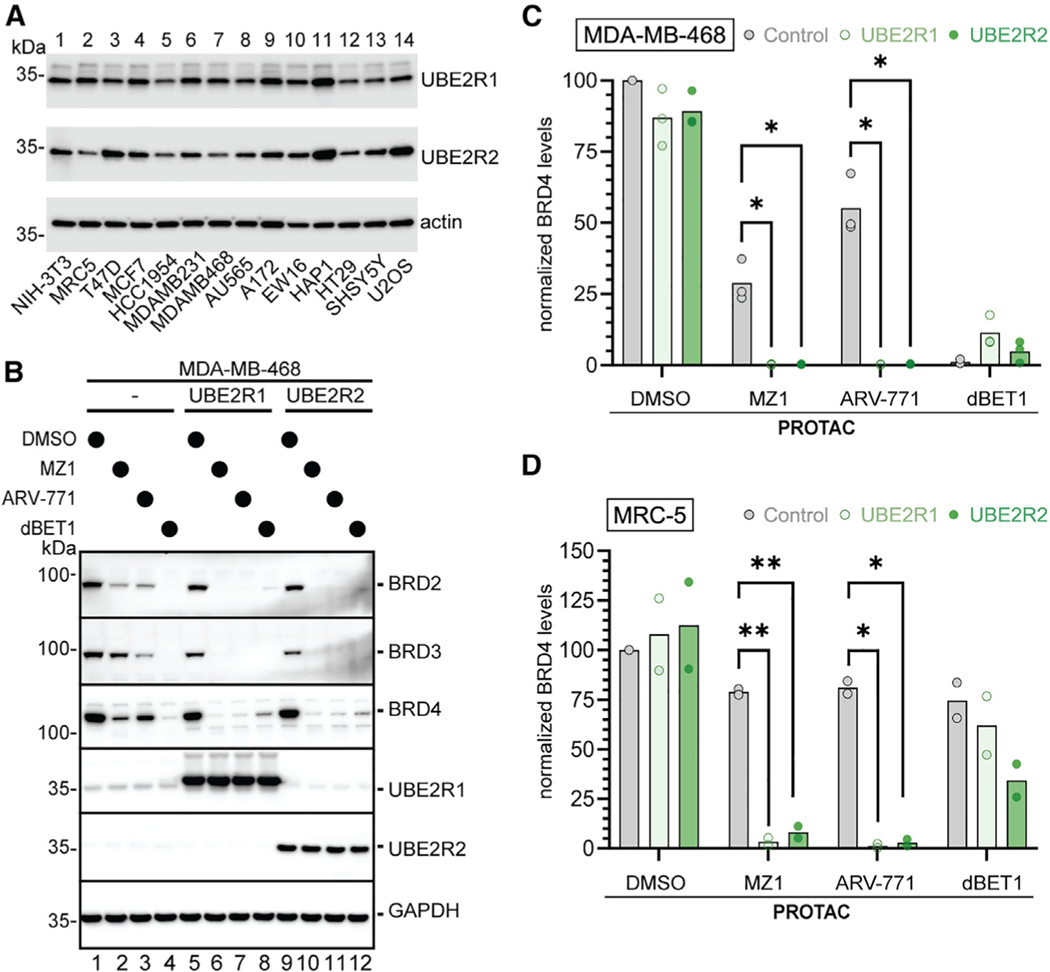
Lower UBE2R2 expression in cells results in less efficient neo-substrate degradation with CRL2-dependent PROTACs (A) Representative Western blots showing UBE2R1 and UBE2R2 protein levels from the indicated cell lines. (B) Representative Western blots showing the indicated protein levels in the model breast cancer cell line MDA-MB-468 that had been treated with the indicated PROTACs or DMSO and upon ectopic expression of UBE2R1 or UBE2R2. (C) Graphical representation of BRD4 levels from (B). *p < 0.05 represents the statistical significance of the indicated comparisons as derived by an unpaired t test with Welch’s correction. Datapoints reflect triplicate technical replicates performed on the same cell line. (D) Same as in (C), except with MRC-5 cells. *p < 0.05 and **p < 0.01 represent the statistical significance of the indicated comparisons as derived by an unpaired t test with Welch’s correction. Datapoints reflect duplicate technical replicates performed on the same cell line. See also Excel File S2.

Could endogenous UBE2R1 and UBE2R2 levels impact PROTAC efficacy? We examined a panel of cell lines for their steady-state levels of UBE2R1 and UBE2R2 and noticed low levels of UBE2R2 in multiple cell lines including the breast cancer cell line MDA-MB-468 (Figure 3A; Excel File S2). Although treatment of MDA-MB-468 cells with dBET1 led to complete loss of BRD2–4, neo-substrate levels persisted upon treatment with MZ1, particularly BRD3 (Figures 3B, 3C, S2I, and S2J). Interestingly, treatment with ARV-771 also resulted in less efficient neo-substrate elimination in comparison with dBET1, but here BRD4 levels remained to the greatest extent. Ectopic expression of either UBE2R1 or UBE2R2 resulted in increased clearance of neo-substrate from the cells. Further examination of an additional cell line (the non-immortalized human lung fibroblast line MRC-5) with relatively low UBE2R2 levels similarly showed enhanced degrader-induced loss of BRD2–4 upon ectopic expression of UBE2R1 or UBE2R2 (Figures 3D and S2K–S2M).

### Preferred catalytic geometry for CRL2-bound substrates by UBE2R2

**Figure 4.**
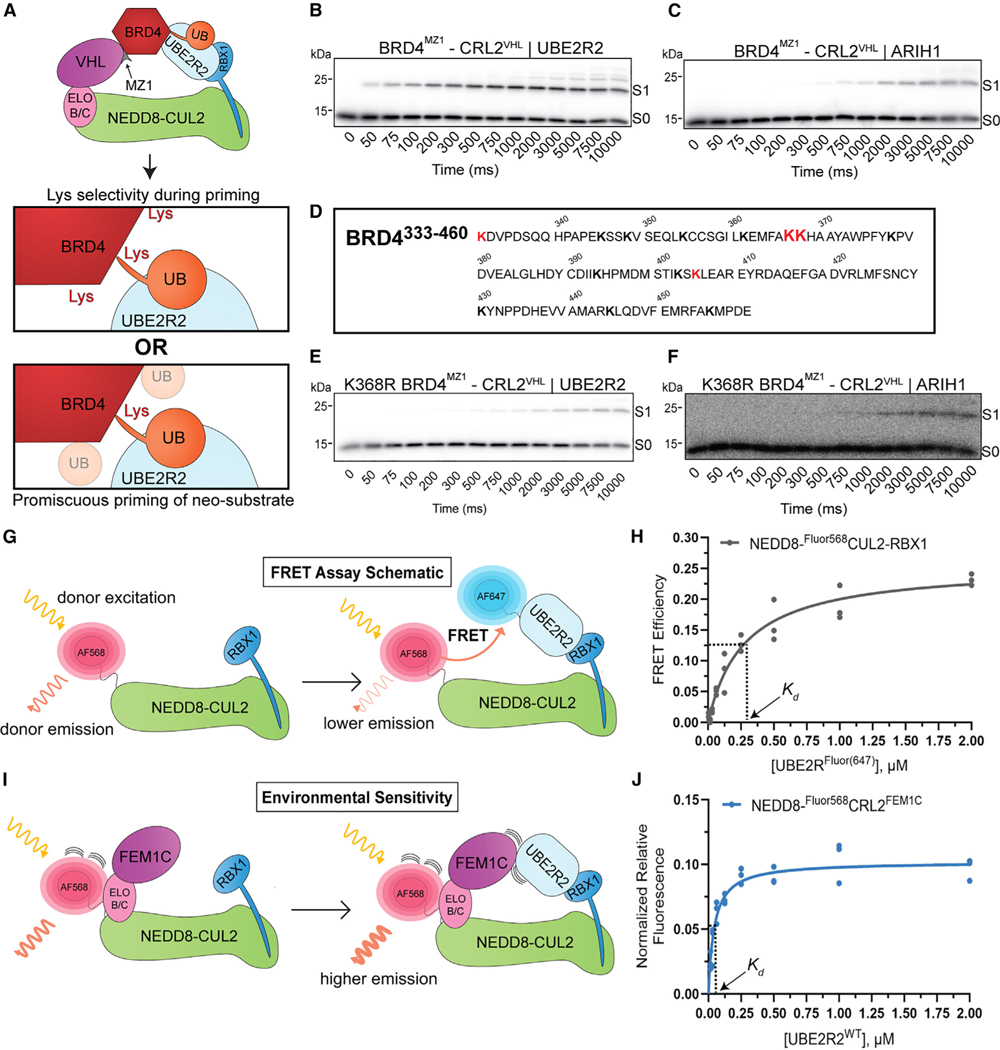
Preferred catalytic geometry for CRL2-bound substrate by UBE2R2 (A) Schematic illustrating *in vitro* reconstituted BRD4 neo-substrate ubiquitylation in the presence of neddylated CRL2^VHL^, UBE2R2 (light blue), and the PROTAC MZ1 (gray). Notice that the recombinant BRD4 protein contains multiple Lys residues (the actual number has been reduced for simplicity) that potentially may serve as sites of ubiquitin (UB; orange) priming of neo-substrate. (B) Representative autoradiogram showing a pre-steady-state ubiquitylation reaction time course with UBE2R2, MZ1, and WT BRD4 (346–460) neo-substrate. S0 represents unmodified substrate, whereas S1 is product containing a single ubiquitin. (C) Same as (B), except with ARIH1. (D) Amino acid sequence diagram of recombinant BRD4 neo-substrate containing residues 333–460. Lys residues identified as ubiquitylated by mass spectrometry have been highlighted red, with increased font size showing the top hits (367 and 368). (E) Same as (B), except with K368R BRD4. (F) Same as (E), except with ARIH1. (G) Schematic illustrating how fluorescence resonance energy transfer (FRET) is achieved between two fluorophores conjugated to neddylated CUL2-RBX1 and UBE2R2. Excitation of Alexa Fluor 568 on neddylated CUL2-RBX1 produces fluorescence emission that is sensitive to the presence of UBE2R2 conjugated to Alexa Fluor 647. (H) Graph of the FRET efficiency versus labeled UBE2R2 titration in the presence of fluorescent neddylated CUL2-RBX1 and reaction buffer at ionic strength of 50 mM. The data were fit to a one-site binding model by nonlinear regression (GraphPad Prism software v10). (I) Schematic depicting the environmental sensitivity of Alexa Fluor 568 on neddylated CUL2-RBX1 to substrate receptor and UBE2R2. Binding of the FEM1C substrate receptor complex increases fluorescence (left), whereas the addition of UBE2R2 further increases fluorescence, possibly through the direct interaction of UBE2R2 with FEM1C (right). (J) Graph showing the normalized change in neddylated ^Fluor568^CRL2^FEM1C^ fluorescence upon the titration of unlabeled UBE2R2 in reaction buffer at ionic strength of 50 mM. All kinetics and binding experiments were completed in triplicate technical replicates. See also Figure S3 and Table S2.

To gain mechanistic insight into UBE2R2 preference for CRL-dependent substrate ubiquitylation, we pursued two striking observations. First, the *in vitro* reconstituted ubiquitylation reaction with BRD4 showed apparent Lys specificity of neo-substrate priming for both UBE2R2 and ARIH1, despite the recombinant BRD4 fragment containing 14 solvent-exposed Lys residues (Figures 4A–4C). To identify the Lys residue that was being modified by UBE2R2, *in vitro* ubiquitylation reactions were performed followed by MS (Figures 4D and S2N). Although several Lys residues were targeted, the results were consistent with Lys368 being ubiquitylated to the greatest extent. Consistent with this result, modification of K368R BRD4 by UBE2R2 was greatly reduced with reactions containing MZ1 and ARV-771 (Figures 4E and S1I; Table S1). The specificity with UBE2R2 was unexpected because, presently, the notion is that CRLs target a zone that typically encompasses multiple potential ubiquitylation sites. On the other hand, the same CRL2 complex with ARIH1 primed both wild-type (WT) and K368R BRD4 with similar kinetics (Figures 4C, 4F, S1H, and S1I; Table S1).

Second, among the CRLs tested, CRL2^FEM1C^ stood out for its specificity for a UBE2R-family E2 for substrate priming *in vitro* and for auto-degradation in cells (Figures 1C and 2A, respectively). This raised the possibility that not only a cullin-RING complex but also a substrate receptor could directly impact recruitment of a UCE. To test this hypothesis, we developed a fluorescence resonance energy transfer (FRET) binding assay (Figures 4G and S3A–S3C). As a control, we tested effects of ionic strength of the buffer solution on the *K_d_* of UBE2R2 for neddylated CUL2-RBX1, as electrostatic interactions between UBE2R2’s acidic C-terminal tail have been shown to be a key determinant of cullin binding^67^ (Figures 4H and S3D; Table S2). Formation of the active CRL, through the addition of ELONGIN B/C-FEM1C to neddylated CUL2-RBX1, decreased the *K_d_* of UBE2R2 for the CRL complex by approximately 7-fold (Figures 4J and S3G; Table S2; here environmental perturbation was employed instead of FRET, please see STAR Methods and Figures 4I and S3E). Appending a ubiquitin to the UBE2R2 active site had no effect on the *K_d_* value (Figure S3H; Table S2), consistent with a previous study reporting the affinity of UBE2R2 for a neddylated CRL1.^33^

### An expansive interface between UBE2R2 and the neddylated CRL2^FEM1C^ complex promotes CRL-UCE specificity

**Figure 5.**
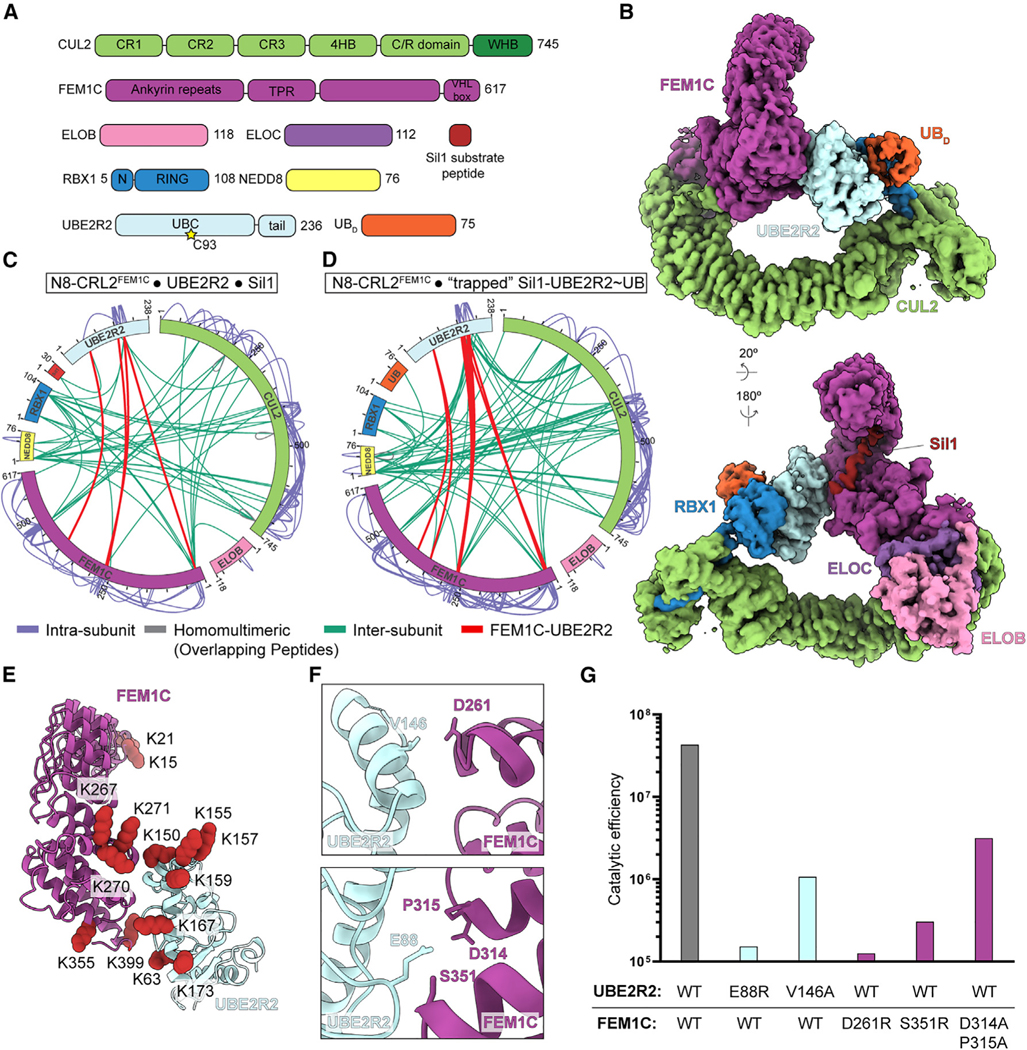
Optimal UBE2R2 activity is achieved by geometric optimization with the CRL substrate receptor (A) Guide to coloring of the various subunits within the neddylated CRL2^FEM1C-Sil1^-UBE2R2~ubiquitin cryo-EM structure. C93 (yellow star) represents the active site Cys residue that is thioesterified to donor ubiquitin. Notice that UBE2R-family members have a unique acidic tail located C-terminal to the catalytic UBC domain. ELOB, ELONGIN B; ELOC, ELONGIN C; UB_D_, ubiquitin. (B) DeepEMhancer consensus cryo-EM map representing the activated conformation of neddylated CRL2^FEM1C^ bound to Sil1 peptide covalently joined to a stable proxy for UBE2R2~ubiquitin. (C) Diagram showing intra- (light purple) and inter-subunit (green) cross-linking of the neddylated (N8) CRL2^FEM1C^ complex in the presence of UBE2R2 and Sil1 peptide. Cross-links between FEM1C and UBE2R2 have been colored red. (D) Same as (C), except with the trapped Sil1 peptide-UBE2R2~ubiquitin complex. (E) Ribbon diagram showing the location of cross-linked FEM1C and UBE2R2 Lys residues (red spheres) based on the results in (D), shown on previous structures (PDB: 6LBN and PDB: 6NYO, respectively) docked into the cryo-EM map shown in (B). (F) Ribbon diagrams showing the location of residues at the interface between FEM1C (purple) and UBE2R2 (light blue). (G) Bar graph comparing the catalytic efficiencies of wild-type (WT) components with either mutant variants of UBE2R2 (light blue) or FEM1C (purple). See also Figures S4 and S5 and Tables 1 and S1.

Prior studies had suggested that cullin binding to substrate receptors and RING binding to UCEs are independent interactions separated by nearly 100 Å across the length of a cullin.^68^ Moreover, mechanistic and structural studies have led to the view that UCEs with intrinsic linkage-specific chain-forming activity such as UBE2R2 are specific for transferring ubiquitin to another ubiquitin.^36,43,45–47,59,69,70^ Thus, to understand how a substrate receptor could impact the binding and activity to drive UBE2R-family E2 priming of neddylated CRL2^FEM1C^ substrates, we obtained cryoelectron microscopy (cryo-EM) data for a stable complex mimicking this reaction. To obtain a proxy for the assembly catalyzing substrate priming (Figure S4A), ubiquitin’s Gly75 was first cross-linked to a Cys replacement for an acceptor Lys on Sil1 peptide, which was then reacted with UBE2R2’s active site Cys in the presence of neddylated CRL2^FEM1C^ (Figure S4B; importantly, nonspecific cross-linkers were not used during sample preparation that could bias the structure). The map, at overall 3.7 Å resolution, allowed clear placement of previous structures into the cryo-EM density and iterative rounds of model building (with side chains when visible in the cryo-EM maps) and refinement (Figures 5A, 5B, and S4C–S4F; Table 1; Video S1).

The UBE2R2-mediated priming structure showed two striking features. First, comparison to the only other structure representing a CRL-E2 complex ubiquitylating a substrate—neddylated CRL1^FBXW1^ with well-established UBE2D-family priming enzymes^38^—showed that, despite some shifting relative to the cullin C-terminal region, both E2~ubiquitins display their active sites toward the substrate receptors (Figure S5A). Second, the substrate receptor—FEM1C—makes extensive, direct contacts with the UCE UBE2R2 (Figure 5B). Loops emanating from all three FEM1C tetratricopeptide repeat (TPR) motifs converge at the interface with UBE2R2, which itself contributes a helix-turn-helix element located at the C-terminal end of the catalytic UBC domain and a loop immediately distal to the central β-sheet to the interface. Interactions at this helix-turn-helix element have been shown to stimulate intrinsic—albeit linkage-specific chain forming—activities of other E2s.^69,71,72^

These observations raise the possibility that substrate receptors can toggle the ubiquitylation of their targets. First, cross-linking MS^73^ was performed, which showed extensive cross-linking between UBE2R2 and FEM1C that agreed with the structural interface (Figures 5C–5E and S5B). Second, mutagenesis of residues located at the FEM1C-UBE2R2 interface (Figure 5F) resulted in substantial defects in ubiquitylation without generally affecting enzyme activity. For instance, the catalytic efficiency of E88R UBE2R2 was reduced nearly 300-fold (Figures 5G, S5C, and S5D; Table S1), whereas E88R UBE2R2 activity was not compromised with neddylated CRL2^VHL^ and Hif1α peptide substrate (Figure S5E). The mutation of UBE2R2 residue Val146 also resulted in defects in activity that were specific for the neddylated CRL2^FEM1C^ complex (Figures 5G and S5C–S5E). Mutagenesis of multiple FEM1C residues located at the interface also led to significant disruptions in UBE2R2-dependent Sil1 priming without affecting ARIH1 activity (Figures 5G and S5F–S5H). In summary, the cryo-EM structure of UBE2R2 bound to CRL2^FEM1C^ illuminates a unique interface between UBE2R2 and FEM1C that is required for rapid priming kinetics of a substrate recruited to this particular CRL-UCE pairing.

**Figure 6.**
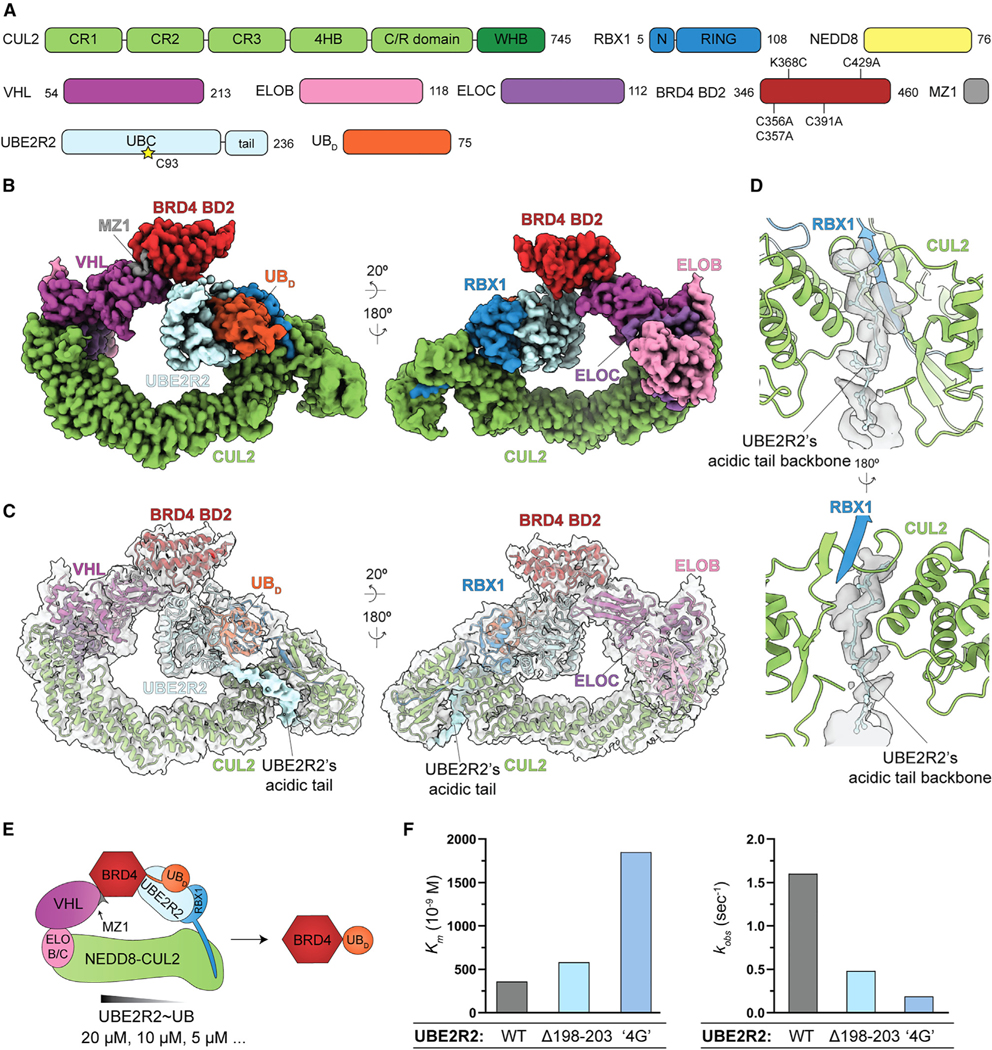
Anchoring of the UBE2R2~ubiquitin-RING catalytic core by interaction of the unique UBE2R2 C-terminal tail with CUL2 (A) Guide to coloring of the various subunits within the neddylated CRL2^VHL-MZ1-BRD4 (346–460)^-UBE2R2~ubiquitin cryo-EM structure. BD2 represents the second bromodomain of the BRD4 protein spanning residues 346–460. The UBE2R2 active site Cys residue is denoted as C93 (yellow star). UBE2R-family members have a highly conserved, acidic tail located at the C terminus not present in other human E2s. ELOB, ELONGIN B; ELOC, ELONGIN C; UB_D_, ubiquitin. (B) DeepEMhancer consensus cryo-EM map representing the activated conformation of neddylated CRL2^VHL^ bound to K368C BRD4 (346–460) covalently joined to a stable proxy for UBE2R2~ubiquitin and with the PROTAC MZ1. (C) Ribbon diagram superimposed with the unsharpened cryo-EM consensus map (gray) of the neddylated CRL2^VHL^ structure bound to BRD4 (346–460), UBE2R2~ubiquitin, and the PROTAC MZ1. Notice the presence of a continuous tube of density in the maps (light blue) corresponding to UBE2R2’s C-terminal acidic tail. (D) DeepEMhancer focused cryo-EM map on the interaction of UBE2R2’s acidic tail with the CUL2 basic canyon region. The backbone atoms (light-blue ball-and-sticks) corresponding to 15 consecutive acidic tail residues and their fit to the density are highlighted. Secondary structure elements corresponding to the CUL2 basic canyon (green) and RBX1’s N-terminal β strand (blue) are shown. (E) Schematic illustrating *in vitro* reconstituted, BRD4 neo-substrate ubiquitylation and estimation of the *K*_*m*_ of UBE2R2 for the CRL2 complex. Neddylated CRL2^VHL^, the PROTAC MZ1 (gray), and various concentrations of UBE2R2~ubiquitin (light blue and orange, respectively) were mixed to estimate the fraction of ubiquitin-primed substrate as a function of the UBE2R2 concentration. (F) Bar graphs comparing the *K*_*m*_ (left) or *k*_*obs*_ (right) values of wild-type (WT) or UBE2R2 acidic tail mutants for neddylated CRL2^VHL^. Notice that deletion of residues 198 to 203 (Δ198–203) or a “4G” quadruple point mutant (L206G Y207G L210G Y211G) resulted in both a decrease in the apparent affinity of the mutants for the CRL2 complex as well as reductions in the rates of BRD4 neo-substrate priming. See also Figures S6, S7A, and S7B and Tables 1 and S1.

To assess the structure of UBE2R2-mediated neo-substrate priming, cryo-EM was performed on a UBE2R2~ubiquitin mimic cross-linked to a recombinant BRD4 fragment recruited to the neddylated CRL2^VHL^ by the PROTAC MZ1^74^ (Figure 6A; Table 1; Video S2). Similar to the neddylated CRL2^FEM1C^ priming structure, the map (3.4 Å resolution) allowed for the placement of previous structures (Figures 6B, 6C, and S6A–S6D). The refined map also showed continuous, tube-like electron density emanating at the C terminus of UBE2R2’s UBC domain and traversing into CUL2’s canyon region (Figure 6C). We reasoned that the density corresponded to UBE2R2’s unique C-terminal tail,^75–78^ important for processive poly-ubiquitin chain formation.^67,79^ Visualization of the tail was somewhat unexpected since previous studies suggested nonspecific electrostatic interactions between the tail and cullin subunits,^67,80^ and density was not apparent in the neddylated CRL2^FEM1C^ priming structure or those representing UBE2R2-mediated poly-ubiquitin chain formation.^81^ Although the density was less visible after post-processing, these maps still enabled assignment of the backbone atoms for 15 UBE2R2 tail residues, illuminating interaction with CUL2 (Figure 6D). Two UBE2R2 mutants, one that internally shortened the tail by six residues and another that replaced four hydrophobic residues, corresponding to those previously shown as important for UBE2R1 activity with a CRL1 E3,^77^ displayed increased *K_m_* values of UBE2R2 for the neddylated CRL2^VHL^ complex as well as reduced *k_obs_* values for ubiquitin transfer to BRD4 (Figures 6E, 6F, S7A, and S7B).

### Dynamic subunit assembly enables both UBE2R2-mediated substrate priming and poly-ubiquitin chain extension

**Figure 7.**
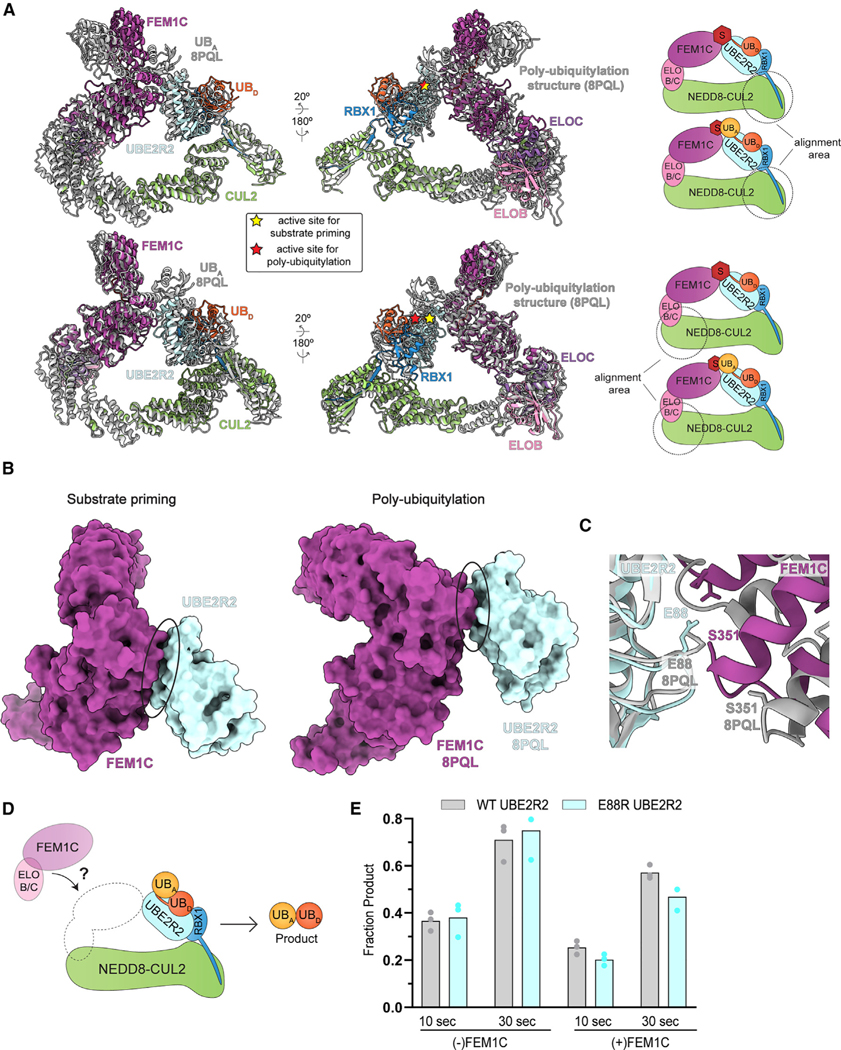
Striking conformational flexibility illustrated by UBE2R2-mediated substrate priming and poly-ubiquitin chain extension (A) Superposition of the neddylated CRL2^FEM1C-Sil1^-UBE2R2~ubiquitin-substrate priming structure reported here and the cryo-EM poly-ubiquitin chain extension structure (gray; PDB: 8PQL). Coloring of the subunits in the priming structure is defined in Figure 5A. Donor ubiquitin (UB_D_) is activated by UBE2R2 for priming of unmodified substrate or chain extension by attachment to acceptor ubiquitin (UB_A_). The UBE2R2 active sites of the priming and chain extension structures have been noted by stars (yellow and red, respectively). The schematics (right) indicate the regions that were used to perform the structural alignments. (B) Space-filling diagrams comparing the priming and chain extension intermolecular interfaces between UBE2R2 (light blue) and FEM1C (purple). Whereas approximately 400 Å^2^ of surface area is buried between UBE2R2 and FEM1C in the priming structure, conformational changes have shifted the poly-ubiquitylation interface such that only ≈200 Å^2^ of surface area is buried. (C) Structural superposition of the UBE2R2-FEM1C interfaces comparing the priming and chain extension structures (ribbon diagrams). Notice that UBE2R2’s Glu88 is in proximity with FEM1C’s Ser351 in the priming structure, whereas the same residues are substantially shifted in the chain extension one. (D) Diagram illustrating an assay for UBE2R2’s di-ubiquitin chain formation activity in the presence of unanchored acceptor ubiquitin and comparison with either neddylated CUL2-RBX1 or neddylated CRL2^FEM1C^ complex. UB_D_, donor ubiquitin; UB_A_, acceptor ubiquitin; ELO B/C, ELONGIN B/C. (E) Bar graph comparing CRL-stimulated di-ubiquitin product formation by wild-type (WT) or E88R UBE2R2 in the absence or presence of the ELONGIN B/C-FEM1C substrate receptor complex. See also Figure S7.

We compared the neddylated CRL2^FEM1C^ structures showing UBE2R2-mediated priming and poly-ubiquitin chain formation. Aligning the two structures showed subtle rotation of subunits located at either end of CUL2 (Figure 7A). On one side, the RBX1 RING-UBE2R2~ubiquitin catalytic assembly rotates by 7° about RBX1’s flexible linker, which results in a relative translation of the UBE2R2 active site by nearly 5 Å (Figure S7C). An 8° difference about CUL2’s central cullin repeat results in a 17 Å translation in the position of FEM1C’s N terminus (Figure S7D). Comparison of the FEM1C conformations for priming and extending revealed a 3° rotation across the atomic positions, resulting in a 5–10 Å translation of the surfaces most adjacent to UBE2R2 (Figure S7E). Altogether, multiple subtle yet significant changes to the structures would allow targeting of a variety of CRL2 substrates but with strict geometric conditions.

Comparing the structures also illuminated stark differences for priming and poly-ubiquitin chain extension^81^ (Figures 6B and 7A). For instance, relative rearrangement of both UBE2R2 and FEM1C, presumably occurring after linkage of the first ubiquitin, resulted in 50% reduction of buried surface area at the interface in comparison with the priming structure (Figure 7B). For example, UBE2R2’s Glu88—which forms a key contact with FEM1C’s Ser351 in the complex representing substrate priming (Figure 5F)—no longer contacts FEM1C in the structure representing poly-ubiquitylation (Figure 7C). Indeed, unlike effects on substrate priming (Figure 5G), assaying for unanchored poly-ubiquitin chain formation demonstrated that E88R UBE2R2 shows WT-like activity (Figures 7D and 7E). Furthermore, the chain formation structure highlighted the presence of a synergy loop on the UBE2R2 UBC domain that physically connects the CRL RING domain with the acceptor ubiquitin bound to substrate to promote rapid poly-ubiquitin chain formation^81^ (Figure S7F). The loop appeared to be disordered in the neddylated CRL2^FEM1C^ priming structure owing to a lack of density in the cryo-EM maps (Figure S7G), whereas loop density was apparent for the BRD4 priming structure (Figure S7H).

## DISCUSSION

In the 30 years since CRLs were shown to be key mediators of protein ubiquitylation, substrate targeting has largely been attributed to binding by cognate receptors without substantial consideration of involvement of any particular enzyme catalyzing ubiquitylation. This concept had been even further solidified by PROTAC design focusing on generating a stable interface between the CRL substrate receptor and neo-substrate.^7,74,82,83^ The discovery of numerous UCEs functioning with neddylated CRLs in humans suggested substantial redundancy for either substrates or neo-substrates^30–37^: any of the various UCEs seemed equipped to promote ubiquitylation, at least for several CRL1 substrates. Thus, discovery of specific UCEs as contributing to CRL function for targeting specific substrates (Figure 1) was unexpected.

Even more surprisingly, given that the majority of CRLs harbor a common UCE-binding RING domain (from RBX1), our data reveal a correlation between cullin identity and UCE selection. The results showed connections between CRL2 E3s either as a whole or for particular family members with UBE2R-family E2s at all possible levels: (1) kinetics of substrate and neo-substrate ubiquitylation *in vitro*, (2) auto-regulation of CRL2 levels in cells, (3) PROTAC-mediated neo-substrate degradation, (4) site-specific ubiquitylation, and (5) E3-E2 affinity.

These findings have important implications for not only endogenous regulation but also for developing therapeutics mediating TPD.^4–9,53,61,84^ Some degrader drugs are already front-line therapies,^4,85^ and multiple new candidates are now in clinical trials.^86^ In cases where the identity of the E3 ligase has been disclosed, TPD typically involves a CRL, with CRL2^VHL^ employed in at least one of those cases.^86^ Notably, differences in levels of UBE2R2 affected the efficiency of neo-substrate degradation elicited by multiple CRL2-targeting PROTACs from three distinct cell lines (Figures 2 and 3). Future studies may be employed to ascertain whether additional cell lines will show correlation of UBE2R1 and/or UBE2R2 levels and CRL2-targeting PROTAC efficiency (Figure 3A).

Our data showing neddylated CRL2-based E3s preferentially employing UBE2R-family E2s provides a rationale for the previous identification of UBE2R2 in a CRISPR screen designed to discover effectors of a CRL2^VHL^-harnessing degrader.^87^ Although prior studies seeking to identify E2s functioning with CRL4^CRBN^ and CRL4^DCAF15^ identified a key role for the chain-elongating E2 UBE2G1,^34,35,87^ our data for CRL auto-regulation suggest this UCE is broadly employed by CUL4-based CRLs (Figure 2B). Furthermore, the striking target lysine specificity for MZ1-mediated ubiquitylation is consistent with strong geometric constraints imposed by the CRL2-UBE2R-family E2 partnership. Thus, our data suggest the importance of factoring in the features imposed by a CRL’s preferred UCE partner(s) during drug design to obtain the most efficacious degrader molecules. Accordingly, it also seems likely that UCE levels differ among patient samples and may be investigated for its potential as a diagnostic for which degrader molecules would be most effective.

It is remarkable that UBE2R E2s effectively prime CRL2 substrates, given their far weaker activity toward substrates of CRL1. In fact, the millisecond timescales for UBE2R-mediated poly-ubiquitylation of neddylated CRL1 substrates,^60^ combined with equally slow substrate priming, led to the model of distinct enzymes modifying substrate and extending a chain.^32^ Indeed, the delineation of substrate priming and poly-ubiquitin chain extension as separate tasks likely aided in the discovery of several other UCEs that rapidly prime CRL1 substrates.^30,31,37,40^ Moreover, given their proclivity to forge free ubiquitin chains even in the absence of an E3, it seems that UBE2R-family E2s are reprogrammed by CRL2 to also target substrates.

Future studies will be required to ascertain the molecular basis for differences between CUL2 and CUL1 in their employment of UBE2R-based E2s for substrate priming to CUL2. Nonetheless, a possible explanation emerges from the suite of structures showing neddylated CRLs in action. Not only RBX1 but also NEDD8 and CUL1 contact the UCEs (UBE2D E2s and ARIH1) priming CRL1 substrates. By contrast, neither NEDD8 nor its linked CUL1 domain (termed the WHB) are visible in cryo-EM maps for neddylated CRL1 or CRL2 complexes with UBE2R2, either during substrate priming (Figures 5B and 6B) or poly-ubiquitylation.^81^ Given the numerous arrangements a CRL’s WHB and RING domain must adopt—for neddylation,^88^ ubiquitylation,^38,39,81,89^ deneddylation,^14–16^ and binding to other factors^64^—it stands to reason that different cullin-RING complexes would have different intrinsic preferences for adopting the various conformations. As in other CRLs, these domains appear to sample many orientations in neddylated CUL2-RBX1. As such, we speculate that neddylated CUL2-RBX1 is relatively more poised to adopt conformations employed by UBE2R-family E2s.

CRL-UCE specificity also appears to manifest, at least in some cases, through direct interaction between the substrate receptor and the UCE, increasing the complex affinity and toggling faster kinetics of substrate priming by inducing substrate and UCE~ubiquitin juxtaposition (Figure 5). FEM1C drives proximity-induced degradation not only by recruiting a substrate to a CRL but also by co-recruiting and positioning a catalytically reprogrammed Lys48-linked chain forming E2. Nevertheless, it seems unlikely that UCE-substrate receptor interaction will be prerequisite for coordination of the CRL-bound substrate with the UCE active site. Indeed, the cryo-EM structure of neddylated CRL2^VHL^ bound to UBE2R2~ubiquitin with BRD4 and MZ1 shows a minimal interface between UBE2R2 and VHL in comparison with the neddylated CRL2^FEM1C^ priming structure. Although the N-terminal tail of the UCE ARIH2 was visualized binding the corresponding canyon of neddylated CUL5-RBX2,^89^ the observation of UBE2R2’s tail was surprising given prior studies suggesting highly dynamic binding between the tail and a cullin^67,80^ as well as multiple cryo-EM structures where density for the tail was absent (both this study and Liwocha et al.^81^). We suspect that the specific tethering of the tail in the CUL2 canyon may serve to restrict the conformation of the UBE2R2 UBC catalytic domain. Notably, UBE2D-family E2s lack a C-terminal tail and do not share conservation of the residues involved in the UBE2R2-FEM1C interface, perhaps explaining at least in part why these E2s are far less efficient at priming several CRL2 substrates compared with UBE2R2. Further uncovering of these striking structural features as well as the discovery of new ones may eventually facilitate design of degraders precisely placing a substrate’s lysine in the UBE2R2 active site. We propose that design elements of proximity-based drugs should focus not only on co-tethering the E3 and neo-substrate but also catalytic geometric activation that may facilitate therapeutic efficacy.

### Limitations of the study

Peptide or protein domain substrates have been used throughout this study owing to their homogeneity in composition, ease of chemical modification to form E3-guiding degrons, and incorporation of labels for their sensitive detection. Although desirable, full-length protein substrates are more complex for a variety of reasons, including the number of Lys residues that may be modified by ubiquitin, post-translational modifications, subcellular localization, and interaction with additional protein partners. These factors may result in challenges in producing highly pure samples necessary for the biochemical and structural methods employed here.

Also, cryo-EM performs best for complexes displaying homogeneous inter-subunit interactions. Since formation of the UBE2R2~ubiquitin-substrate traps is inefficient, the neddylated CRL complexes are expected to display at least some heterogeneity. Moreover, the final high-resolution reconstructions were performed with relatively small subsets of particles that were more homogeneous, suggesting that the structures may represent only the most stable arrangements. Nonetheless, the results obtained from UBE2R2 and FEM1C mutants support a critical role for the structurally observed interfaces (Figure 5).

## STAR★METHODS

### RESOURCE AVAILABILITY

#### Lead contact

Further information and requests for reagents may be directed to and will be fulfilled by the lead contact, Professor Gary Kleiger (gary.kleiger@unlv.edu).

#### Materials availability

All unique/stable reagents generated in this study are listed in the key resources table and are available from the lead contact with a completed Materials Transfer Agreement.

#### Data and code availability

- The atomic coordinates and electron microscopy maps have been deposited in the PDB with accession code PDB: 8Q7R (NEDD8-CRL2^FEM1C-Sil1^-UBE2R2~ubiquitin), PDB: 8R5H (NEDD8-CRL2^VHL-MZ1-BRD4 (346−460)^-UBE2R2~ubiquitin) and in the Electron Microscopy Data Bank with codes EMDB: EMD-18230 (NEDD8-CRL2^FEM1C-Sil1^-UBE2R2~ubiquitin; Krios), EMDB: EMD-18915 (NEDD8-CRL2^VHL-MZ1-BRD4 (346−460)^-UBE2R2~ubiquitin; Krios) and EMDB: EMD-18207 (NEDD8-CRL2^FEM1C-Sil1^-UBE2R2~ubiquitin; Glacios). Accession codes are listed in the key resources table. All mass spectrometry data have been uploaded to the ProteomeXchange Consortium via the PRIDE partner repository with the dataset identifier listed in the key resources table. Raw image data have been deposited at Mendeley with the DOI listed in the key resources table. All data are publicly available as of the date of publication.
- This paper does not report original code.
- Any additional information required to reanalyze the data reported in this paper is available from the lead contact upon request.

### EXPERIMENTAL MODEL AND STUDY PARTICIPANT DETAILS

#### Cell lines

##### High-five insect cells

Cells were grown in Sf-900 III SFM insect cell culture media at 27°C.

##### Sf9 insect cells

Cells were grown in ESF 921 insect cell culture media at 27°C.

##### HEK 293T Cells

Cells were grown in DMEM (High glucose, L-glutamine, Pyruvate) supplemented with 10% fetal bovine serum. The HEK 293T cells that were employed are female.

##### HEK 293T siRNA-mediated ARIH1 knockdown

Cells were grown in DMEM (High glucose, L-glutamine, Pyruvate) supplemented with 10% fetal bovine serum, GlutaMax, penicillin (100 units/ml), streptomycin (0.1 mg/ml), zeocin (100mg/ml) and blasticidin-HCl (15 mg/ml). The HEK 293T cells that were employed are female.

##### T47D, AU565, HCC1954, and EW16

Cells were grown in RPMI 1640 growth medium supplemented with 10% fetal bovine serum, penicillin, and streptomycin. T47D, AU565, and HCC1954 cells are female. EW16 cells are male.

##### Other cell lines

Cells were grown in DMEM (High glucose, L-glutamine, Pyruvate) supplemented with 10% fetal bovine serum.

Cells were maintained at 37°C in a humidified incubator with 5% CO_2_. Cells were routinely checked for mycoplasma contamination with LookOut mycoplasma PCR detection kit (Sigma).

NIH-3T3, MRC5, A-172, and HAP1 cells are male. MCF7, MDA-MB-231, MDA-MB-468, HT-29, SH-SY5Y and U2OS cells are female.

#### Organisms/strains

##### Escherichia coli *BL21 (DE3), Rosetta, and DH5α*

Cells were grown in Lysogeny broth (LB) at 18°C or 37°C.

### METHOD DETAILS

#### Constructs

Constructs that were generated for this study were prepared by standard molecular biology practices and DNA sequences were verified by Sanger sequencing. Mutants in this study were generated by standard site-directed mutagenesis protocols. For generation of pGEX His-RBX1-StrepII-GGGG-CUL2, the *MsyB* coding region (upstream from CUL2) was excised from pGEX His-RBX1-StrepII- CUL2, and a gene fragment for *MsyB* that contained a tetraglycine repeat at the C-terminus was ligated into the digested plasmid.

#### Peptides and protein purification

Peptides were either purchased from Vivitide (formerly New England Peptides) at > 95% purity and solubilized in water or synthesized in house in the Max Planck Institute für Biochemie. All single Lys peptide substrates had their N-termini acetylated (Ac). The hydroxylated Pro degron in Hif1α peptide is shown as hyP. The AZ-Dye peptides used for sortasing to CRLs were conjugated to the indicated fluorescent dyes using maleimide (Mal) chemistry. The Sil1 peptide substrate amino acid sequence was based on the clone 13 design from a previous study^106^ that had optimized the affinity of the peptide for FEM1C.

E1s: Human E1 was cloned into pLIB vectors and expressed in *Trichoplusia ni* High-Five insect cells via baculovirus infection. Human E1 was purified via GST affinity chromatography before being cleaved overnight with the TEV protease at 4°C, and subsequently further purified by anion exchange chromatography followed by gel filtration into storage buffer (30 mM Tris pH 7.5, 100 mM NaCl, 10% glycerol, and 1 mM DTT). Proteins were concentrated and drop-frozen in liquid nitrogen and stored at −80°C. Human APPBP1 and UBA3 were co-expressed in *Escherichia coli* BL21(DE3) cells. The APPBP1/UBA3 complex (NEDD8 E1) was purified through GST affinity chromatography, followed by overnight cleavage with the thrombin protease at 4°C and gel filtration into storage buffer (30 mM Tris pH 7.5, 100 mM NaCl, 10% glycerol, and 1 mM DTT). Proteins were concentrated and drop-frozen in liquid nitrogen and stored at −80°C.

E2s: Human UBE2R2 (and all mutant derivatives) were expressed in *E. coli* Rosetta(DE3) cells and purified through an N-terminal 6xHis-tag (Nickel-agarose; Qiagen), followed by cleavage overnight with TEV protease at 4°C, and gel filtration (SuperDex 200; Cytiva) into storage buffer (30 mM Tris pH 7.5, 100 mM NaCl, 10% glycerol, and 1 mM DTT). UBE2L3 (also known as UBCH7) was expressed in *E. coli* BL21 (DE3) cells and was purified by GST affinity chromatography, followed by overnight cleavage with thrombin protease at 4°C, and gel filtration chromatography (SuperDex 200) into storage buffer, followed by GST pass-back to eliminate GST impurities. Human UBE2M (also known as UBC12) was expressed in *E. coli* BL21(DE3) cells and purified using glutathione-S-transferase (GST) affinity chromatography, before being cleaved overnight with the thrombin protease at 4°C and subjected to cation-exchange chromatography followed by gel filtration into storage buffer (30 mM Tris pH 7.5, 100 mM NaCl, 10% glycerol, and 1 mM DTT). Proteins were concentrated and drop-frozen in liquid nitrogen and stored at −80°C. For details on UBE2R2 employed for binding studies, please see the section titled ‘Fluorescence-based binding assays’ below.

Ubiquitin and ubiquitin-like proteins: Wild-type human ubiquitin was purchased as a lyophilized powder (R&D Systems). Lys 48 Arg (K48R) human ubiquitin, a mutant where all Lys residues had been replaced with Arg (K0), and a ubiquitin that contain an aspartate residue at the C terminus (Asp77 ubiquitin) were expressed in *E. coli* BL21(DE3) cells by growing to an optical density of approximately 0.6 before inducing with 0.4 mM IPTG and harvesting after growing overnight. K48R, K0 and Asp77 ubiquitin were purified through their N-terminal 6xHis-tag, followed by gel filtration on a SuperDex 75 gel filtration column (Cytiva) that had been equilibrated in storage buffer (30 mM Tris pH 7.5, 100 mM NaCl, 10% glycerol, and 1 mM DTT). Human NEDD8 was expressed in *E. coli* BL21(DE3) and purified by virtue of its N-terminal GST tag, followed by cleavage with thrombin protease at 4°C, and gel filtration (SuperDex 75; Cytiva) into storage buffer.

CRL scaffolds: Human CUL2 and RBX1 were co-expressed in either *E. coli* BL21(DE3) (for details, please see the section titled ‘Fluorescence-based binding assays’ below) or *Trichoplusia ni* High-Five insect cells (all experiments employed this protein except the binding studies). Human CUL2 and RBX1 were co-expressed in *E. coli* and purified initially by virtue of its 6x-Histidine tag using Ni-NTA resin (Qiagen), followed by overnight digestion by TEV protease at 4°C. Following TEV cleavage, the protein was then diluted 1:5 in buffer (100 mM Tris-HCl pH 7.5 and 150 mM NaCl) and incubated with Strep-Tactin resin (IBA Lifesciences) for 2 hours, before being eluted with Strep-Tactin elution buffer (100 mM Tris-HCl pH 7.5, 150 mM NaCl, 2.5 mM desthiobiotin). Proteins were drop-frozen and stored at −80°C. For human CUL2 and RBX1 co-expressed in High-Five insect cells, baculoviruses for CUL2 and His-MBP-TEV-RBX1 (5-C) were used to transduce High-Five insect cells. Proteins were purified by Strep-Tactin affinity chromatography, followed by an overnight digestion with the TEV protease at 4°C. The complex was then further purified by anion exchange chromatography (HiTrap Q HP; Cytiva) followed by gel filtration on a SuperDex 200 column (Cytiva) into a buffer containing 25 mM HEPES pH 7.5, 150 mM NaCl, and 1 mM DTT.

CRL substrate receptor complexes: ELONGIN B/C-FEM1C complex: Human wild-type FEM1C (and all mutants) were co-expressed along with ELONGIN B and ELONGIN C in *E. coli* BL21(DE3) cells and grown at 37°C to an OD_600_ of 0.6 before being induced with 0.4 mM IPTG, and immediately followed by expression overnight at 16°C. ELONGIN B/C-FEM1C complex was purified through an N-terminal Glutathione-S-transferase (GST) tag on FEM1C (Glutathione Sepharose 4B, Cytiva), followed by cleavage with TEV protease at 4°C, and gel filtration chromatography (SuperDex 200) into storage buffer. ELONGIN B/C-VHL(54-C) complex: Expression constructs for human VHL (that lacked the first 53 residues) and ELONGIN B and ELONGIN C were co-transformed into *E. coli* BL21(DE3) cells with overnight expression as described above. The human ELONGIN B/C-VHL complex was purified through the N-terminal GST tag on VHL, followed by cleavage with thrombin protease at 4°C. The ELONGIN B/C-VHL complex was then subjected to ion exchange chromatography (HiTrap Q HP; Cytiva) followed by gel filtration (SuperDex 75; Cytiva) into storage buffer.

RBR E3s: Human ARIH1 was expressed in *E. coli* BL21 (DE3) cells and purified by virtue of its GST tag, followed by overnight cleavage with TEV protease at 4°C. ARIH1 was further purified using ion exchange chromatography (HiTrap Q HP; Cytiva), followed by gel filtration (SuperDex 200) into storage buffer.

Recombinant BRD4 proteins: All human BRD4 bacterial expression constructs encompassed the second bromodomain (BD2), where 333–460 was employed for mass-spec, and 346–460, K367R 346–460, and K368R 346–460 for *in vitro* ubiquitylation reactions. All BRD4 constructs were cloned using standard procedures and contained an N-terminal His-tag for purification by nickel affinity chromatography. Overnight TEV cleavage was followed by ion-exchange (HiTrap HP S; Cytiva) and size exclusion chromatography (SuperDex 75) into a buffer that contained 25 mM HEPES pH 7.5, 150 mM NaCl, and 1 mM DTT.

#### In vitro *neddylation*

Purified CUL2-RBX1 was neddylated as follows. Reactions were assembled in neddylation buffer (25 mM HEPES pH 7.5, 150 mM NaCl, 10 mM MgCl_2_, 5 mM CaCl_2_, and 2 mM ATP) by the sequential addition of 0.1 μM APPBP1 and UBA3, 1 μM UBE2M, 30 μM NEDD8, and 12 μM CUL2-RBX1 (all final concentrations are shown independent of the reaction volume). Reactions were initiated by the addition of CUL2-RBX1, incubated at room temperature for 20 minutes, followed by quenching with the addition of DTT (10 mM). Products were purified by gel filtration (SuperDex 200) into storage buffer.

#### *In vitro* reconstituted ubiquitylation assays

##### Peptide substrate and wild-type ubiquitin labeling

All labeled reactions were performed with 16 μM phosphate ^32^P-labeled ATP, 5 or 10 μM peptide, and protein kinase A in labeling buffer (New England Biolabs) and incubated at 32°C for 2 hours. Occasionally, peptides or wild-type ubiquitin were labeled at 50 μM upon such that additional non-radiolabeled ATP (50 μM final) was added to the reaction after 1 hour.

##### Steady-state ubiquitylation

Ubiquitylation reactions were performed under steady state conditions with respect to substrate (Hif1α peptide, Sil1 peptide, or BRD4^BD2^ protein neo-substrate (346–460) and various ubiquitin-carrying enzymes (UBE2R2, UBE2D3, UBE2G1, and ARIH1). A CRL mix tube was prepared with 0.5 μM neddylated CUL2-RBX1, 0.5 μM ELONGIN B/C-FEM1C or ELONGIN B/C-VHL, and 0.2 μM radio-labeled peptide substrate (Sil1 peptide or Hif1α peptide, respectively) diluted in reaction buffer (30 mM Tris-HCl pH 7.5, 100 μM NaCl, 5 mM MgCl_2_, 2 mM DTT, and 2 mM ATP). For reactions with BRD4^BD2^ (346–460), the CRL mix tube contained the same E3 concentration but had 0.5 μM radiolabeled BRD4^BD2^ neo-substrate (346–460) and 4 μM MZ1 PROTAC (MedChemExpress). In a separate reaction tube named E2 mix, 0.5 μM human E1 and 5 μM K0 ubiquitin were diluted in reaction buffer. 4 μL of the E2 mix was then aliquoted into separate tubes, before 1 μL of 5 μM ubiquitin-carrying enzyme (UBE2R2, UBE2D3, UBE2G1 or ARIH1) was added. For reactions involving UBE2R2, K48R ubiquitin was used in the E2 mix instead of K0 ubiquitin. For mixtures involving ARIH1, 2 μM UBE2L3 was included. These tubes (including the CRL mix tube) were incubated for 15 minutes at room temperature, except for tubes containing ARIH1 where no incubation period was involved. Reactions were initiated by mixing an equal volume of the CRL mix to each aliquoted tube. Reactions were quenched with 2X SDS-PAGE loading buffer (100 mM Tris-HCl pH 6.8, 20% glycerol, 30 mM EDTA, 4% SDS and 4% β-mercaptoethanol) after a 10 second incubation period and were resolved on Tris-Glycine 18% SDS-PAGE gels. Autoradiography was performed using an Amersham Typhoon 5 imager and quantified using ImageQuant software (Cytiva). The fraction of ubiquitylated product was calculated by the signal of products (defined as a substrate peptide that had been conjugated with one or more ubiquitins) divided by the total signal (products and substrate).

##### Estimation of the K_m_ of a ubiquitin-carrying enzyme for CRL

Reactions were assembled in two separate mixtures with stock proteins being diluted with reaction buffer. The assembly of the CRL-substrate complex was performed by the sequential addition of neddylated CUL2-RBX1, followed by the substrate receptor complex, and finally ^32^P-labeled peptide substrate (tube 1). While the tube 1 components were incubating at room temperature, the tube 2 solution was assembled through the addition of E1 and ubiquitin (K48R or K0 depending on the ubiquitin-carrying enzyme component; please see the tables below). Following a one-minute incubation period, the tube 2 solution was evenly aliquoted into 9 individual Eppendorf tubes. Next, dilutions of UBE2R2 or ARIH1 that had previously been serially diluted by 2-fold were added to the Eppendorf tubes to initiate ubiquitin-carrying enzyme charging with ubiquitin. Please note that for ARIH1-based ubiquitylation reactions, the required ubiquitin-carrying enzyme UBE2L3 was also included in tube 2 and incubated for 2 minutes prior to aliquoting into the Eppendorf tubes. For all reactions containing ARIH1,^107^ UBE2L3 protein levels were kept constant in an amount that was in excess of the highest ARIH1 concentration (the final concentrations of all protein reagents are reported in the tables below). For UBE2R2 reactions, the aliquots were pre-incubated for 15 minutes to allow for complete charging of the ubiquitin-carrying enzyme with ubiquitin. To establish single encounter conditions between radiolabeled substrate and the CRL complex, excess unlabeled competitor peptide substrate was added to the solutions prior to initiation of the reaction. For ubiquitylation reactions containing ARIH1, the competitor peptide was added immediately after the addition of ARIH1. For reactions involving Sil1, the addition of competitor peptide was not necessary due to the slow off-rate of Sil1 from the CRL relative to the duration of the reaction prior to quenching. Each ubiquitylation reaction was initiated by the addition of an equal volume of solution from tube 1 to the tubes containing charged ubiquitin-carrying enzymes and then quenched in 2X SDS-PAGE loading buffer after a 10 second incubation period. Substrate and products were resolved on Tris-Glycine 18% SDS-PAGE gels. Autoradiography was performed using an Amersham Typhoon 5 imager and quantification by ImageQuant software. The fraction of substrate ubiquitylated was calculated as the signal of products (defined as a substrate that had been modified by at least one ubiquitin) and divided by the total signal (products and substrate). The fraction ubiquitylated values were plotted as a function of the ubiquitin-carrying enzyme concentrations on a graph and the data were fit to the Michaelis-Menten model using nonlinear regression (GraphPad Prism v10 software). Final protein concentrations can be found in Table S3.

##### Pre-steady-state single-encounter reactions

Reactions were assembled in two separate mixtures as described in the previous section and according to the concentrations in the table below. After the appropriate incubation periods had passed, solutions from tubes 1 and 2 were loaded into separate loops on a KinTek RQF-3 Quench-Flow instrument. Reactions were initiated by bringing the two mixes together in drive buffer (30 mM Tris-HCl pH 7.5 and 100 mM NaCl) and then quenched at various time points in reducing 2X SDS-PAGE loading buffer. Substrate and products at the various time points were resolved on Tris-Glycine 18% SDS-PAGE gels. Autoradiography was performed using an Amersham Typhoon 5 imager and quantification performed in ImageQuant software (Cytiva). The signal was calculated as the depletion of unmodified peptide substrate, calculated by taking the signal of unmodified peptide substrate (S0) and dividing it by the total signal in the lane. The rates of ubiquitin transfer were determined by fitting to analytical closed-form solutions^60^ in Mathematica (v13.1). For estimation of the chain forming activity of UBE2R2, pre-steady-state ubiquitylation reactions with neddylated CRL2^VHL^ and Hif1α peptide were performed with identical conditions as described in the table below, but with wild-type instead of K48R ubiquitin. Reactions were prepared and initiated as described above. The signal corresponding to unmodified substrate (S0) and ubiquitin-primed Hif1α peptide substrate (S1; see Figure S1B) were estimated as fractions of the total signal in the lane. The rates of substrate priming (i.e. S0 conversion to S1) and poly-ubiquitin chain extension (i.e. S1 to S2) were estimated by fitting the data to their respective analytical closed-form solutions^60^ in Mathematica (v13.1). Final protein concentrations can be found in Table S3.

##### Reactions with PROTACs

Reactions were assembled as described above (except without the addition of unlabeled competitor peptide) for the estimation of *K_m_* and *k_obs_* with the exception that PROTACs were added to tube 1 following formation of the CRL complex. PROTACs were purchased as lyophilized powders (MedChemExpress) and solubilized in 100% DMSO at 1 mM. Final DMSO concentrations in the ubiquitylation reactions were either 1% (*K_m_*) or below 1% (*k_obs_*). Final protein concentrations can be found in Table S3.

##### Single-encounter ubiquitylation control reactions

Ubiquitylation reactions were performed under single-encounter conditions (radiolabeled substrate for E3) to assess the specificity of various UBE2R2 or FEM1C mutants affecting Sil1 substrate priming. Reactions involving UBE2R2 mutants were performed as follows: a tube was assembled in reaction buffer containing 0.5 μM neddylated CUL2-RBX1, 0.5 μM ELONGIN B/C-VHL, and 0.2 μM radiolabeled Hif1α peptide substrate. Another separate tube was also prepared in reaction buffer, containing 0.5 μM E1, 5 μM K48R ubiquitin, 1 μM E88R or V146A UBE2R2, and 20 μM unlabeled Hif1α peptide substrate. These two tubes were then incubated at room temperature for 15 minutes. Reactions were initiated by mixing equal volumes of both tubes together and quenching the reaction with 2X SDS-PAGE buffer at various timepoints. Substrates and products were resolved on 18% Tris-Glycine SDS-PAGE gels. Following autoradiography on an Amersham Typhoon 5 imager, quantification of substrate and product was performed using ImageQuant software (Cytiva). Reactions involving FEM1C mutants were performed as follows: a tube was assembled in reaction buffer, containing 0.5 μM neddylated CUL2-RBX1, 0.5 μM ELONGIN B/C-FEM1C (mutant or wild-type), and 0.2 μM radiolabeled Sil1 peptide substrate. In a separate tube, 0.5 μM E1, 5 μM K0 ubiquitin, and 2.5 μM UBE2L3 were combined and incubated at room temperature for 2 minutes prior to the addition of 250 nM ARIH1. Next, equal volumes of both tubes were then mixed to initiate the reaction at room temperature and then quenched with 2X SDS-PAGE buffer at the various timepoints. Substrates and products were resolved and quantified as described above.

##### Di-ubiquitin synthesis with wild-type and E88R UBE2R2

Di-ubiquitin synthesis reactions were assembled as follows: a CRL mix tube containing 0.5 μM neddylated CUL2-RBX1 and 50 μM Asp77 ubiquitin diluted in reaction buffer was prepared. Note that Asp77 ubiquitin cannot be conjugated to an E2 owing to blocking of its GlyGly C terminus and thus can only form chains with E2-conjugated donor ubiquitin. For reactions involving ELONGIN B/C-FEM1C, the CRL mix tube instead contained 0.5 μM neddylated CUL2-RBX1, 0.5 μM ELONGIN B/C-FEM1C, and 2.5 μM Asp77 ubiquitin. Another tube contained 0.5 μM human E1, 4 μM ^32^P-labelled K48R donor ubiquitin, and 2 μM of either wild-type or E88R UBE2R2 diluted in reaction buffer. The radiolabeled donor ubiquitin contained a K48R mutation owing to UBE2R2’s preference to forge poly-ubiquitin chains with Lys48 specificity. The combination of K48R donor ubiquitin and Asp77 ubiquitin ensures the formation of a single di-ubiquitin product. These two solutions were incubated at room temperature for 15 minutes. Reactions were initiated by mixing equal volumes of both mixtures together before being quenched in 2X SDS-PAGE buffer after 10 seconds. Donor ubiquitin substrate and di-ubiquitin product were resolved on 18% Tris-Glycine gels by SDS-PAGE, followed by autoradiography (Typhoon 5 image scanner; Cytiva) and quantification (ImageQuant). The fraction of di-ubiquitin product was determined by dividing the signal of di-ubiquitin by the total signal in the lane. See Figure 7D for a schematic of the assay.

#### Generation of *UBE2G1* knockout cells

Wild-type HEK 293T cells were plated and transfected with a PX330 vector containing a small guide sequence targeting exon 1 of *UBE2G1* as well as a single stranded DNA oligo that contained several consecutive stop codons to promote homologous recombination at the cut site. Cells were then diluted to achieve single colonies upon their application onto 96-well plates. Colonies were first identified by visualization under a light microscope and expanded until testing for incorporation of the DNA oligo by PCR.

#### TMT-10plex global proteome profiling

HEK 293T control and *UBE2R1/UBE2R2* DKO cell lines were employed for global proteome profiling^37^ (control cell line clone identifiers #1, G3 and D5; *UBE2R1/UBE2R2* DKO clone identifiers B3, E4, and A10). Cells were grown at 37°C in tissue media that contained DMEM (4.5 g/L glucose) supplemented with 10% fetal bovine serum, 4 mM L-Glutamine, 100 units/mL Penicillin, 100 mg/mL Streptomycin, and 10 mg/mL Ciprofloxacin in tissue culture incubator with 5% carbon dioxide, harvested via trypsination, washed in PBS, pelleted via centrifugation and flash frozen to later be lysed in a denaturing buffer (8 M urea, 20 mM HEPES pH 8.0, Roche cOmplete protease inhibitors) via sonication using a Qsonica Q125 (Converter Model CL-18, microtip 1/8” #422-A) for 20 seconds (1 second ON/1 second OFF) at 55% amplitude. Lysates were cleared via centrifugation at 18,000 x g at 4°C for 10 minutes and protein concentrations of the soluble fraction were determined using a BSA assay. Subsequently, 500 μg total protein lysate in a volume of 100 μL was reduced (5 mM DTT, for 45 minutes at 37°C) and alkylated (15 mM Iodoacetamide, for 15 minutes at 23°C, followed by quenching via increasing DTT to 10 mM) under constant shaking at 850 rpm in a ThermoMixer. Proteins were then precipitated via a chloroform-methanol precipitation through gentle stepwise mixing of 100 μL lysate with 400 μL methanol, then with 100 μL chloroform, and finally with 300 μL H_2_O. Mixtures were spun at 14,000 x g for 2 minutes at room temperature (RT), top and bottom fluid layers aspirated, and the protein precipitate washed twice in methanol. Precipitates were air-dried and resuspended in fresh 8 M Urea (in 50 mM HEPES pH 8.0), diluted to 4 M Urea in 50 mM HEPES pH 8.0, and lysyl-endopeptidase C (Lys-C, Wako) was added at 1 μg per 100 μg total protein before incubation at 37°C for 4 hours under constant shaking. Afterwards, samples were diluted to 1.5 M Urea in 50 mM HEPES pH 8.0 and sequencing grade trypsin (Promega) was added at 1 μg per 50 μg total protein followed by an overnight incubation at 23°C and 850 rpm. Resultant peptides were acidified via addition of 50% formic acid solution to a final concentration of ~5% formic acid and desalted via Sep-Pak C18 (50 mg cartridge) solid phase extraction (Waters). Eluted peptides were flash-frozen and dried to be resuspended in 200 mM HEPES pH 8.0 at a concentration of ~1 mg/mL and 100 μg of total protein per sample was used for TMT labeling reactions: 100 μL at 1 mg/mL protein concentration were mixed with 20 μL of anhydrous acetonitrile before 20 μL of freshly reconstituted TMT-10plex label (Thermo Scientific) was added followed by incubation at room temperature for 1 hour. Labeling reactions were temporarily halted at −80°C, while label and ratio checks were performed via small-scale MS analysis, and ultimately quenched via addition of 20 μL of 5% hydroxylamine while incubating at room temperature for 15 minutes. Equal amounts of all samples were pooled after a TMT incorporation rate of >98% was confirmed and the combined sample dried and re-suspended in 500 μL 0.1% trifluoroacetic acid. Reconstituted peptides were desalted using a 200 mg Sep-Pak C18 cartridge and subjected to fractionation by basic pH Reverse-Phase liquid chromatography on an Agilent 1260 Infinity II LC system HPLC outfitted with an Agilent ZOBRAX 300Extend-C18 column (Rapid Resolution, 4.6×150mm, 3.5 Micron) and an Agilent 1260 Infinity Bio-inert analytical scale fraction collector. 96 fractions were collected,^108^ 48 of which were combined into 12 final fractions which were desalted via the STAGE-TIP procedure^109^ and subjected to MS analysis.

##### Mass spectrometry analysis of TMT samples

Dried TMT-labeled samples from global proteome profiling experiments were reconstituted in Buffer A (2% acetonitrile, 0.1% formic acid, in 98% H_2_O) for analysis via liquid chromatography-tandem mass spectrometry (LC-MS) using a Dionex Ultimate 3000 RSLCnano system (Thermo Fisher Scientific, Inc) coupled with an Orbitrap Fusion Lumos Tribrid MS (Thermo Fisher Scientific, Inc). Peptides were resolved on an IonOpticks (Aurora Series) column by the following gradient with Buffer A (2% acetonitrile, 0.1% formic acid, 98% H_2_O) and Buffer B (98% acetonitrile, 0.1% formic acid, 2% H_2_O): 0 minutes-4.9 minutes 2% Buffer B at a flow rate 400 nL/min, 4.9 minutes-10 minutes 2% Buffer B at 300 nL/min, 10 minutes-145 minutes from 2% to 30% Buffer B at 300 nL/min, 145 minutes-160 minutes from 30% to 50% Buffer B at 300 nL/min, 160 minutes-163 minutes from 50% to 90% Buffer B at 300 nL/min, 163 minutes-169.9 minutes 90% Buffer B at 300 nL/min, 169.9 minutes-185 minutes 2% Buffer B at 400 nL/min. MS analysis was performed using a Multi-Notch MS3-based TMT method. Each duty cycle included an MS1 scan in the Orbitrap at 120,000 resolution across 350–1350 m/z range with automatic gain control (AGC) target of 1.0e6, 50 ms maximum injection time. Data dependent ion trap MS2 scans were performed on the top 10 peptides with CID activation at a collision energy of 35%, at 0.5 m/z isolation window in the quadrupole, turbo scan rate, 2.0e4 AGC target, 100 ms maximum injection time. Eight MS2 fragment ions were selected for Orbitrap SPS-MS3 scans with isolation widths of 1.2 m/z using isolation waveforms with multiple frequency notches. These ions were fragmented during MS3 by high energy collision-induced dissociation (HCD) at a collision energy of 55% and analyzed by Orbitrap at 50,000 resolution, 2.5e5 AGC target, 150 ms maximum injection time.

##### Mass spectrometry TMT data analysis

MS data were searched using Mascot (Matrix Sciences, London, UK) against a target-decoy database that included UniProt *Homo sapiens* protein sequences (August 2017 version), known contaminants, and the reversed protein sequences. Search parameters were 50 ppm precursor ion mass tolerance and 0.8 Da fragment for ion tolerance, permitting up to 2 missed cleavages. Carbamidomethylation on cysteines (+57.0215 Da) and TMT modification on the N terminus and lysines (+229.1629 Da) were also considered. Variable modifications included methionine oxidation (+15.9949 Da) and TMT modification on tyrosine (+229.1629 Da). Peptide level and protein level search results were filtered to <1% FDR and <2% FDR, respectively. The following parameters were used for the analysis: TMP (Tukey’s median polish) as the peptide to protein summarization method, global median normalization (Equalize Medians parameter), moderated t-test for hypothesis testing, and with Benjamini-Hochberg adjustment method for multiple comparisons.

#### Cellular degradation in *UBE2R1/UBE2R2* DKO cells

##### Cloning

Coding sequences for UBE2R1 and UBE2R2 expression were cloned into a pHR’CMV vector obtained from Addgene (plasmid #23135). Plasmids were used for generating stable rescue lines.

##### Cell culture conditions

Cell lines were grown in Dulbecco’s modified Eagle’s medium (DMEM) supplemented with 10% fetal bovine serum (Gibco) at 37°C in a humidified incubator with 5% CO_2_. Cells were periodically tested for mycoplasma with LookOut Mycoplasma PCR Detection Kit (Sigma).

##### Generation of stable cell lines

Lentivirus for UBE2R1 or UBE2R2 was generated by co-transfection with packaging (psPAX2) and enveloping (pMD.2G) plasmids into HEK 293T cells using Lipofectamine 3000. 48 hours post-transfection viral particles were harvested, and viral titer determined using Lenti-X GoStix Plus (Takara). Control and *UBE2R1/UBE2R2* double knockout (DKO), MDA-MB-468 or MRC-5 cells were infected at a MOI of 0.5 in complete media supplemented with 6 μg/ml polybrene. 48 hours post infection, cells were harvested and plated in complete media containing 100 μg/ml (HEK 293T and MRC-5) or 300 μg/ml Hygromycin (MDA-MB-468). After 14 days of selection, cells were harvested and screened for expression of UBE2R1 or UBE2R2 by western blotting.

##### Cell biology experiments

Control, *UBE2R1/UBE2R2* DKO, and rescue cell lines were plated onto 6-well plates (800,000 cells/well). Approximately 18 hours later, cells were treated with either DMSO or PROTACs including ARV-771, dBET1, or MZ1 (500 nM) diluted in complete media for 6 hours. MDA-MB-468 cells were treated identically except cells were exposed to PROTACs for 2 hours, while MRC-5 cells were treated for 1 hour.

##### Western blot analysis

Total cell lysates were prepared by harvesting cell pellets that were first washed and resuspended in lysis buffer (6 M Urea, 50 mM Tris-HCl pH 7.6, 150 mM NaCl, 0.5% NP-40, 0.1% SDS) supplemented with Halt^™^ protease and phosphatase inhibitor cocktail (Thermo Fisher) and Universal nuclease (Pierce). Cells were periodically pipetted on ice for 20 minutes. Lysates were cleared by centrifugation at 14,000 rpm for 10 minutes. Total protein was quantified with BCA protein assay (Pierce). Equivalent amounts of lysate (typically 25 μg) were separated on 4–12% Bis-Tris gels (Invitrogen) and transferred onto polyvinylidene difluoride membranes. Membranes were blocked in 5% non-fat dry milk powder (BIO-RAD) in Tris-buffered saline with 0.05% Tween for 1 hour at room temperature. Membranes were then incubated with primary antibodies overnight at 4°C, washed three times with TBST, incubated with secondary antibody at room temperature for 1 hour, and washed again before incubation with SuperSignal West Pico substrate (Thermo Fisher Scientific). Blots were imaged using an ImageQuant LAS4000 imaging system. Band intensity was quantified with ImageQuant. Raw BRD2, BRD3, and BRD4 signals were normalized according to the GAPDH protein level for each lane. The following antibodies were used at 1:1000 dilution: UBE2R1 (Abcam ab204515), UBE2R2 (Santa Cruz sc134628), BRD2 (CST 5848), BRD3 (Santa Cruz sc-81202), BRD4 (CST 13440), ZER1 (Invitrogen PA5–21807), and KLHDC2 (Prestige HPA000628). The following antibodies were used at 1:6000 dilution: GAPDH (Santa Cruz sc-32233).

#### Substrate degradation with ARIH1-targeting siRNA

##### Cell lines and cell culture

Flp-In T-Rex Human embryonic kidney 293 (HEK 293) cells (Thermo Fisher Scientific) were cultured in Dulbecco’s modified Eagle medium (DMEM; GIBCO) supplemented with 10% (v/v) fetal bovine serum (FBS) (GIBCO), GlutaMax (GIBCO), penicillin (100 units/ml), streptomycin (0.1 mg/ml) (Gibco), zeocin (100 mg/ml), and blasticidinS-HCl (15 mg/ml) at 37°C in a 5% CO_2_ humidified incubator. Flp-In T-Rex HEK 293 cells with stably integrated siRNA resistant N-terminally GFP-tagged ARIH1 (GFP-ARIH1^res^) were generated using the Flip-In system following the manufacture’s protocol (Invitrogen).

##### PROTAC treatment, lysates, and immunoblot analysis

Flp-In T-REx HEK 293+GFP-ARIH1 cells were transfected with either siNonTargeting (siNT) or siARIH1 (5′ CGAGAUAUUU CCCAAGAUU) using RNA-Max lipofectamine for 96 hours. Where indicated, expression of GFP-ARIH1^res^ was induced with 0.5 μg/ml tetracycline. The cells were mock-treated (DMSO) or treated with the PROTACs ARV-771, dBET1, and MZ1 at a final concentration of 500 nM for 6 hours. Cells were rinsed twice with ice-cold 1X PBS and lysed on ice in lysis buffer (25 mM Tris-HCl pH 7.4, 150 mM NaCl, 1 mM EDTA, 1% NP-40, 5% glycerol) and supplemented with Halt^™^ protease and phosphatase inhibitor mix (Thermo Fisher). Cell lysates were clarified by centrifugation and subjected to immunoblot analysis using the following antibodies: BRD4 (CST: 13440), ARIH1 (homemade^29^), and GAPDH (Abcam: ab9484).

#### Cellular UBE2R1 and UBE2R2 protein levels

##### Cell culture conditions

Cell lines (Excel File S2) were grown in Dulbecco’s modified Eagle’s medium (DMEM) supplemented with 10% fetal bovine serum (Atlanta Biologicals) and penicillin and streptomycin (Invitrogen) at 37°C in a humidified incubator with 5% CO_2_. Other cell lines, including T47D, AU565, HCC1954, and EW16 were grown in RPMI 1640 growth medium supplemented with 10% fetal bovine serum and penicillin and streptomycin using the same incubator conditions.

##### Western blot analysis

For preparing total cell lysates, cells were washed once with cold PBS, and lysed via scraping in RIPA lysis buffer (50 mM Tris-HCl pH 8.0, 150 mM NaCl, 0.1% Triton, 0.5% sodium deoxycholate, 0.1% SDS) supplemented with protease and phosphatase inhibitor cocktail (Halt PPI; Thermo Fisher Scientific). Lysates were incubated on ice for 30 minutes and then centrifuged at 14,000 rpm for 20 minutes at 4°C. Total protein was quantified using the Bradford assay (BIO-RAD). Sample concentrations were first equalized, and Laemmli sample buffer (BIO-RAD) was added to a final concentration of 1X and boiled for 7 minutes at 95°C. 15–20 μg of protein were loaded for each sample, and proteins were separated by SDS-PAGE on 4–20% poly-acrylamide gels, then transferred to polyvinylidene difluoride membranes (Immobilon-P PVDF; Sigma) using the Trans-Blot Turbo Transfer System (BIO-RAD). Membranes were blocked in 5% non-fat dry milk powder (BIO-RAD) in Tris-buffered saline with 10% Tween for 1 hour at room temperature. Membranes were then incubated with primary antibodies overnight at 4°C, washed three times with TBST, incubated with secondary antibody at room temperature for 1 hour, and washed again before incubation with SuperSignal West Dura substrate (Thermo Fisher Scientific). Blots were imaged using the LI-COR Fc Odyssey imaging system. The antibodies used were specific for UBE2R1 (1:2,000), UBE2R2 (1:2,000), and β-actin (1:10,000; Millipore Sigma). The secondary antibodies used were rabbit IgG HRP, and mouse IgG HRP (1:10,000; both from BIO-RAD). Quantification of band intensity was calculated using the ImageStudio analysis platform (LI-COR Biosciences).

#### *In vitro* PROTAC-dependent ubiquitylation assays

##### Ubiquitylation assay for LC-MS/MS

Ubiquitylation assays were performed for liquid chromatograph mass spectrometry (LC-MS/MS). 20 μM UBE2R2 was charged with 22.5 μM fluorescently labeled K48R ubiquitin by incubation with 0.3 μM UBA1 in a buffer containing 50 mM HEPES pH 7.5, 100 mM NaCl, 2.5 mM MgCl_2_, 1.5 mM ATP, and 0.05 mg/ml BSA (final concentrations for the charging reaction are shown). Reactions were quenched after 30 minutes by adding an EDTA solution to a final concentration of 30 mM. Subsequently, UBE2R2~ubiquitin K48R (approximately 0.5 μM final) was incubated with 1 μM ELONGIN B/C-VHL complex, 1 μM neddylated CUL2-RBX1, 5 μM MZ1 and 4 μM BRD4 (333–460) for 10 minutes in a buffer containing 25 mM HEPES pH 7.5 and 150 mM NaCl. The ubiquitylation reactions were then quenched with 10 mM DTT and submitted for mass spectrometry analysis.

##### LC-MS/MS sample preparation and data acquisition

BRD4 (333–460) samples were left untreated or enzymatically digested with 1 μM LBpro protease (purified in-house^110^) for 8 hours. Hereafter, samples were five-fold diluted in 50 mM ammonium bicarbonate, pH 8.0 and boiled for 5 minutes at 95°C. For protein reduction and alkylation, tris(2-carboxyethyl)phosphine (TCEP) and chloroacetamide were added to a final concentration of 10 mM and 40 mM, respectively, followed by 5 minutes of incubation at 45°C. Samples that had not been exposed to LBpro was cleaved with trypsin (1:20 w/w; Sigma-Aldrich) at 37°C overnight. Similarly, LBpro digested samples were equally split and enzymatically digested using either GluC (1:20 w/w; BioLab) or AspN (1:20 w/w; Promega) at 37°C overnight. The enzymatic activity was first quenched, followed by the loading of peptides onto homemade SDB-RPS StageTips. Briefly, after peptide loading, peptides were washed twice with 200 μL 0.2% trifluoroacetic acid and eluted with 60 μL ammonium hydroxide in 80% acetonitrile. Eluted peptides were dried in a Concentrator Plus (Eppendorf) and resuspended in Buffer A (2% acetonitrile/0.1% trifluoroacetic acid) for LC/MS-MS analysis.

The peptide concentration was determined optically by measuring the absorbance at 280 nm (NanoDrop 2000; Thermo Scientific).100 ng of peptide material was loaded onto a 50 cm reversed phase column (75 μm inner diameter, packed in-house with ReproSil-Pur C18-AQ 1.9 μm resin (Dr. Maisch GmbH)). Peptides were separated with a binary buffer gradient consisting of Buffer A (0.1% formic acid) and B (0.1% formic acid in acetonitrile) at a constant column temperature of 60°C. The LC-MS setup consisted of an EASY-nLC 1200 system (Thermo Fisher Scientific), which was directly coupled online with the mass spectrometer (Exploris 480; Thermo Fisher Scientific) via a nano-electrospray source. Peptides were stepwise eluted with a gradient starting at 3% Buffer B and stepwise increased to 8% in 8 minutes, 36% in 32 minutes, 45% in 4 minutes and 95% in 4 minutes at a constant flow rate of 300 nL/min. The mass spectrometer was operated in Top12 data-dependent mode (DDA) with a full scan range of 250–1350 m/z at 60,000 resolution with an automatic gain control (AGC) target of 300% and a maximum fill time of 20 milliseconds. Precursor ions were isolated at a width of 1.4 m/z and fragmented by higher-energy collisional dissociation (HCD) with a normalized collision energy (NCE) of 28%. Fragment scans were performed at a resolution of 30,000, an 1000% AGC and a maximum injection time of 110 milliseconds. Dynamic exclusion was enabled and set to 30 seconds.

For ubiquitylation site mapping, raw MS data were searched against the UniProt Human FASTA (downloaded 19^th^ of November 2020) and a FASTA file containing the BRD4 (333–460) sequence using MaxQuant (version 1.6.7.0). Cysteine carbamidomethylation was set as fixed and N-terminal acetylation, methionine oxidation and lysine diGly as variable modifications. The minimum peptide length was set to seven amino acids and a maximum of two missed cleavages were allowed for peptide identification, while permitting a maximum of five modifications per peptide. Enzyme specificity was set to trypsin, AspN and GluC for digestion approaches using trypsin, AspN and GluC, respectively.

##### BRD4 “pulse-chase” ubiquitylation assay

20 μM UBE2R2 (final concentration) was charged with 22.5 μM fluorescently labeled K48R ubiquitin in the presence of 0.3 μM UBA1 in a buffer containing 50 mM HEPES pH 7.5, 100 mM NaCl, 2.5 mM MgCl_2_, 1.5 mM ATP, and 0.05 mg/ml BSA. Reactions were stopped after a 30-minute incubation by adding an EDTA solution (30 mM final), followed by adding approximately 0.5 μM UBE2R2~ubiquitin to a fresh tube containing 0.5 μM ELONGIN B/C-VHL complex, 0.5 μM neddylated CUL2-RBX1, 5 μM MZ1 and 2 μM BRD4 (346–460), K367R BRD4 (346–460), or K368R BRD4 (346–460) in a buffer that contained 25 mM HEPES pH 7.5 and 150 mM NaCl buffer. Ubiquitylation reactions were quenched after a 5-minute incubation period with non-reducing SDS-PAGE sample buffer and substrate and products were separated by SDS-PAGE. Gels were scanned using an Amersham Typhoon system (GE Healthcare) and bands intensities were quantified with ImageQuantTL (GE Healthcare) software.

#### Fluorescence-based binding assays

##### Constructs

Human CUL2 and RBX1 were co-expressed in bacteria as described in the protein purification section and with a N-terminal tetraglycine motif (GGGG) added to the CUL2 N terminus for sortasing (^GGGG^CUL2-RBX1). Briefly, a vector that contained the gene for full-length CUL2 was treated with restriction enzymes NcoI/NheI to release the *MsyB* gene that had been included to improve the solubility of the complex in bacteria.^94^ A gene fragment was synthesized (IDT) that coded for *MsyB* with a C-terminal TEV protease consensus site (ENLYFQG) that upon cleavage reveals a tetraglycine repeat. The synthetic gene was used to replace the original *MsyB* gene upstream of the CUL2 N terminus. Similarly, human UBE2R2 was also cloned to contain an N-terminal tetraglycine motif by modifying a previously existing UBE2R2 construct in pET11b that contained an N-terminal histidine tag followed by a TEV protease site. Briefly, gene synthesis (IDT) was employed to generate a UBE2R2 construct as described above and with the active site Cys residue replaced by a Lys to facilitate the stable incorporation of a donor ubiquitin (^GGGG^UBE2R2^C93K^). A wild-type version of the same construct (^GGGG^UBE2R2^wild-type^) was cloned to enable assessment of the effect of fluorescent labeling on UBE2R2 ubiquitylation activity.

##### Expression and purification of ^GGGG^CUL2-RBX1

^GGGG^CUL2-RBX1 was expressed by transformation of the construct into BL21(DE3) cells and grown at 37°C until the culture had achieved an OD_600_ of 0.6, followed by the induction of protein expression through the addition of 0.4 mM IPTG and incubation overnight at 16°C. Cells were harvested by centrifugation at 5000 x g and pellets were frozen in liquid nitrogen and stored at −80°C for future use. To prepare lysates, the bacterial cells were suspended in lysis buffer (30 mM Tris-HCl pH 7.5, 200 mM NaCl, 10% glycerol, 5 mM DTT, and protease inhibitor cocktail) and sonicated. The MsyB-TEV-^GGGG^CUL2-RBX1 protein was purified by batch purification using Ni-NTA resin (Qiagen) before overnight incubation with TEV protease at 4°C. Cleaved ^GGGG^CUL2-RBX1 were then subjected to further batch purification using Strep-Tactin resin (IBA Lifesciences), washed with Strep-Tactin wash buffer (100 mM Tris pH 8.0, 150 mM NaCl) and eluted in a buffer containing 100 mM Tris pH 8.0, 150 mM NaCl, and 2.5 mM desthiobiotin. The resulting eluate was then concentrated using a 10 kDa centrifugal filter (Millipore-Sigma) and drop frozen in liquid nitrogen prior to storage at −80°C. The final protein complex purity can be found within the Mendeley data file (please see the key resources table).

##### Expression and purification of ^GGGG^UBE2R2^C93K^

^GGGG^UBE2R2^C93K^ was expressed by transformation of the construct into Rosetta(DE3) bacterial cells and grown at 37°C to an OD_600_ of 0.6, followed by the induction of expression by adding 0.4 mM IPTG and incubation for 3 hours at 30°C. Cells were harvested by centrifugation at 5,000 x g and pellets frozen in liquid nitrogen prior to long-term storage at −80°C. Lysates were prepared by suspending the cell pellets in lysis buffer (30 mM Tris-HCl pH 7.5, 200 mM NaCl, 10% glycerol, 5 mM DTT, and protease inhibitor cocktail) and sonication. The ^GGGG^UBE2R2 protein was isolated from bacterial proteins by batch purification using Ni-NTA resin (Qiagen) followed by overnight digestion by incubation with TEV protease. The next day, samples were subjected to anion exchange chromatography (HiTrap Q; Cytiva). Fractions containing ^GGGG^UBE2R2^C93K^ were first concentrated using a 10 kDa centrifugal filter (Millipore-Sigma) and further purified by injection onto a SuperDex 75 (Cytiva) column that had been equilibrated in storage buffer (30 mM Tris pH 7.5, 100 mM NaCl, 10% glycerol, 1 mM DTT) and controlled by a BioRad NGC system. The final protein purity can be found within the Mendeley data file (please see the key resources table).

##### In vitro *neddylation and generation of NEDD8-^Fluor568^CUL2-RBX1*

The purified ^GGGG^CUL2-RBX1 protein complex was the substrate for a neddylation reaction performed at room temperature for 20 minutes in reaction buffer (25 mM HEPES pH 7.5, 150 mM NaCl, 10 mM MgCl_2_, 5 mM CaCl_2_ and 2 mM ATP) and in the presence of 0.1 μM NEDD8 E1, 1 μM UBE2M, 12 μM ^GGGG^CUL2-RBX1, and 30 μM NEDD8. Next, a synthetic peptide that contained a fluorescent dye at the N terminus and a consensus sortasing site at the C terminus (Nterm-[C-568Mal]GSGGLPETGG-Cterm; Vivitide) was covalently fused to the N terminus of ^GGGG^CUL2-RBX1 in the presence of 10 μM sortase and 100 μM peptide. The reaction was incubated for 2 hours and 30 minutes at room temperature and quenched with 10 mM DTT. The reaction contents were then passed through a 0.22 μm filter before gel filtration over a SuperDex 75 column that had been equilibrated in storage buffer. Proteins were drop-frozen in liquid nitrogen and stored at −80°C. The final protein complex purity can be found within the Mendeley data file (please see the key resources table).

Since the fluorescent dye that had maximal absorption at 568 nm nevertheless contributed to the protein light absorption at 280 nm, the following protocol was developed to assess the final protein concentration. A construct had been developed where the RBX1 subunit of CUL2-RBX1 contained the N-terminal tetraglycine sortasing motif and was employed to produce fluorescently labeled CUL2-RBX1. Here sortasing of the fluorescent peptide to the RBX1 N terminus resulted in a quantifiable change in electrophoretic mobility and led to our estimating that nearly all of the RBX1 had been modified by peptide. Next, a 2-fold titration series was performed using both NEDD8-^Fluor568^CUL2-RBX1 and NEDD8-CUL2- ^Fluor568^RBX1 and was scanned on a Typhoon 5 fluorescence imager. The concentration of NEDD8-CUL2- ^Fluor568^RBX1 was first measured using the known extinction coefficient of the fluorescent dye and subsequently used to estimate the concentration of NEDD8-^Fluor568^CUL2-RBX1 by quantitative comparison of the protein levels in the 2-fold dilution series. All images can be found within the Mendeley data file (please refer to the key resources table).

##### Generation of ^Fluor647^UBE2R2^C93K^

^GGGG^UBE2R2^C93K^ was the substrate for a sortasing reaction in buffer (30 mM Tris pH 8.2, 150 mM NaCl, 6 mM CaCl_2_) that contained 40 μM ^GGGG^UBE2R2^C93K^, 15 μM sortase, and 100 μM peptide containing an N-terminal fluorescent dye and a C-terminal sortasing consensus site (Nterm-[C-647Mal]GSGGLPETGG-Cterm; Vivitide). The solution containing these components was incubated for 3 hours. The reaction was quenched with 10 mM DTT and passed through a 0.22 μm filter before purification by gel filtration (SuperDex 75) in storage buffer. Purified proteins were drop-frozen in liquid nitrogen and stored at −80°C. Generation of ^Fluor647^UBE2R2^wild-type^ was performed identically. The protein concentration was estimated by measuring the amount of light absorption at 280 nm and using the theoretical extinction coefficient based on the UBE2R2 protein sequence (note that here the fluorescence of the appended dye did not affect the absorption of light at 280 nm; ^Fluor647^UBE2R2^C93K^ appeared as a pure product as assessed by SDS-PAGE and detection of fluorescently labeled protein on a Typhoon 5 imager).

The biochemical activities of both NEDD8-^Fluor568^CUL2-RBX1 and ^Fluor647^UBE2R2^wild-type^ were assessed by single-encounter *in vitro* ubiquitylation assays. First, either NEDD8-CUL2-RBX1 or NEDD8-^Fluor568^CUL2-RBX1 were incubated with ELONGIN B/C-VHL (0.5 μM final) and ^32^P-labeled Hif1α peptide substrate (0.2 μM) to form a neddylated CRL2 substrate complex. In a separate tube, E1 (0.5 μM), K48R ubiquitin (2 μM) and UBE2R2 (1 μM) were incubated followed by initiation of the reaction by mixing with the E3-substrate tube. Time-points were taken and substrates and products were separated by SDS-PAGE and detected by autoradiography. Notice that the activities of wild-type and fluorescently labeled complex are well within 2-fold of each other. Next, the activity of ^Fluor647^UBE2R2^wild-type^ was estimated using the same assay and shows that fluorescently labeled UBE2R2 retains wild-type-like activity (note that the wild-type version was necessary as UBE2R2^C93K^ is inactive for neddylated CRL-substrate dependent ubiquitylation). All images can be found within the Mendeley data file (please refer to the key resources table).

##### Generation of UBE2R2^C93K^-UB

The covalent attachment of ubiquitin to the UBE2R2^C93K^ active site was accomplished as follows. UBE2R2^C93K^ was the substrate for a ubiquitylation reaction performed at 37°C overnight in loading buffer (100 mM Bis-Tris propane pH 9.5, 100 mM NaCl, 6 mM CaCl_2_ 10 mM MgCl_2_, and 2 mM ATP) that contained 5 μM UBA1, 20 μM UBE2R2^C93K^ and 100 μM K48R ubiquitin that contained an N-terminal 6-Histidine tag. The reaction was quenched with 10 mM DTT, before being diluted 1:4 in storage buffer and incubated with Ni-NTA resin for 2 hours. The bead solution was centrifuged at 1,500 x g for 2 minutes and the supernatant was discarded. Ni-NTA elution buffer was added (25 mM HEPES pH 8.0, 200 mM NaCl, and 300 mM imidazole) and incubated for 15 minutes with light agitation following collection of the eluate. The flow-through was then concentrated using a 10 kDa centrifugal filter (Millipore-Sigma), and then gel filtered over a SuperDex 75 column that had been equilibrated in storage buffer. Proteins were drop-frozen in liquid nitrogen and stored at −80°C. The protein concentration was determined as described above, except using the sum of the theoretical extinction coefficient values for both UBE2R2 and human ubiquitin (38,480 M^−1^cm^−1^). The final protein purity can be found within the Mendeley data file (please see the key resources table).

##### Fluorescence measurement and quantification

Equilibrium fluorescence measurements were obtained using a FluoroMax 4 Spectrofluorometer (Jobin Yvon). Varying concentrations of ^Fluor647^UBE2R2^C93K^ were first diluted in binding buffer (30 mM Tris pH 7.5, 100 mM NaCl, and 10% glycerol) in a final volume of 50 μL. In a separate tube, 200 μL solutions of NEDD8-^Fluor568^CUL2-RBX1 in the absence or presence of ELONGIN B/C-FEM1C were also prepared in binding buffer that had been supplemented with bovine serum Albumin (BSA; Omnipur) (30 mM Tris pH 7.5, 38 or 100 mM NaCl, 2 mg/mL BSA).

For binding reactions in a buffer containing a final NaCl concentration of 50 mM, note that the dilution of stock proteins that had been solubilized in storage buffer (containing 100 mM NaCl) was determined such that the final NaCl concentration would be 50 mM when diluted in binding buffer that contained 38 mM NaCl. These solutions were first mixed and then introduced to glass cuvettes (all binding reactions contained a final neddylated CRL concentration of 25 or 50 nM). All samples were excited at 575 nm, and the emission spectra were collected between 590 nm and 700 nm. Notice that the titration of ^Fluor647^UBE2R2^C93K^ resulted in sequentially lower fluorescence values of the donor dye appended to CUL2 as expected for energy transfer from the acceptor dye on UBE2R2 to the donor one (Figure S3A).

Importantly, the introduction of unlabeled wild-type UBE2R2 protein resulted in donor dye fluorescence levels that were similar to the values that had been observed in the absence of labeled UBE2R2 and acceptor dye (Figure S3B), demonstrating that the FRET signal most likely arises from *bona fide* protein-protein interactions instead of nonspecific aggregation. Eight measurements were taken for the titration of ^Fluor647^UBE2R2^C93K^. For each technical replicate, the fluorescence signal values were averaged from 599 nm and 601 nm which represented the peak of donor dye fluorescence. The FRET efficiency was then calculated as the absolute value of the fluorescence of the donor-acceptor pair minus the donor alone, which was then divided by the signal for donor alone. These values were plotted on a graph as a function of the ^Fluor647^UBE2R2^C93K^ concentration and the *K_d_* estimated by non-linear curve fitting to a one-site binding model (GraphPad Prism v10). Fluorescence measurements of the neddylated CRL2^FEM1C^ complex were performed as described above, but with unlabeled UBE2R2^wild-type^. The fluorescence values were normalized to the signal in the absence of UBE2R2 and then plotted against UBE2R2 protein levels followed by fitting the model as described above. For NEDD8-^Fluor568^CUL2-RBX1 fluorescence measurements in the presence of elongin B/C-FEM1C, the neddylated CRL complex was first reconstituted at 1 μM in binding buffer for 15 minutes before dilution to the final concentration (25 or 50 nM) in binding buffer.

##### Environmental sensitivity estimation

While attempting to estimate the *K_d_* of UBE2R2 for the neddylated CRL2^FEM1C^ complex, it was found that the addition of unlabeled UBE2R2 led to changes in NEDD8-^Fluor568^CUL2-RBX1 fluorescence values (Figure S3E). As such, environmental perturbation was employed to probe for binding of UBE2R2 to the neddylated CRL2^FEM1C^ complex in lieu of FRET. Control binding reactions were performed first in individual tubes, with UBE2D3, UBE2L3, or UBE2R2 being diluted into storage buffer (30 mM Tris pH 7.5, 100 mM NaCl, 10% glycerol, 1 mM DTT) to a final volume of 50 μL and incubated for 15 minutes at room temperature (final concentrations were 50 μM for UBE2D3 and UBE2L3, and 10 μM for UBE2R2). For reactions involving ARIH1, the following reagents were first diluted into a modified 1X reaction buffer (30 mM Tris pH 7.5, 38 mM NaCl, 5 mM MgCl_2_, 2 mM DTT, and 2 mM ATP) and incubated for 15 minutes at room temperature also in a volume of 50 μL: human E1 (2.5 μM), wild-type ubiquitin (20 μM), UBE2L3 (25 μM) and ARIH1 (10 μM). Concurrently, NEDD8-^Fluor568^CUL2-RBX1 and ELONGIN B/C-FEM1C were diluted into binding buffer (30 mM Tris pH 7.5, 38 mM NaCl, and 2 mg/mL BSA) in a total volume of 5 μL and incubated at room temperature for 15 minutes. Next, the neddylated CRL2 mixtures were diluted 1:40 and then mixed with the ubiquitin-carrying enzyme samples for a final volume of 250 μL. Measurements were taken, and normalized fluorescence was estimated (Figure S3F). A similar protocol was followed for the UBE2R2 titrations and estimation of the *K_d_* of UBE2R2 for the neddylated CRL2^FEM1C^ complex.

#### Cryo-EM structure determination

##### Activity-based probe formation

Activity-based probes were used to mimic the covalent attachment of a donor ubiquitin to UBE2R2 and catalysis of the transfer of ubiquitin to neddylated CRL-bound peptide substrate Sil1 or neo-substrate BRD4 (346–460). Collectively, the UBE2R2~ubiquitin-substrate complex is referred to as the ‘trap’ since it is expected to form avid interactions with the NEDD8-CRL2 complexes. Briefly, ubiquitin BMDPa^39^ (0.5 mg/ml, final concentration) was reacted with either freshly dissolved Sil1 peptide (N-term-CEGYFQELLGSVNPTQGRAR-C-term; 1:0.9 molar ratio) or 100 μM K368C C356A C357A C391A C429A BRD4 (346–460), where the ubiquitylation site (Lys368) had been replaced with a Cys residue to promote cross-linking. The reaction was incubated for 15 minutes (Sil1-ubiquitin activity-based probe) or 1 hour (BRD4 (346–460)-ubiquitin activity-based probe) at 30°C and followed immediately by size-exclusion chromatography over a SuperDex 75 gel filtration column that had been equilibrated in a buffer containing 25 mM HEPES pH 7.5 and 150 mM NaCl.

##### Trapped complex formation

To form the trapped priming complexes (NEDD8-CRL2^FEM1C-Sil1^-UBE2R2~UB) for both cryo-EM and PhoX cross-linking followed by mass spectrometry and NEDD8-CRL2^VHL-MZ1-BRD4 (346−460)^-UBE2R2~ubiquitin for cryo-EM, UBE2R2 was incubated with 1 mM TCEP for 20 minutes on ice and then desalted (Zeba desalting columns, Thermo Fisher) into a buffer containing 25 mM HEPES pH 7.5 and 150 mM NaCl. Next, desalted UBE2R2 was immediately added to NEDD8-CRL2^FEM1C^ or NEDD8-CRL2^VHL-MZ1^ (both 7.5 μM final) and a 6-fold molar excess of Sil1-ubiquitin or BRD4 (346–460)-ubiquitin activity-based probes, respectively, for 30 minutes at 30°C. The trapped neddylated CRL2 complexes were purified by size-exclusion chromatography. The buffer for mass spectrometry contained 25 mM HEPES pH 7.5, 100 mM NaCl, and 1 mM TCEP, whereas the buffer for cryo-EM contained 25 mM HEPES pH 7.5, 75 mM NaCl, and 1 mM TCEP.

##### Cryo-EM

3.5 μL of purified and trapped complexes (0.8 mg/mL) was applied onto R1.2/1.3 holey carbon grids (Quantifoil). Immediately following the first application of sample to the grid, the remaining protein solution was removed with a pipette, immediately followed by application of another 3.5 μL of trapped complex onto the grids and blotted with Whatman filter paper using a blot force of 4 for 3.5 seconds using a Vitrobot Mark IV (4°C, 100% humidity). Grids were plunge-frozen in liquid ethane. The low-resolution dataset was collected on a 200 kV Glacios transmission electron microscope (TEM) equipped with a K2 direct detector set to counting mode. 1,984 micrographs were collected at 1.885Å pixel size, with a total exposure set to 60 electrons Å^−2^ (40 frames). The defocus value ranged from −0.8 and −3.2 μm. 17,719 and 15,758 high-resolution micrographs were collected on a 300 kV Titan Krios TEM at 0.851Å pixel size for NEDD8-CRL2^FEM1C-Sil1^-UBE2R2~ubiquitin and NEDD8-CRL2^VHL-MZ1-BRD4 (346−460)^-UBE2R2~ubiquitin complexes, respectively. The microscope was equipped with a K3 direct detector set to counting mode. The total exposure was set to 66 electrons Å^−2^ (38 frames) and the defocus value ranged from −0.6 to −2.6 μm.

##### Cryo-EM data processing

The Glacios and Titan Krios datasets were processed in RELION 3.1.1.^96^ First, the raw movie frames were aligned and dose-weighted using MotionCorr2.^97^ Next, Gctf was employed to estimate the contrast-transfer-function.^100^ Particle picking was performed using Gautomatch.^100^ *Ab initio* reconstruction was performed in cryoSPARC.^95^ All further operations including 2D and 3D classification, global and local focused 3D refinement, as well as post-processing were done in RELION 3.1.1. Moreover, the final maps were post-processed in DeepEMhancer.^101^ For all datasets, DeepEMhancer-derived maps were uploaded to EMDB as the main maps, whereas RELION post-processed maps were also uploaded as additional maps. To better extract high-resolution features for the UBE2R2-FEM1C interface corresponding to the NEDD8-CRL2^FEM1C-Sil1^-UBE2R2~ubiquitin complex in the Titan Krios dataset, a focused map was generated, masking on: CUL2 (residues 1–511), ELONGIN B/C-FEM1C (residues 245-C), and UBE2R2 (Figure S4). Similarly, a focused map was generated for the NEDD8-CRL2^VHL-MZ1-BRD4 (346−460)^-UBE2R2~ubiquitin complex, masking on: CUL2 (residues 384–647), UBE2R2, RBX1, ubiquitin, BRD4 (346–460), MZ1 and VHL (residues 61–152) (Figure S6).

##### Model building and refinement

###### NEDD8-CRL2^FEM1C-Sil1^-UBE2R2~ubiquitin.

An initial model was created by docking previously reported structures of sub-complexes or single components, consisting of UBE2R2 (PDB: 6NYO), donor ubiquitin (PDB: 8PQL), CUL2-ELONGIN B/C (PDB: 5N4W), and FEM1C residues 245–373 (PDB: 6LBN). The latter coordinates were combined with an AlphaFold model^111^ for FEM1C’s residues 374 to the C terminus to form a single unit corresponding to FEM1C residues 245 through the C terminus (245-C). First, the components of the structure that were resolved to high-resolution (3.6Å), corresponding to CUL2 residues 1–511, ELONGIN B/C-FEM1C (residues 245-C), and UBE2R2, were built using both the focused and consensus maps (EMDB: EMD-18230) as follows. The coordinates for these units were first manually placed into the cryo-EM density followed by rigid-body refinement with UCSF Chimera. Next, the remaining density was manually fitted with models for FEM1C residues 1–244, Sil1 substrate peptide, donor ubiquitin, RBX1 and CUL2 residues 512–644, based on the PDB files PDB: 6LBN for FEM1C, PDB: 8PQL for Sil1 and donor ubiquitin, PDB: 6TTU for RBX1, and an AlphaFold model for CUL2 residues 512–644, followed by rigid-body refinement using UCSF Chimera and the lower-resolution Glacios map (EMDB: EMD-18207). The chemical ligand used to cross-link UBE2R2, donor ubiquitin and substrate (identified in the PDB file by the compound ‘SY8’) was first modelled in the correct orientation and in proximity with UBE2R2’s active site Cys. Here it was also necessary to estimate the position of a single Cys residue corresponding to Sil1’s N terminus to complete the three-way cross-link, owing to an absence of electron density in this region as well as an absence of ordered residues from the coordinates used to initially model Sil1 (PDB: 8PQL). Iterative rounds of manual model building in COOT^104^ and real-space refinement with Phenix.refine^105^ were performed until good geometries and map-to-model correlations were achieved. Side-chains were built for residues when the cryo-EM maps showed correspondingly well-resolved density. In cases where the electron density was ambiguous, side-chain rotamers were maintained from the original structures used to general the model (FEM1C residues 1–244 (PDB: 6LBN), Sil1 and donor ubiquitin (PDB: 8PQL), RBX1 (PDB: 6TTU), and CUL2 residues 512–644 derived from an AlphaFold model). Sil1 residues 456–460 were not included in the model owing to a lack of density from the Krios map. The final structure was refined using the consensus map. As stated above, the DeepEMhancer processed maps have been deposited as the primary maps in the Electron Microscopy Data Bank (EMDB: EMD-18207 and EMDB: EMD-18230), while the post-processed maps can be found under the ‘additional data’ tab.

###### NEDD8-CRL2^VHL-MZ1-BRD4 (346−460)^-UBE2R2~ubiquitin.

An initial model was created using previously solved structures of sub-complexes or single components, consisting of CUL2 (PDB: 5N4W and PDB: 8PQL), ELONGIN B/C-VHL-MZ1-BRD4 (PDB: 5T35), and UBE2R2-ubiquitin-RBX1 (PDB: 8PQL). First, CUL2 (residues 1–511), ELONGIN B/C-VHL-MZ1, and BRD4 (residues 346–460), which were resolved to high-resolution (3.4Å), were built using both the focused and consensus maps (EMDB: EMD-18915). The remaining elements of the structure, including UBE2R2, ubiquitin, RBX1 and CUL2 (residues 512–644) (PDB: 8PQL) were docked into the density using rigid-body refinement in UCSF Chimera. Minor modifications were made in the docked proteins using COOT which were also allowed to move during refinement in Phenix. In this case, the three-way cross-link junctions were visible in the electron density maps and used to place the compound (SY8) relative to UBE2R2, donor ubiquitin and BRD4’s Cys 368. Additional electron density was visible in CUL2’s basic canyon that was consistent with the C-terminal acidic tail of UBE2R2. Since sidechain atoms could not be unambiguously assigned, the backbone atoms were built for 15 consecutive residues (kept as part of UBE2R2’s chain but the sequence was not defined and thus residues names are unknown (UNK)). Similarly as above, iterative rounds of manual model building in COOT^104^ and real-space refinement with Phenix.refine^105^ were performed until good geometries and map-to-model correlations were achieved. Parts of protein subunits that lacked clear electron density were excluded from the model. Side-chains were built for residues when the cryo-EM maps showed correspondingly well-resolved density. In cases where the electron density was ambiguous, side-chain rotamers were maintained from the original structures used to generate the model (UBE2R2, donor ubiquitin and RBX1 (PDB: 8PQL) and CUL2 residues 512–644 derived from an AlphaFold model). The final structure was refined using the consensus map. As described above, the DeepEMhancer processed map has been deposited as the primary map in the Electron Microscopy Data Bank (EMDB: EMD-18915), while the post-processed maps can be found under the ‘additional data’ tab.

#### PhoX protein-protein cross-linking

Three distinct CRL2-FEM1C-ubiquitin-carrying enzyme complexes were subjected to PhoX-mediated protein cross-linking: (1) CRL2^FEM1C^ in the presence of UBE2R2; (2) neddylated CRL2^FEM1C^ in the presence of UBE2R2 and Sil1 peptide substrate; and (3) the ‘trapped’ complex that contained neddylated CRL2^FEM1C^ in the presence of cross-linked UBE2R2~UB-Sil1. In all cases, 100 μg of each complex was incubated with 2.5 mM PhoX cross-linker (50 mM stock in DMSO; Bruker Daltonics, product number 1881358)^73^ for 30 minutes at room temperature. Reactions were quenched with 20 mM Tris-HCl pH 7.5 and submitted for analysis. All experiments were performed in duplicate.

Cross-linked proteins were denatured by addition of 4 M Urea in 50 mM Tris. For reduction and alkylation of the proteins, 40 mM 2-chloroacetamide (CAA; Sigma-Aldrich) and 10 mM tris(2-carboxyethyl)phosphine (TCEP; Thermo Fisher Scientific) were added. After incubation for 20 minutes at 37°C, the samples were diluted 1:3 with MS grade water (VWR). Proteins were digested overnight at 37°C by addition of 1 μg of LysC and 2 μg of trypsin (Promega). Thereafter, the solution was acidified with trifluoroacetic acid (TFA; Merck) to a final concentration of 1%, followed by desalting of the peptides using Sep-Pak C18 1cc vacuum cartridges (Waters). PhoX cross-linked peptides were enriched with Fe(III)-NTA cartridges (Agilent Technologies; Santa Clara, CA) using the AssayMAP Bravo Platform (Agilent Technologies; Santa Clara, CA) on a liquid sample handling platform enabling high sample throughput.^73^

Enriched peptides were dissolved in Buffer A (0.1% formic acid) and 1/10 of the peptides were analyzed using LC-MS/MS. Pep tides were separated on a 30cm analytical column (inner diameter: 75 microns; packed in-house with ReproSil-Pur C18-AQ 1.9-micron beads; Dr. Maisch GmbH) by an Easy-nLC 1200 (Thermo Fisher Scientific) coupled to an Exploris 480 mass spectrometer (Thermo Fisher Scientific) at a flow rate of 300 nL/min. The analytical column was heated to 60°C. For the elution gradient, the following steps were programmed with increasing addition of Buffer B (80% Acetonitrile, 0.1% formic acid): linear increase from 5% to 30% over 40 minutes, followed by a linear increase to 95% over 10 minutes, and finally, the percentage of Buffer B was maintained at 95% for another 10 minutes. The mass spectrometer was operated in data-dependent mode with survey scans from m/z 300 to 1,650 Th (resolution of 60k at m/z = 200 Th), and up to 15 of the most abundant precursors were selected and fragmented using stepped Higher-energy C-trap Dissociation (HCD with a normalized collision energy of value of 19, 27, 35). The MS2 spectra were recorded with dynamic m/z range (resolution of 30k at m/z = 200 Th). Normalized AGC targets for MS1 and MS2 scans were set to 300% and 100%, respectively, within a maximum injection time of 25 milliseconds for the MS1 scan. The maximum injection time was set to “auto” for the MS2 scans. Charge state 2 was excluded from fragmentation to enrich the fragmentation scans for cross-linked peptide precursors.

The acquired raw data were processed using Proteome Discoverer (version 2.5.0.400) with the XlinkX/PD nodes integrated.^112^ To identify the cross-linked peptide pairs, a database search was performed against a FASTA containing the sequences of the proteins under investigation. PhoX was set as a cross-linker. Cysteine carbamidomethylation was set as fixed modification and methionine oxidation and protein N-term acetylation were set as dynamic modifications. Trypsin/P was specified as the protease and up to two missed cleavages were allowed. Furthermore, identifications were only accepted with a minimal score of 40 and a minimal delta score of 4. Filtering at 1% false discovery rate (FDR) at peptide level was applied.

### QUANTIFICATION AND STATISTICAL ANALYSIS

#### Statistical analysis and quantification

For quantification of *in vitro* reconstituted ubiquitylation assays, the intensities of radiolabeled peptide or protein substrates and their ubiquitylated forms were imaged by scanning SDS-PAGE gels on a Typhoon 5 (Cytiva) and quantified using ImageQuant software. Experiments measuring the Michaelis constant *K_m_* were fit using nonlinear regression on GraphPad Prism v10, while *k_obs_* was analyzed using Mathematica v13.1. All data were measured in triplicate technical replicates. Binding assays were fit using one-phase association in GraphPad Prism v10 and performed in triplicate technical replicates, with data being represented as the average of each replicate, and the error representing the standard error. Statistical analysis and significance in the cellular PROTAC-dependent neo-substrate degradation assay was determined using an unpaired t-test with Welch’s correction. Statistical parameters that were reported in the figures are described in the corresponding figure legend.

## Supplementary Material

MMC3

MMC5

MMC4

MMC2

MMC1

## Figures and Tables

**Table 1.** Cryo-EM data collection, refinement, and validation statistics

Complex	NEDD8-CRL2^vhl-mz1-brd4 BD2^-UBE2R2~ubiquitin	NEDD8-CRL2^FEM1C-Sil1^-UBE2R2~ubiquitin	NEDD8-CRL2^FEM1C-Sil1^-UBE2R2~ubiquitin

	EMDB: EMD-18915; PDB: 8R5H	EMDB: EMD-18230; PDB: 8Q7R	EMDB: EMD-18207
Microscope	Krios	Krios	Glacios
Magnification	130,000	130,000	22,000
Voltage (kV)	300	300	200
Electron exposure (e^−^/Å^2^)	66	66	60
Defocus range (μm)	−0.6 ~ −2.6	−0.6 ~ −2.6	−0.8 ~ −3.2
Pixel size (Å)	0.8512	0.8512	1.885
Symmetry imposed	C1	C1	C1
Initial particle images (no.)	6,463,393	4,475,838	1,983,382
Final particle images (no.)	148,402	135,564	70,503

Map resolution (Å)			

FSC threshold	3.44 (0.143)	3.71^a^ (0.143)	6.46(0.143)
Map resolution range (Å)	-	-	-

Refinement			
Initial model used (PDB code)	PDB: 5N4W, 5T35, 8PQL	PDB: 5N4W, 6NYO, 6TTU, 6LBN, 8PQI	-

Model resolution (Å)			

FSC threshold	(0.143)	3.71 (0.143)	-
Model resolution range (Å)	-	_-_	-
Map sharpening *B* factor (Å^2^)	−30	−40^b^	−200

Model composition			

Non-hydrogen atoms	11,277	12,536	-
Protein residues	1,437	1,726	-
Ligands	3(ZN)	3(ZN)	-

*B* factors (Å^2^)			

Protein	68.79	59.66	-
Ligand	98.39	92.01	-

RMSDs			

Bond lengths (Å)	0.003	0.004	-
Bond angles (°)	0.681	0.740	-

Validation			

MolProbity score	1.87	2.07	-
Clashscore	6.74	9.49	-
Poor rotamers (%)	0.0	1.26	-

Ramachandran plot			

Favored (%)	91.58	91.82	-
Allowed (%)	8.42	8.18	-
Disallowed (%)	0.00	0.00	-

Related to Figures 5, 6, S4, and S6 and STAR Methods.

a Consensus map: 3.71 Å; focused map: 3.63 Å.

b Consensus map: −40; focused map: −80.

**Table:** KEY RESOURCES TABLE

REAGENT or RESOURCE	SOURCE	IDENTIFIER
Antibodies		

Rabbit monoclonal anti-BRD2 antibody	Cell Signaling Technology	Cat#5848; RRID: AB_10835146
Mouse monoclonal anti-BRD3 antibody	Santa Cruz	Cat#sc81202; RRID: AB_1119692
Rabbit monoclonal anti-BRD4 antibody	Cell Signaling Technology	Cat#13440; RRID: AB_2687578
Rabbit monoclonal anti-ZER1 antibody	Invitrogen	Cat#PA5-21807; RRID: AB_11153226
Rabbit monoclonal anti-KLHDC2 antibody	Prestige	Cat#HPA000628; RRID: AB_2666051
Mouse monoclonal anti-GAPDH antibody	Abcam	Cat#ab9484; RRID: AB_307274
Mouse monoclonal anti-GAPDH antibody	Santa Cruz	Cat#sc32233; RRID: AB_627679
Rabbit monoclonal anti-UBE2R1 antibody	Abcam	Cat#ab204515
Rabbit monoclonal anti-UBE2R2 antibody	Santa Cruz	Cat#sc134628; RRID: AB_2010705
Mouse monoclonal anti-β-ACTIN antibody	Millipore-Sigma	Cat#A1978; RRID: AB_476692
Monoclonal anti-ARIH1 antibody	Kelsall et al.^29^	N/A
Goat anti-rabbit IgG HRP secondary antibody	Bio-Rad	Cat#1706515; RRID: AB_11125142
Goat anti-mouse IgG HRP secondary antibody	Bio-Rad	Cat#1721011; RRID: AB_11125936

Bacterial and virus strains		

*E. coli* BL21 (DE3)	New England Biolabs	Cat#C2527H
*E. coli* Rosetta (DE3)	Sigma-Aldrich	Cat#71400-3
*E. coli* DH5α (DE3)	New England Biolabs	Cat#C2987H

Chemicals, peptides, and recombinant proteins		

ARV-771	MedChemExpress	Cat#HY-100972
MZ1	MedChemExpress	Cat#HY-107425
dBET1	MedChemExpress	Cat#HY-101838
Wild-type Ubiquitin	R&D Systems	Cat#U-100H-10M
Hif1 α peptideAc-KLRREPDALTLLA(hyP) AAGDTIISLDFGSNGRRASY-OH	New England Peptide	N/A
Sil1 peptide (for kinetic assays)Ac-GRRASYGSGSKEGYFQELLGSVNPTQGRAR-OH	Vivitide	N/A
Sil1 peptide (for cryo-EM) H_2_N-CEGYFQELLGSVPTQGRAR-OH	Max Planck Institute für Biochemie	N/A
AZ-Dye 568H_2_N-(C-AZDye568Mal) GSGGLPETGG-OH	Vivitide	N/A
AZ-Dye 647H_2_N-(C/AZDye647Mal) GSGGLPETGG-OH	Vivitide	N/A
cAMP-dependent Protein Kinase (PKA), catalytic subunit	New England Biolabs	Cat#P6000S
Halt^™^ Protease and Phosphatase Inhibitor Cocktail	Thermo-Fisher	Cat#78440
cOmplete^™^ EDTA-free Protease Inhibitor Cocktail	Roche	Cat#11873580001
NEBuffer^™^ for Protein Kinases (PK)	New England Biolabs	Cat#B6022S
Adenosine 5′-triphosphate,[γ−^32^P]-	Perkin Elmer	NEG002A100UC
Trypsin, porcine, Proteomics Grade	Sigma Aldrich	T6567
Trypsin, sequencing grade	Promega	Cat#V5111
Trypsin/Lys-C Mix	Promega	Cat#V5071
Asp-N	Promega	Cat#VA1160
Glu-C	Creative Biolabs	Cat#Glyco-079CL
ReproSil-Pur C18-AQ 1.9 μm resin	Dr. Maisch GmbH	Cat#r119.aq.
2-chloroacetamide	Sigma-Aldrich	Cat#C0267

Critical commercial assays		

QIAquick Gel Extraction Kit	Qiagen	Cat#28706X4
E.Z.N.A Plasmid DNA Mini Kit	Omega Bio-Tek	Cat#D6942-00S
Pierce^™^ BCA Protein Assay Kit	Thermo-Fisher	Cat#23225
Lenti-X GoStix Plus	Takara Bio	Cat#631280

Deposited data		

Atomic model of SKP1-FBXW1A-IκBα-UB~UBE2D2	Baek et al.^38^	PDB: 6TTU
Atomic model of ELONGIN B/C-FEM1C-Sil1-UB-UBE2R2	Liwocha et al.^81^	PDB: 8PQL
ELONGIN B/C-FEM1C-Sil1-UBE2R2 (Krios)	Electron Microscopy Data Bank	PDB: 8Q7R EMDB: EMD-18230
ELONGIN B/C-VHL-MZ1-BRD4-UBE2R2 (Krios)	Electron Microscopy Data Bank	PDB: 8R5H EMDB: EMD-18915
ELONGIN B/C-FEM1C-Sil1-UBE2R2 (Glacios)	Electron Microscopy Data Bank	EMDB: EMD-18207
Raw image data	This study	Mendeley data: https://doi.org/10.17632/vkcnhm7rwc.1
Proteomics data	This study	Excel File S1 PRIDE: PXD043523

Experimental models: Cell lines		

HEK 293T/17	Hill et al.^37^	Clone identifiers G3, D5
HEK 293T/17 *UBE2R1/UBE2R2* DKO	Hill et al.^37^	Clone identifiers B3, E4, A10
HEK 293T/17 *Δ*UBE2G1	This study	Clone identifiers 2, 5, 35
Flp-In T-REx HEK293+GFP-ARIH1	This study	N/A
High-Five Insect cells	Thermo Fisher	Cat#B85502
Sf9 Insect cells	Thermo Fisher	Cat#11496015
MRC-5	Coriell Institute	Cat#AG05965-D RRID: CVCL_H748
T-47D	ATCC	Cat#HTB-133 RRID: CVCL_0553
NIH/3T3	ATCC	Cat#CRL-1658 RRID: CVCL_0594
MCF7	ATCC	Cat#HTB-22 RRID: CVCL_0031
HCC1954	ATCC	Cat#CRL-2338 RRID: CVCL_1259
MDA-MB-231	ATCC	Cat#HTB-26 RRID: CVCL_0062
MDA-MB-468	ATCC	Cat#HTB-132 RRID: CVCL_0419
AU565	ATCC	Cat#CRL-2351 RRID: CVCL_1074
A-172	ATCC	Cat#CRL-1620 RRID: CVCL_0131
HT29	ATCC	Cat#HTB-38 RRID: CVCL_0320
SHSY5Y	ATCC	Cat#CRL-2266 RRID: CVCL_0019
U2OS	ATCC	Cat#HTB-96 RRID: CVCL_0042
HAP1	Horizon Discovery	Cat#C631 RRID: CVCL_Y019
EW16	Children′s Oncology Group	N/A

Oligonucleotides		

siARIH1 (5′ CGAGAUAUUUCCCAAG AUU 3′)	This study	N/A
*UBE2G1* guideRNA DNA sequence (5′ ACTGCTACTGCGAAGACAGC 3′)	This study	N/A
*UBE2G1* single-stranded oligo for homologous recombination (5′ GGACTGGGCTGCGGCTGTCCGCGATCGCGGCCGGGCCCGGCGCCCCGCCGCCCGCCTGCTCACCTGCTAACTAACTATTAATTAATTATCAATCAA]TCACTAACTAACTATTAATTAATTATCAATCAATCAAAGTCTTCGCAGTAGCAGTGCCGACTGCAGCTCCGTCATCCTCCCTGCCGAGGGCCCGGGCTGGCGCC 3′)	This study	N/A
*UBE2G1* CRISPR PCR primer 1 - forward5′ GCCGGATCCGAAGCGAGCGGACTCGCAC 3′	This study	N/A
*UBE2G1* CRISPR PCR primer 2 - reverse5′- GCCGAATCCCCCGGGAGGAGAAGAGGGACT −3′	This study	N/A

Recombinant DNA		

pET11b-UBE2R2	Hill et al.^37^	N/A
pET11b-GGGG-UBE2R2	This study	N/A
pET11b-GGGG-UBE2R2(C93K)	This study	N/A
pET11b-6xHis-human-K0-ubiquitin	Hill et al.^37^	N/A
pET11b-6xHis-human-K48R-ubiquitin	Hill et al.^37^	N/A
pET11b-6xHis-human-D77-ubiquitin	Ziemba et al.^90^	N/A
pGEX-4T1 GST-TEV-ARIH1	Hill et al.^37^	N/A
pGEX-4T1 GST-TEV-UBE2L3	Hill et al.^37^	N/A
pGEX-4T1 GST-TEV-UBE2G1	This study	N/A
pGEX-4T1 GST-TEV-UBE2D3	This study	N/A
pGEX-4T1 GST-TEV-FEM1C	This study	N/A
pGEX-4T1 GST-TEV-VHL (54-C)	This study	N/A
pGEX-4T1 GST-TEV-BRD4^bd2^ (346-460)	This study	N/A
pX330	Cong et al.^91^	Cat#42230 RRID: Addgene_42230
pFastbac GST-TEV-RBX1	Scott et al.^30^	N/A
psPAX.2	Didier Trono	RRID: Addgene_12260
pMD2.G	Didier Trono	RRID: Addgene_12259
pLIB	Weissmann et al.^92^	N/A
pLIB GST-TEV-UBA1	Baek et al.^38^	N/A
pLIB His-TEV-DAC-CUL2	Scott et al.^93^	N/A
pRK793 TEV protease	David Waugh	RRID: Addgene_8827
pGEX-4T1 GST-Thrombin-UBE2M	Duda et al.^11^	N/A
pGEX-4T1 GST-Thrombin-APPBP1-UBA3	Duda et al.^11^	N/A
pGEX-4T1 GST-Thrombin-NEDD8 (S>C) Insert GSGS @72	Scott et al.^88^	N/A
pGEX His-RBX1-StrepII-CUL2	Diaz et al.^94^	N/A
pGEX His-RBX1-StrepII-GGGG-CUL2	This study	N/A
pACYC-ELONGIN B/C	This study	N/A

Software and algorithms		

Prism v10.0.0.153	GraphPad	https://www.graphpad.com/
Mathematica v13.1	Wolfram	https://www.wolfram.com/mathematica/
ImageQuant	Cytiva	https://www.cytivalifesciences.com/
cryoSPARC	Punjani et al.^95^	https://www.cryosparc.com/
RELION v3.1.1	Zivanov et al.^96^	https://www3.mrc-lmb.cam.ac.uk/relion
MotionCor2 v1.1	Zheng et al.^97^	https://msg.ucsf.edu/em/software/index.html
UCSF Chimera v1.11.2	University of California, San Francisco; Pettersen et al.^98^	https://www.cgl.ucsf.edu/chimera/
FluorEssence	Horiba Scientific	https://www.horiba.com/int/scientific/products/detail/action/show/Product/fluoressence-1378/
Proteome Discoverer v2.5.0.400	Thermo Fisher	https://www.thermofisher.com/
Serial-EM v3.8.0-b5	N/A	https://bio3d.colorado.edu/SerialEM/
FEI EPU v2.7.0	Thermo Scientific	https://www.thermofisher.com/
Typhoon FLA Phosphoimager	General Electric	https://www.cytivalifesciences.com/
Gautomatch v0.56	Kai Zhang	https://www2.mrc-lmb.cam.ac.uk/download/gautomatch-056/
CTFFIND v4.1	Rohou and Grigorieff^99^	https://grigoriefflab.umassmed.edu/ctffind4
GCTF v1.06	Zhang^100^	https://www2.mrc-lmb.cam.ac.uk/download/gctf/
DeepEMhancer	Sanchez-Garcia et al.^101^	https://github.com/rsanchezgarc/deepEMhancer
Focus v1.2	Biyani etal.^102^	https://lbem-focus.epfl.ch/documentation.php
ChimeraX v1.2	Goddard et al.^103^	https://www.rbvi.ucsf.edu/chimerax/
PyMOL v2.3.3	Schrodinger, LLC	https://pymol.org/2/
COOT v0.8.9.1	Emsley et al.^104^	https://www2.mrc-lmb.cam.ac.uk/personal/pemsley/coot/
Phenix.refine v1.19.2	Afonine et al.^105^	https://www.phenix-online.org/
MaxQuant (version 1.6.7.0)	Tyanova	https://www.maxquant.org/maxquant/

Other		

RNA-Max Lipofectamine	Thermo Fisher	Cat#13778150
DMEM, high glucose, pyruvate	Thermo-Fisher	Cat#11965092
Sf-900 III SFM Media	Gibco	Cat#12658019
ESF-921 Media	Expression Systems	Cat#96-001-01
Fetal Bovine Serum	Fisher-Scientific	Cat#10437028
Fetal Bovine Serum	Atlanta Biologicals	Cat#S12550
GlutaMax	Thermo-Fisher	Cat#35050061
Penicillin Streptomycin	Gibco	Cat#15140122
Zeocin^™^	Gibco	Cat#R25001
Hygromycin B	Thermo-Fisher	Cat#10687010
BlasticidinS-HCl	Gibco	Cat#A1113903
PhoX Cross-linker	Bruker Daltonics	Cat#1881358
Fe(III)-NTA cartridges	Agilent Technologies	Cat#G5496-60085
HaloLink^™^ Resin	Promega	Cat#G1912
Blotting Grade Blocker Nonfat Dry Milk Powder	Bio-Rad	Cat#1706404XTU
SuperSignal^™^ West Pico PLUS Chemiluminescent Substrate	Thermo-Fisher	Cat#34580
Strep-Tactin Sepharose resin	IBA Lifesciences	Cat#2-1201-002
Ni-NTA agarose	Qiagen	Cat#30210
Glutathione Sepharose 4B resin	Cytiva	Cat#17075605
R1.2/1.3 holey carbon grids	Quantifoil	Cat#4220C-CF
TMT10plex Isobaric Label Reagents	Thermo Scientific	Cat#90110
Lysyl Endopeptidase, Mass Spectrometry Grade	Fujifilm WAKO	Cat#125-05063
Bovine serum albumin	Omnipur	Cat#2905-OP
Sep-Pak C18 3 cc Vac Cartridge	Waters	Cat#WAT036945
AssayMAP Bravo Platform	Agilent	Cat#G5571AA

## Highlights

- UBE2R E2s preferentially target CUL2-based CRL2 substrates
- CRL2-dependent PROTAC efficiency correlates with UBE2R-family E2 levels in cells
- Cryo-EM reveals a UBE2R2-CRL2 interface that underlies geometric substrate selection
- A dedicated poly-ubiquitin chain extending E2 is reprogrammed to prime CRL2 substrates

## References

[1] Willems AR, Schwab M, and Tyers M. (2004). A hitchhiker’s guide to the cullin ubiquitin ligases: SCF and its kin. Biochim. Biophys. Acta 1695, 133–170. 15571813 10.1016/j.bbamcr.2004.09.027

[2] Frescas D, and Pagano M. (2008). Deregulated proteolysis by the F-box proteins SKP2 and [beta]-TrCP: tipping the scales of cancer. Nat. Rev. Cancer 8, 438–449. 18500245 10.1038/nrc2396 PMC2711846

[3] Liu Y, and Tan X. (2020). Viral Manipulations of the Cullin-RING Ubiquitin Ligases. Adv. Exp. Med. Biol 1217, 99–110. 31898224 10.1007/978-981-15-1025-0_7

[4] Teng M, and Gray NS (2023). The rise of degrader drugs. Cell Chem. Biol 30, 864–878. 10.1016/j.chembiol.2023.06.020 37494935

[5] Wu T, Yoon H, Xiong Y, Dixon-Clarke SE, Nowak RP, and Fischer ES (2020). Targeted protein degradation as a powerful research tool in basic biology and drug target discovery. Nat. Struct. Mol. Biol 27, 605–614. 32541897 10.1038/s41594-020-0438-0 PMC7923177

[6] Burslem GM, and Crews CM (2020). Proteolysis-Targeting Chimeras as Therapeutics and Tools for Biological Discovery. Cell 181, 102–114. 31955850 10.1016/j.cell.2019.11.031 PMC7319047

[7] Cowan AD, and Ciulli A. (2022). Driving E3 Ligase Substrate Specificity for Targeted Protein Degradation: Lessons from Nature and the Laboratory. Annu. Rev. Biochem 91, 295–319. 35320687 10.1146/annurev-biochem-032620-104421

[8] Deshaies RJ (2020). Multispecific drugs herald a new era of biopharmaceutical innovation. Nature 580, 329–338. 32296187 10.1038/s41586-020-2168-1

[9] Bulatov E, and Ciulli A. (2015). Targeting Cullin-RING E3 ubiquitin ligases for drug discovery: structure, assembly and small-molecule modulation. Biochem. J 467, 365–386. 25886174 10.1042/BJ20141450 PMC4403949

[10] Pan ZQ, Kentsis A, Dias DC, Yamoah K, and Wu K. (2004). Nedd8 on cullin: building an expressway to protein destruction. Oncogene 23, 1985–1997. 15021886 10.1038/sj.onc.1207414

[11] Duda DM, Borg LA, Scott DC, Hunt HW, Hammel M, and Schulman BA (2008). Structural insights into NEDD8 activation of cullin-RING ligases: conformational control of conjugation. Cell 134, 995–1006. 18805092 10.1016/j.cell.2008.07.022 PMC2628631

[12] Fischer ES, Scrima A, Böhm K, Matsumoto S, Lingaraju GM, Faty M, Yasuda T, Cavadini S, Wakasugi M, Hanaoka F, (2011). The molecular basis of CRL4DDB2/CSA ubiquitin ligase architecture, targeting, and activation. Cell 147, 1024–1039. 22118460 10.1016/j.cell.2011.10.035

[13] Emberley ED, Mosadeghi R, and Deshaies RJ (2012). Deconjugation of Nedd8 from Cul1 is directly regulated by Skp1-F-box and substrate, and the COP9 signalosome inhibits deneddylated SCF by a noncatalytic mechanism. J. Biol. Chem 287, 29679–29689. 22767593 10.1074/jbc.M112.352484 PMC3436198

[14] Mosadeghi R, Reichermeier KM, Winkler M, Schreiber A, Reitsma JM, Zhang Y, Stengel F, Cao J, Kim M, Sweredoski MJ, (2016). Structural and kinetic analysis of the COP9-Signalosome activation and the cullin-RING ubiquitin ligase deneddylation cycle. eLife 5, e12102. 27031283 10.7554/eLife.12102 PMC4878873

[15] Enchev RI, Scott DC, da Fonseca PC, Schreiber A, Monda JK, Schulman BA, Peter M, and Morris EP (2012). Structural basis for a reciprocal regulation between SCF and CSN. Cell Rep. 2, 616–627. 22959436 10.1016/j.celrep.2012.08.019 PMC3703508

[16] Cavadini S, Fischer ES, Bunker RD, Potenza A, Lingaraju GM, Goldie KN, Mohamed WI, Faty M, Petzold G, Beckwith RE, (2016). Cullin-RING ubiquitin E3 ligase regulation by the COP9 signalosome. Nature 531, 598–603. 27029275 10.1038/nature17416

[17] Baek K, Scott DC, Henneberg LT, King MT, Mann M, and Schulman BA (2023). Systemwide disassembly and assembly of SCF ubiquitin ligase complexes. Cell 186, 2492. 37236156 10.1016/j.cell.2023.04.036 PMC10228279

[18] Liu X, Reitsma JM, Mamrosh JL, Zhang Y, Straube R, and Deshaies RJ (2018). Cand1-Mediated Adaptive Exchange Mechanism Enables Variation in F-Box Protein Expression. Mol. Cell 69, 773–786.e6. 29499133 10.1016/j.molcel.2018.01.038 PMC5836512

[19] Harper JW, and Schulman BA (2021). Cullin-RING Ubiquitin Ligase Regulatory Circuits: A Quarter Century Beyond the F-Box Hypothesis. Annu. Rev. Biochem 90, 403–429. 33823649 10.1146/annurev-biochem-090120-013613 PMC8217159

[20] Skaar JR, Pagan JK, and Pagano M. (2013). Mechanisms and function of substrate recruitment by F-box proteins. Nat. Rev. Mol. Cell Biol 14, 369–381. 23657496 10.1038/nrm3582 PMC3827686

[21] George DJ., and Kaelin WG Jr. (2003). The von Hippel-Lindau protein, vascular endothelial growth factor, and kidney cancer. N. Engl. J. Med 349, 419–421. 12890838 10.1056/NEJMp030061

[22] Dubey AA, Krygier M, Szulc NA, Rutkowska K, Kosińska J, Pollak A, Rydzanicz M, Kmieć T, Mazurkiewicz-Bełdzińska M, Pokrzywa W, (2023). A novel de novo FEM1C variant is linked to neurodevelopmental disorder with absent speech, pyramidal signs and limb ataxia. Hum. Mol. Genet 32, 1152–1161. 36336956 10.1093/hmg/ddac276 PMC10026218

[23] Ivan M, Kondo K, Yang H, Kim W, Valiando J, Ohh M, Salic A, Asara JM, Lane WS, and Kaelin WG Jr. (2001). HIFalpha targeted for VHL-mediated destruction by proline hydroxylation: implications for O2 sensing. Science 292, 464–468. 11292862 10.1126/science.1059817

[24] Manford AG, Rodríguez-Pérez F, Shih KY, Shi Z, Berdan CA, Choe M, Titov DV, Nomura DK, and Rape M. (2020). A Cellular Mechanism to Detect and Alleviate Reductive Stress. Cell 183, 46–61.e21. 32941802 10.1016/j.cell.2020.08.034

[25] Manford AG, Mena EL, Shih KY, Gee CL, McMinimy R, Martínez-González B, Sherriff R, Lew B, Zoltek M, Rodríguez-Pérez F, (2021). Structural basis and regulation of the reductive stress response. Cell 184, 5375–5390.e16. 34562363 10.1016/j.cell.2021.09.002 PMC8810291

[26] Pickart CM, and Rose IA (1985). Functional heterogeneity of ubiquitin carrier proteins. J. Biol. Chem 260, 1573–1581. 2981864

[27] Schwob E, Böhm T, Mendenhall MD, and Nasmyth K. (1994). The B-type cyclin kinase inhibitor p40SIC1 controls the G1 to S transition in S. cerevisiae. Cell 79, 233–244. 7954792 10.1016/0092-8674(94)90193-7

[28] Verma R, Feldman RM, and Deshaies RJ (1997). SIC1 is ubiquitinated in vitro by a pathway that requires CDC4, CDC34, and cyclin/CDK activities. Mol. Biol. Cell 8, 1427–1437. 9285816 10.1091/mbc.8.8.1427 PMC276167

[29] Kelsall IR, Duda DM, Olszewski JL, Hofmann K, Knebel A, Langevin F, Wood N, Wightman M, Schulman BA, and Alpi AF (2013). TRIAD1 and HHARI bind to and are activated by distinct neddylated Cullin-RING ligase complexes. EMBO J. 32, 2848–2860. 24076655 10.1038/emboj.2013.209 PMC3817463

[30] Scott DC, Rhee DY, Duda DM, Kelsall IR, Olszewski JL, Paulo JA, de Jong A, Ovaa H, Alpi AF, Harper JW, and Schulman BA (2016). Two Distinct Types of E3 Ligases Work in Unison to Regulate Substrate Ubiquitylation. Cell 166, 1198–1214.e24. 27565346 10.1016/j.cell.2016.07.027 PMC5091668

[31] Dove KK, Kemp HA, Di Bona KR, Reiter KH, Milburn LJ, Camacho D, Fay DS, Miller DL, and Klevit RE (2017). Two functionally distinct E2/E3 pairs coordinate sequential ubiquitination of a common substrate in Caenorhabditis elegans development. Proc. Natl. Acad. Sci. USA 114, E6576–E6584. 28739890 10.1073/pnas.1705060114 PMC5559030

[32] Wu K, Kovacev J, and Pan ZQ (2010). Priming and extending: a UbcH5/Cdc34 E2 handoff mechanism for polyubiquitination on a SCF substrate. Mol. Cell 37, 784–796. 20347421 10.1016/j.molcel.2010.02.025 PMC2862584

[33] Saha A, and Deshaies RJ (2008). Multimodal activation of the ubiquitin ligase SCF by Nedd8 conjugation. Mol. Cell 32, 21–31. 18851830 10.1016/j.molcel.2008.08.021 PMC2644375

[34] Sievers QL, Gasser JA, Cowley GS, Fischer ES, and Ebert BL (2018). Genome-wide screen identifies cullin-RING ligase machinery required for lenalidomide-dependent CRL4(CRBN) activity. Blood 132, 1293–1303. 30042095 10.1182/blood-2018-01-821769 PMC6148446

[35] Lu G, Weng S, Matyskiela M, Zheng X, Fang W, Wood S, Surka C, Mizukoshi R, Lu CC, Mendy D, (2018). UBE2G1 governs the destruction of cereblon neomorphic substrates. eLife 7, e40958. 30234487 10.7554/eLife.40958 PMC6185104

[36] Gazdoiu S, Yamoah K, Wu K, and Pan Z-Q (2007). Human Cdc34 Employs Distinct Sites To Coordinate Attachment of Ubiquitin to a Substrate and Assembly of Polyubiquitin Chains. Mol. Cell. Biol 27, 7041–7052. 17698585 10.1128/MCB.00812-07 PMC2168909

[37] Hill S, Reichermeier K, Scott DC, Samentar L, Coulombe-Huntington J, Izzi L, Tang X, Ibarra R, Bertomeu T, Moradian A, (2019). Robust cullin-RING ligase function is established by a multiplicity of poly-ubiquitylation pathways. eLife 8, e51163. 31868589 10.7554/eLife.51163 PMC6975927

[38] Baek K, Krist DT, Prabu JR, Hill S, Klügel M, Neumaier LM, von Gronau S, Kleiger G, and Schulman BA (2020). NEDD8 nucleates a multivalent cullin-RING-UBE2D ubiquitin ligation assembly. Nature 578, 461–466. 32051583 10.1038/s41586-020-2000-y PMC7050210

[39] Horn-Ghetko D, Krist DT, Prabu JR, Baek K, Mulder MPC, Klügel M, Scott DC, Ovaa H, Kleiger G, and Schulman BA (2021). Ubiquitin ligation to F-box protein targets by SCF-RBR E3-E3 super-assembly. Nature 590, 671–676. 33536622 10.1038/s41586-021-03197-9 PMC7904520

[40] Huttenhain R, Xu J, Burton LA, Gordon DE, Hultquist JF, Johnson JR, Satkamp L, Hiatt J, Rhee DY, Baek K, (2019). ARIH2 Is a Vif-Dependent Regulator of CUL5-Mediated APOBEC3G Degradation in HIV Infection. Cell Host Microbe 26, 86–99.e87. 10.1016/j.chom.2019.05.008. 31253590 PMC7153695

[41] Feldman RM, Correll CC, Kaplan KB, and Deshaies RJ (1997). A complex of Cdc4p, Skp1p, and Cdc53p/cullin catalyzes ubiquitination of the phosphorylated CDK inhibitor Sic1p. Cell 91, 221–230. 9346239 10.1016/s0092-8674(00)80404-3

[42] Choi YS, Lee YJ, Lee SY, Shi L, Ha JH, Cheong HK, Cheong C, Cohen RE, and Ryu KS (2015). Differential ubiquitin binding by the acidic loops of Ube2g1 and Ube2r1 enzymes distinguishes their Lys-48-ubiquitylation activities. J. Biol. Chem 290, 2251–2263. 25471371 10.1074/jbc.M114.624809 PMC4303676

[43] Liwocha J, Krist DT, van der Heden van Noort GJ, Hansen FM, Truong VH, Karayel O, Purser N, Houston D, Burton N, Bostock MJ, (2021). Linkage-specific ubiquitin chain formation depends on a lysine hydrocarbon ruler. Nat. Chem. Biol 17, 272–279. 33288957 10.1038/s41589-020-00696-0 PMC7904580

[44] Thrower JS, Hoffman L, Rechsteiner M, and Pickart CM (2000). Recognition of the polyubiquitin proteolytic signal. EMBO J. 19, 94–102. 10619848 10.1093/emboj/19.1.94 PMC1171781

[45] Wickliffe KE, Lorenz S, Wemmer DE, Kuriyan J, and Rape M. (2011). The mechanism of linkage-specific ubiquitin chain elongation by a single-subunit E2. Cell 144, 769–781. 21376237 10.1016/j.cell.2011.01.035 PMC3072108

[46] Bremm A, Freund SM, and Komander D. (2010). Lys11-linked ubiquitin chains adopt compact conformations and are preferentially hydrolyzed by the deubiquitinase Cezanne. Nat. Struct. Mol. Biol 17, 939–947. 20622874 10.1038/nsmb.1873 PMC2917782

[47] Saha A, Lewis S, Kleiger G, Kuhlman B, and Deshaies RJ (2011). Essential role for ubiquitin-ubiquitin-conjugating enzyme interaction in ubiquitin discharge from Cdc34 to substrate. Mol. Cell 42, 75–83. 21474069 10.1016/j.molcel.2011.03.016 PMC3091889

[48] Wang GL, Jiang BH, Rue EA, and Semenza GL (1995). Hypoxia-inducible factor 1 is a basic-helix-loop-helix-PAS heterodimer regulated by cellular O2 tension. Proc. Natl. Acad. Sci. USA 92, 5510–5514. 7539918 10.1073/pnas.92.12.5510 PMC41725

[49] Jaakkola P, Mole DR, Tian YM, Wilson MI, Gielbert J, Gaskell SJ, von Kriegsheim A, Hebestreit HF, Mukherji M, Schofield CJ, (2001). Targeting of HIF-alpha to the von Hippel-Lindau ubiquitylation complex by O2-regulated prolyl hydroxylation. Science 292, 468–472. 11292861 10.1126/science.1059796

[50] Timms RT, Zhang Z, Rhee DY, Harper JW, Koren I, and Elledge SJ (2019). A glycine-specific N-degron pathway mediates the quality control of protein N-myristoylation. Science 365, eaaw4912. 10.1126/science.aaw4912 PMC7090375 31273098

[51] Koren I, Timms RT, Kula T, Xu Q, Li MZ, and Elledge SJ (2018). The Eukaryotic Proteome Is Shaped by E3 Ubiquitin Ligases Targeting C-Terminal Degrons. Cell 173, 1622–1635.e14. 29779948 10.1016/j.cell.2018.04.028 PMC6003881

[52] Raina K, Lu J, Qian Y, Altieri M, Gordon D, Rossi AM, Wang J, Chen X, Dong H, Siu K, (2016). PROTAC-induced BET protein degradation as a therapy for castration-resistant prostate cancer. Proc. Natl. Acad. Sci. USA 113, 7124–7129. 27274052 10.1073/pnas.1521738113 PMC4932933

[53] Winter GE, Buckley DL, Paulk J, Roberts JM, Souza A, Dhe-Paganon S, and Bradner JE (2015). DRUG DEVELOPMENT. Phthalimide conjugation as a strategy for in vivo target protein degradation. Science 348, 1376–1381. 25999370 10.1126/science.aab1433 PMC4937790

[54] Zengerle M, Chan KH, and Ciulli A. (2015). Selective Small Molecule Induced Degradation of the BET Bromodomain Protein BRD4. ACS Chem. Biol 10, 1770–1777. 10.1021/acschembio.5b00216 PMC4548256 26035625

[55] Henning NJ, Manford AG, Spradlin JN, Brittain SM, Zhang E, McKenna JM, Tallarico JA, Schirle M, Rape M, and Nomura DK (2022). Discovery of a Covalent FEM1B Recruiter for Targeted Protein Degradation Applications. J. Am. Chem. Soc 144, 701–708. 34994556 10.1021/jacs.1c03980 PMC8928484

[56] Kamura T., Maenaka K., Kotoshiba S., Matsumoto M., Kohda D., Conaway RC., Conaway JW., and Nakayama KI. (2004). VHL-box and SOCS-box domains determine binding specificity for Cul2-Rbx1 and Cul5-Rbx2 modules of ubiquitin ligases. Genes Dev. 18, 3055–3065. 15601820 10.1101/gad.1252404 PMC535916

[57] Mahrour N, Redwine WB, Florens L, Swanson SK, Martin-Brown S, Bradford WD, Staehling-Hampton K, Washburn MP, Conaway RC, and Conaway JW (2008). Characterization of Cullin-box sequences that direct recruitment of Cul2-Rbx1 and Cul5-Rbx2 modules to Elongin BC-based ubiquitin ligases. J. Biol. Chem 283, 8005–8013. 18187417 10.1074/jbc.M706987200

[58] Petroski MD, and Deshaies RJ (2005). Mechanism of lysine 48-linked ubiquitin-chain synthesis by the cullin-RING ubiquitin-ligase complex SCF-Cdc34. Cell 123, 1107–1120. 16360039 10.1016/j.cell.2005.09.033

[59] Williams KM, Qie S, Atkison JH, Salazar-Arango S, Alan Diehl J, and Olsen SK (2019). Structural insights into E1 recognition and the ubiquitin-conjugating activity of the E2 enzyme Cdc34. Nat. Commun 10, 3296. 31341161 10.1038/s41467-019-11061-8 PMC6656757

[60] Pierce NW, Kleiger G, Shan SO, and Deshaies RJ (2009). Detection of sequential polyubiquitylation on a millisecond timescale. Nature 462, 615–619. 19956254 10.1038/nature08595 PMC2791906

[61] S1abicki M, Yoon H, Koeppel J, Nitsch L, Roy Burman SS, Di Genua C, Donovan KA, Sperling AS, Hunkeler M, Tsai JM, (2020). Small-molecule-induced polymerization triggers degradation of BCL6. Nature 588, 164–168. 33208943 10.1038/s41586-020-2925-1 PMC7816212

[62] Wu K, Huynh KQ, Lu I, Moustakim M, Miao H, Yu C, Haeusgen MJ, Hopkins BD, Huang L, Zheng N, (2021). Inhibitors of cullin-RING E3 ubiquitin ligase 4 with antitumor potential. Proc. Natl. Acad. Sci. USA 118, e2007328118. 33602808 10.1073/pnas.2007328118 PMC7923628

[63] Zhou P, and Howley PM (1998). Ubiquitination and degradation of the substrate recognition subunits of SCF ubiquitin-protein ligases. Mol. Cell 2, 571–580. 9844630 10.1016/s1097-2765(00)80156-2

[64] Henneberg LT, Singh J, Duda DM, Baek K, Yanishevski D, Murray PJ, Mann M, Sidhu SS, and Schulman BA (2023). Activity-based profiling of cullin-RING ligase networks by conformation-specific probes. Nat. Chem. Biol 19, 1513–1523. 37653169 10.1038/s41589-023-01392-5 PMC10667097

[65] Hanzl A, Barone E, Bauer S, Yue H, Nowak RP, Hahn E, Pankevich EV, Koren A, Kubicek S, Fischer ES, and Winter GE (2023). E3-Specific Degrader Discovery by Dynamic Tracing of Substrate Receptor Abundance. J. Am. Chem. Soc 145, 1176–1184. 36602777 10.1021/jacs.2c10784 PMC9853857

[66] Zhang Y, Jost M, Pak RA, Lu D, Li J, Lomenick B, Garbis SD, Li CM, Weissman JS, Lipford JR, and Deshaies RJ (2022). Adaptive exchange sustains cullin-RING ubiquitin ligase networks and proper licensing of DNA replication. Proc. Natl. Acad. Sci. USA 119, e2205608119. 36037385 10.1073/pnas.2205608119 PMC9456757

[67] Kleiger G, Saha A, Lewis S, Kuhlman B, and Deshaies RJ (2009). Rapid E2-E3 assembly and disassembly enable processive ubiquitylation of cullin-RING ubiquitin ligase substrates. Cell 139, 957–968. 19945379 10.1016/j.cell.2009.10.030 PMC2804849

[68] Zheng N, Schulman BA, Song L, Miller JJ, Jeffrey PD, Wang P, Chu C, Koepp DM, Elledge SJ, Pagano M, (2002). Structure of the Cul1-Rbx1-Skp1-F boxSkp2 SCF ubiquitin ligase complex. Nature 416, 703–709. 11961546 10.1038/416703a

[69] Nakasone MA, Majorek KA, Gabrielsen M, Sibbet GJ, Smith BO, and Huang DT (2022). Structure of UBE2K-Ub/E3/polyUb reveals mechanisms of K48-linked Ub chain extension. Nat. Chem. Biol 18, 422–431. 35027744 10.1038/s41589-021-00952-x PMC8964413

[70] Middleton AJ, and Day CL (2015). The molecular basis of lysine 48 ubiquitin chain synthesis by Ube2K. Sci. Rep 5, 16793. 26592444 10.1038/srep16793 PMC4655369

[71] Brown NG, VanderLinden R, Watson ER, Weissmann F, Ordureau A, Wu KP, Zhang W, Yu S, Mercredi PY, Harrison JS, (2016). Dual RING E3 Architectures Regulate Multiubiquitination and Ubiquitin Chain Elongation by APC/C. Cell 165, 1440–1453. 27259151 10.1016/j.cell.2016.05.037 PMC4991212

[72] Welsh KA, Bolhuis DL, Nederstigt AE, Boyer J, Temple BRS, Bonacci T, Gu L, Ordureau A, Harper JW, Steimel JP, (2022). Functional conservation and divergence of the helix-turn-helix motif of E2 ubiquitin-conjugating enzymes. EMBO J. 41, e108823. 34942047 10.15252/embj.2021108823 PMC8804933

[73] Steigenberger B, Pieters RJ, Heck AJR, and Scheltema RA (2019). PhoX: An IMAC-Enrichable Cross-Linking Reagent. ACS Cent. Sci 5, 1514–1522. 31572778 10.1021/acscentsci.9b00416 PMC6764163

[74] Gadd MS, Testa A, Lucas X, Chan KH, Chen W, Lamont DJ, Zengerle M, and Ciulli A. (2017). Structural basis of PROTAC cooperative recognition for selective protein degradation. Nat. Chem. Biol 13, 514–521. 28288108 10.1038/nchembio.2329 PMC5392356

[75] Mathias N, Steussy CN, and Goebl MG (1998). An essential domain within Cdc34p is required for binding to a complex containing Cdc4p and Cdc53p in Saccharomyces cerevisiae. J. Biol. Chem 273, 4040–4045. 9461595 10.1074/jbc.273.7.4040

[76] Sadowski M, Mawson A, Baker R, and Sarcevic B. (2007). Cdc34 C-terminal tail phosphorylation regulates Skp1/cullin/F-box (SCF)-mediated ubiquitination and cell cycle progression. Biochem. J 405, 569–581. 17461777 10.1042/BJ20061812 PMC2267305

[77] Choi YS, Wu K, Jeong K, Lee D, Jeon YH, Choi BS, Pan ZQ, Ryu KS, and Cheong C. (2010). The human Cdc34 carboxyl terminus contains a non-covalent ubiquitin binding activity that contributes to SCF-dependent ubiquitination. J. Biol. Chem 285, 17754–17762. 20353940 10.1074/jbc.M109.090621 PMC2878539

[78] Silver ET, Gwozd TJ, Ptak C, Goebl M, and Ellison MJ (1992). A chimeric ubiquitin conjugating enzyme that combines the cell cycle properties of CDC34 (UBC3) and the DNA repair properties of RAD6 (UBC2): implications for the structure, function and evolution of the E2s. EMBO J. 11, 3091–3098. 1639076 10.1002/j.1460-2075.1992.tb05381.x PMC556793

[79] Kleiger G, Hao B, Mohl DA, and Deshaies RJ (2009). The acidic tail of the Cdc34 ubiquitin-conjugating enzyme functions in both binding to and catalysis with ubiquitin ligase SCFCdc4. J. Biol. Chem 284, 36012–36023. 19875449 10.1074/jbc.M109.058529 PMC2794717

[80] Sandoval D, Hill S, Ziemba A, Lewis S, Kuhlman B, and Kleiger G. (2015). Ubiquitin-conjugating Enzyme Cdc34 and Ubiquitin Ligase Skp1-Cullin-F-box Ligase (SCF) Interact through Multiple Conformations. J. Biol. Chem 290, 1106–1118. 25425648 10.1074/jbc.M114.615559 PMC4294478

[81] Liwocha J, Li J, Purser N, Rattanasopa C, Maiwald S, Krist DT, Scott DC, Steigenberger B, Prabu JR, Schulman BA, and Kleiger G. (2024). Mechanism of millisecond Lys48-linked poly-ubiquitin chain formation by cullin-RING ligases. Nat Struct Mol Biol. 10.1038/s41594-023-01206-1. PMC10873206 38326650

[82] Bondeson DP, Smith BE, Burslem GM, Buhimschi AD, Hines J, Jaime-Figueroa S, Wang J, Hamman BD, Ishchenko A, and Crews CM (2018). Lessons in PROTAC Design from Selective Degradation with a Promiscuous Warhead. Cell Chem. Biol 25, 78–87.e5. 29129718 10.1016/j.chembiol.2017.09.010 PMC5777153

[83] Nowak RP, DeAngelo SL, Buckley D, He Z, Donovan KA, An J, Safaee N, Jedrychowski MP, Ponthier CM, Ishoey M, (2018). Plasticity in binding confers selectivity in ligand-induced protein degradation. Nat. Chem. Biol 14, 706–714. 29892083 10.1038/s41589-018-0055-y PMC6202246

[84] Lai AC, and Crews CM (2017). Induced protein degradation: an emerging drug discovery paradigm. Nat. Rev. Drug Discov 16, 101–114. 27885283 10.1038/nrd.2016.211 PMC5684876

[85] Jan M, Sperling AS, and Ebert BL (2021). Cancer therapies based on targeted protein degradation - lessons learned with lenalidomide. Nat. Rev. Clin. Oncol 18, 401–417. 33654306 10.1038/s41571-021-00479-z PMC8903027

[86] Békés M, Langley DR, and Crews CM (2022). PROTAC targeted protein degraders: the past is prologue. Nat. Rev. Drug Discov 21, 181–200. 35042991 10.1038/s41573-021-00371-6 PMC8765495

[87] Mayor-Ruiz C, Jaeger MG, Bauer S, Brand M, Sin C, Hanzl A, Mueller AC, Menche J, and Winter GE (2019). Plasticity of the Cullin-RING Ligase Repertoire Shapes Sensitivity to Ligand-Induced Protein Degradation. Mol. Cell 75, 849–858.e8. 31442425 10.1016/j.molcel.2019.07.013

[88] Scott DC, Sviderskiy VO, Monda JK, Lydeard JR, Cho SE, Harper JW, and Schulman BA (2014). Structure of a RING E3 Trapped in Action Reveals Ligation Mechanism for the Ubiquitin-like Protein NEDD8. Cell 157, 1671–1684. 24949976 10.1016/j.cell.2014.04.037 PMC4247792

[89] Kostrhon S, Prabu JR, Baek K, Horn-Ghetko D, von Gronau S, Klugel M, Basquin J, Alpi AF, and Schulman BA (2021). CUL5-ARIH2 E3-E3 ubiquitin ligase structure reveals cullin-specific NEDD8 activation. Nat Chem Biol 17, 1075–1083. 10.1038/s41589-021-00858-8. 34518685 PMC8460447

[90] Ziemba A, Hill S, Sandoval D, Webb K, Bennett EJ, and Kleiger G. (2013). Multimodal mechanism of action for the Cdc34 acidic loop: a case study for why ubiquitin-conjugating enzymes have loops and tails. J. Biol. Chem 288, 34882–34896. 24129577 10.1074/jbc.M113.509190 PMC3843100

[91] Cong L, Ran FA, Cox D, Lin S, Barretto R, Habib N, Hsu PD, Wu X, Jiang W, Marraffini LA, and Zhang F. (2013). Multiplex genome engineering using CRISPR/Cas systems. Science 339, 819–823. 23287718 10.1126/science.1231143 PMC3795411

[92] Weissmann F, Petzold G, VanderLinden R, Huis In ‘t Veld PJ, Brown NG, Lampert F, Westermann S, Stark H, Schulman BA, and Peters JM (2016). biGBac enables rapid gene assembly for the expression of large multisubunit protein complexes. Proc. Natl. Acad. Sci. USA 113, E2564–E2569. 27114506 10.1073/pnas.1604935113 PMC4868461

[93] Scott DC, King MT, Baek K, Gee CT, Kalathur R, Li J, Purser N, Nourse A, Chai SC, Vaithiyalingam S, (2023). E3 ligase autoinhibition by C-degron mimicry maintains C-degron substrate fidelity. Mol. Cell 83, 770–786.e9. 36805027 10.1016/j.molcel.2023.01.019 PMC10080726

[94] Diaz S, Li L, Wang K, and Liu X. (2021). Expression and purification of functional recombinant CUL2*RBX1 from E. coli. Sci. Rep 11, 11224. 34045610 10.1038/s41598-021-90770-x PMC8160325

[95] Punjani A, Rubinstein JL, Fleet DJ, and Brubaker MA (2017). cryoSPARC: algorithms for rapid unsupervised cryo-EM structure determination. Nat. Methods 14, 290–296. 28165473 10.1038/nmeth.4169

[96] Zivanov J, Nakane T, Forsberg BO, Kimanius D, Hagen WJ, Lindahl E, and Scheres SH (2018). New tools for automated high-resolution cryo-EM structure determination in RELION-3. eLife 7, e42166. 30412051 10.7554/eLife.42166 PMC6250425

[97] Zheng SQ, Palovcak E, Armache JP, Verba KA, Cheng Y, and Agard DA (2017). MotionCor2: anisotropic correction of beam-induced motion for improved cryo-electron microscopy. Nat. Methods 14, 331–332. 28250466 10.1038/nmeth.4193 PMC5494038

[98] Pettersen EF, Goddard TD, Huang CC, Couch GS, Greenblatt DM, Meng EC, and Ferrin TE (2004). UCSF Chimera–a visualization system for exploratory research and analysis. J. Comput. Chem 25, 1605–1612. 15264254 10.1002/jcc.20084

[99] Rohou A, and Grigorieff N. (2015). CTFFIND4: Fast and accurate defocus estimation from electron micrographs. J. Struct. Biol 192, 216–221. 26278980 10.1016/j.jsb.2015.08.008 PMC6760662

[100] Zhang K. (2016). Gctf: Real-time CTF determination and correction. J. Struct. Biol 193, 1–12. 26592709 10.1016/j.jsb.2015.11.003 PMC4711343

[101] Sanchez-Garcia R, Gomez-Blanco J, Cuervo A, Carazo JM, Sorzano COS, and Vargas J. (2021). DeepEMhancer: a deep learning solution for cryo-EM volume post-processing. Commun. Biol 4, 874. 34267316 10.1038/s42003-021-02399-1 PMC8282847

[102] Biyani N, Righetto RD, McLeod R, Caujolle-Bert D, Castano-Diez D, Goldie KN, and Stahlberg H. (2017). Focus: The interface between data collection and data processing in cryo-EM. J. Struct. Biol 198, 124–133. 28344036 10.1016/j.jsb.2017.03.007

[103] Goddard TD, Huang CC, Meng EC, Pettersen EF, Couch GS, Morris JH, and Ferrin TE (2018). UCSF ChimeraX: Meeting modern challenges in visualization and analysis. Protein Sci. 27, 14–25. 28710774 10.1002/pro.3235 PMC5734306

[104] Emsley P, Lohkamp B, Scott WG, and Cowtan K. (2010). Features and development of Coot. Acta Crystallogr. D Biol. Crystallogr 66, 486–501. 20383002 10.1107/S0907444910007493 PMC2852313

[105] Afonine PV, Klaholz BP, Moriarty NW, Poon BK, Sobolev OV, Terwilliger TC, Adams PD, and Urzhumtsev A. (2018). New tools for the analysis and validation of cryo-EM maps and atomic models. Acta Crystallogr. D Struct. Biol 74, 814–840. 30198894 10.1107/S2059798318009324 PMC6130467

[106] Chen X, Liao S, Makaros Y, Guo Q, Zhu Z, Krizelman R, Dahan K, Tu X, Yao X, Koren I, (2021). Molecular basis for arginine C-terminal degron recognition by Cul2(FEM1) E3 ligase. Nat. Chem. Biol 17, 254–262. 33398168 10.1038/s41589-020-00704-3

[107] Purser N, Tripathi-Giesgen I, Li J, Scott DC, Horn-Ghetko D, Baek K, Schulman BA, Alpi AF, and Kleiger G. (2023). Catalysis of non-canonical protein ubiquitylation by the ARIH1 ubiquitin ligase. Biochem. J 480, 1817–1831. 37870100 10.1042/BCJ20230373 PMC10657180

[108] Paulo JA, and Gygi SP (2017). Nicotine-induced protein expression profiling reveals mutually altered proteins across four human cell lines. Proteomics 17. 10.1002/pmic.201600319 PMC5540149 27862958

[109] Rappsilber J, Ishihama Y, and Mann M. (2003). Stop and go extraction tips for matrix-assisted laser desorption/ionization, nanoelectrospray, and LC/MS sample pretreatment in proteomics. Anal. Chem 75, 663–670. 12585499 10.1021/ac026117i

[110] Swatek KN, Usher JL, Kueck AF, Gladkova C, Mevissen TET, Pruneda JN, Skern T, and Komander D. (2019). Insights into ubiquitin chain architecture using Ub-clipping. Nature 572, 533–537. 31413367 10.1038/s41586-019-1482-y PMC6823057

[111] Jumper J, Evans R, Pritzel A, Green T, Figurnov M, Ronneberger O, Tunyasuvunakool K, Bates R, Ž´ıdek A, Potapenko A, (2021). Highly accurate protein structure prediction with AlphaFold. Nature 596, 583–589. 34265844 10.1038/s41586-021-03819-2 PMC8371605

[112] Klykov O, Steigenberger B, Pektaş S, Fasci D, Heck AJR, and Scheltema RA (2018). Efficient and robust proteome-wide approaches for cross-linking mass spectrometry. Nat. Protoc 13, 2964–2990. 30446747 10.1038/s41596-018-0074-x

