# Supplementary material for: Cullin-RING ligases employ geometrically optimized catalytic partners for substrate targeting
Source: Mol Cell. Author manuscript; Available in PMC 2024 Apr 6. (PMC10997478; doi:10.1016/j.molcel.2024.01.022)
Supplement: MMC1 [file NIHMS1965369-supplement-MMC1.pdf]

Molecular Cell, Volume *84*

Supplemental information

Cullin-RING ligases employ geometrically optimized catalytic partners for substrate targeting

Jerry Li, Nicholas Purser, Joanna Liwocha, Daniel C. Scott, Holly A. Byers, Barbara Steigenberger, Spencer Hill, Ishita Tripathi-Giesgen, Trent Hinkle, Fynn M. Hansen, J. Rajan Prabu, Senthil K. Radhakrishnan, Donald S. Kirkpatrick, Kurt M. Reichermeier, Brenda A. Schulman, and Gary Kleiger

Table S1. Estimates of K_m and k_{obs} for CRL-substrate priming, Related to Figures 1, 5, 6, S1, S5, and S7.

Substrate[a]	CRL[b]	UCE	UCE Mutant	K_m[c] (10^{-9} M)	$k_{obs}^{S0\text{-}S1}$ (sec^{-1})	$k_{obs}^{S0\text{-}S1}/K_m$ ($M^{-1}sec^{-1}$)
Hif1α	CRL2^{VHL}	UBE2R2	WT	316 ± 57	0.575 ± 0.006	1.82E6
Hif1α	CRL2^{VHL}	ARIH1	WT	151 ± 29	[d]0.367 ± 0.010	2.43E6
Hif1α	CRL2^{VHL}	UBE2D3	WT	6240 ± 1132	0.0423 ± 0.003	6.78E3
Sil1	CRL2^{FEM1C}	UBE2R2	WT	65 ± 14	2.80 ± 0.227	4.31E7
Sil1	CRL2^{FEM1C}	ARIH1	WT	143 ± 29	0.0734 ± 0.003	5.13E5
Sil1	CRL2^{FEM1C}	UBE2D3	WT	916 ± 243	0.438 ± 0.022	4.78E5
Sil1	CRL2^{FEM1C}	UBE2G1	WT	1137 ± 295	0.146 ± 0.009	1.28E5
BRD4^{BD2}	CRL2$^{VHL\text{-}ARV\text{-}771}$	UBE2R2	WT	418 ± 80	0.398 ± 0.033	9.52E5
BRD4^{BD2}	CRL2$^{VHL\text{-}MZ1}$	UBE2R2	WT	387 ± 71	1.60 ± 0.071	4.13E6
BRD4^{BD2}	CRL2$^{VHL\text{-}ARV\text{-}771}$	ARIH1	WT	315 ± 67	0.107 ± 0.005	3.40E5
BRD4^{BD2}	CRL2$^{VHL\text{-}MZ1}$	ARIH1	WT	262 ± 50	0.070 ± 0.006	2.67E5
K368R BRD4^{BD2}	CRL2$^{VHL\text{-}ARV\text{-}771}$	UBE2R2	WT	-	0.051 ± 0.002	-
K368R BRD4^{BD2}	CRL2$^{VHL\text{-}MZ1}$	UBE2R2	WT	-	0.067 ± 0.001	-
K368R BRD4^{BD2}	CRL2$^{VHL\text{-}ARV\text{-}771}$	ARIH1	WT	-	0.149 ± 0.014	-
K368R BRD4^{BD2}	CRL2$^{VHL\text{-}MZ1}$	ARIH1	WT	-	0.051 ± 0.003	-
Sil1	CRL2^{FEM1C}	UBE2R2	E88R	2185 ± 501	0.335 ± 0.017	1.53E5
Sil1	CRL2^{FEM1C}	UBE2R2	V146A	395 ± 96	0.421 ± 0.010	1.07E6
Sil1	CRL2$^{D261R\ FEM1C}$	UBE2R2	WT	900 ± 214	0.113 ± 0.002	1.26E5
Sil1	CRL2$^{S351R\ FEM1C}$	UBE2R2	WT	479 ± 111	0.146 ± 0.006	3.05E5
Sil1	CRL2$^{D314\text{-}P315A\ FEM1C}$	UBE2R2	WT	462 ± 119	1.46 ± 0.039	3.16E6
BRD4^{BD2}	CRL2$^{VHL\text{-}MZ1}$	UBE2R2	Δ198-203	581 ± 110	0.482 ± 0.018	8.30E5
BRD4^{BD2}	CRL2$^{VHL\text{-}MZ1}$	UBE2R2	4G	1850 ± 285	0.188 ± 0.008	1.02E5
**Substrate**	**CRL**	**UCE**	**UCE Mutant**	$k_{obs}^{S0\text{-}S1}$(sec^{-1})	$k_{obs}^{S1\text{-}S2}$ (sec^{-1})	
Hif1α	CRL2^{VHL}	UBE2R2	WT	0.420[e] ± 0.013	27.54[e] ± 0.711	

UCE, ubiquitin-carrying enzyme; [a]Substrates are peptides except BRD4^{BD2} which is a protein; [b]All CRL complexes were covalently conjugated to the protein NEDD8; [c]K_m of the UCE for the CRL complex; [d]Purser et al, 2023 [S1]; [e]Experiments were performed with wild-type ubiquitin to enable poly-ubiquitin chain formation (see methods); Δ198-203 UBE2R2 is an internal deletion of residues 198-203; '4G' UBE2R2 is a quadruple point mutant (L206G Y207G L210G Y211G); BRD4^{BD2} encompasses residues 346-460 in the wild-type protein. All K_m and $k_{obs}^{S0\text{-}S1}$ values represent the mean from triplicate technical replicates with the standard error of measurement shown.

Table S2. Estimates of the equilibrium dissociation constant (K_d) for UBE2R2 and CRL2 complexes, Related to Figures 4 and S3.

UCE	[NaCl] (mM)	CRL	K_d (10^{-9} M)	95% CI* (10^{-9} M)	R^2
UBE2R2	50	NEDD8-CUL2-RBX1	276 ± 41	204 - 377	0.958
UBE2R2	100	NEDD8-CUL2-RBX1	1145 ± 183	840 – 1581	0.959
UBE2R2	50	NEDD8-$CRL2^{FEM1C}$	44 ± 7	32.2 - 60.2	0.882
UBE2R2	100	NEDD8-$CRL2^{FEM1C}$	156 ± 36	99.0 - 246	0.895
UBE2R2-UB	50	NEDD8-$CRL2^{FEM1C}$	50 ± 8	35.6 - 69.7	0.924

UCE, ubiquitin-carrying enzyme; *CI, confidence interval

Table S3. Reactions concentrations for CRL2-mediated ubiquitylation reactions, related to STAR Methods

Final concentrations for $CRL2^{VHL}$ Ubiquitylation Reactions | UBE2R2

Experiment	[E1] (μM)	[K48R UB] (μM)	[UBE2R2] (μM)	[CRL2] (μM)	[Substrate] (μM)
K_m	0.25	20	10*	0.25	0.1
QF	0.25	20	10	0.25	0.1

Final Concentrations for $CRL2^{VHL}$ Ubiquitylation Reactions | UBE2D3

Experiment	[E1] (μM)	[K0 UB] (μM)	[UBE2D3] (μM)	[CRL2] (μM)	[Substrate] (μM)
K_m	0.25	80	40*	0.25	0.1
QF	0.25	45	30	0.25	0.1

Final Concentrations for $CRL2^{VHL}$ Ubiquitylation Reactions | ARIH1

Experiment	[E1] (μM)	[K0 UB] (μM)	[UBE2L3] (μM)	[ARIH1] (μM)	[CRL2] (μM)	[Substrate] (μM)
K_m	0.25	10	7.5	6.6*	0.25	0.1
QF	0.5	6.25	5	2.5	0.25	0.1

Final concentrations for $CRL2^{FEM1C}$ Ubiquitylation Reactions | UBE2R2

Experiment	[E1] (μM)	[K48R UB] (μM)	[UBE2R2] (μM)	[CRL2] (μM)	[Substrate] (μM)
K_m	0.25	2	1*	0.25	0.25
QF	0.25	2	1	0.25	0.1

Final Concentrations for $CRL2^{FEM1C}$ Ubiquitylation Reactions | UBE2D3

Experiment	[E1] (μM)	[K0 UB] (μM)	[UBE2D3] (μM)	[CRL2] (μM)	[Substrate] (μM)
K_m	0.25	20	5*	0.25	0.1
QF	0.25	20	10	0.25	0.1

Final Concentrations for $CRL2^{FEM1C}$ Ubiquitylation Reactions | UBE2G1

Experiment	[E1] (μM)	[K0 UB] (μM)	[UBE2D3] (μM)	[CRL2] (μM)	[Substrate] (μM)
K_m	0.25	20	5*	0.25	0.1
QF	0.25	20	10	0.25	0.1

Final Concentrations for $CRL2^{FEM1C}$ Ubiquitylation Reactions | ARIH1

Experiment	[E1] (μM)	[K0 UB] (μM)	[UBE2L3] (μM)	[ARIH1] (μM)	[CRL2] (μM)	[Substrate] (μM)
K_m	0.25	2.5	2	1*	0.25	0.25
QF	0.25	2.5	2	1	0.25	0.1

Final Concentrations for $CRL2^{VHL}$ PROTAC Ubiquitylation Reactions | UBE2R2

Experiment	[E1] (μM)	[K48R UB] (μM)	[UBE2R2] (μM)	[CRL2] (μM)	[Substrate] (μM)	[PROTAC] (μM)
K_m	0.25	7.5	17.7*	0.25	0.25	2
QF	0.25	7.5	5	0.25	0.25	2

Final Concentrations for $CRL2^{VHL}$ PROTAC Ubiquitylation Reactions | ARIH1

	[E1] (μM)	[K0 UB] (μM)	[UBE2L3] (μM)	[ARIH1] (μM)	[CRL2] (μM)	[Substrate] (μM)	[PROTAC] (μM)
K_m	0.5	18.75	15	7.8*	0.25	0.25	2
QF	0.5	6.25	5	2.5	0.25	0.25	2

Final Concentrations for Mutant $CRL2^{FEM1C}$ Ubiquitylation Reactions | UBE2R2

Experiment	FEM1C Mutant	[E1] (μM)	[K48R UB] (μM)	[UBE2R2] (μM)	[CRL2] (μM)	[Substrate] (μM)
K_m	D261R	0.25	12.5	4*	0.25	0.25
QF	D261R	0.25	20	10	0.25	0.1
K_m	S351R	0.25	12.5	5	0.25	0.25
QF	S351R	0.25	20	10	0.25	0.1
K_m	D314A/P315A	0.25	12.5	5	0.25	0.25
QF	D314A/P315A	0.25	20	10	0.25	0.1

Final Concentrations for $CRL2^{FEM1C}$ Ubiquitylation Reactions | Mutant UBE2R2

Experiment	UBE2R2 Mutant	[E1] (μM)	[K48R UB] (μM)	[UBE2R2] (μM)	[CRL2] (μM)	[Substrate] (μM)
K_m	E88R	0.25	20	10*	0.25	0.25
QF	E88R	0.25	20	10	0.25	0.1
K_m	V146A	0.25	12.5	5	0.25	0.25
QF	V146A	0.25	20	10	0.25	0.1

Final Concentrations for $CRL2^{VHL-MZ1}$ PROTAC Ubiquitylation Reactions | Mutant UBE2R2

Experiment	UBE2R2 Mutant	[E1] (μM)	[K48R UB] (μM)	[UBE2R2] (μM)	[CRL2] (μM)	[Substrate] (μM)	[MZ1] (μM)
K_m	Δ198-203	0.25	10	5*	0.25	0.25	2
QF	Δ198-203	0.25	10	5	0.25	0.25	2
K_m	4G	0.25	10	5	0.25	0.25	2
QF	4G	0.25	10	5	0.25	0.25	2

*The top concentration of a 2-fold dilution series. QF; Quench flow. All quench flow and K_m reactions contained a final unlabeled peptide concentration of 10 μM, except for experiments involving the Sil1 peptide and $BRD4^{BD2}$ (346-460).

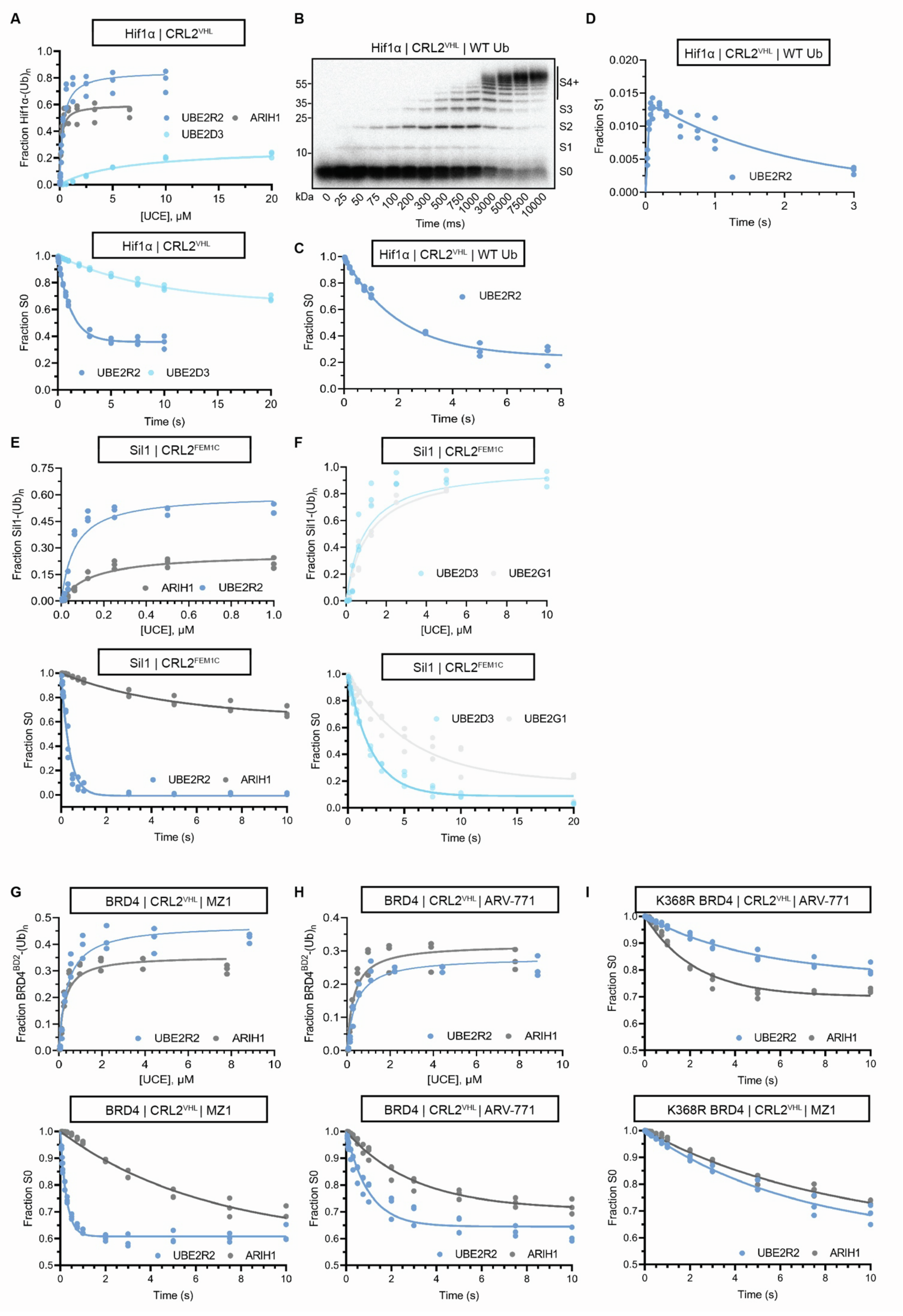
A
Hif1α | CRL2VHL
Fraction Hif1α-(Ub)n
UBE2R2
ARIH1
UBE2D3
[UCE], μM
Hif1α | CRL2VHL
Fraction S0
UBE2R2
UBE2D3
Time (s)
B
Hif1α | CRL2VHL | WT Ub
kDa
55
35
25
10
S4+
S3
S2
S1
S0
0 25 50 75 100 200 300 500 750 1000 3000 5000 7500 10000
Time (ms)
C
Hif1α | CRL2VHL | WT Ub
Fraction S0
UBE2R2
Time (s)
D
Hif1α | CRL2VHL | WT Ub
Fraction S1
UBE2R2
Time (s)
E
Sil1 | CRL2FEM1C
Fraction Sil1-(Ub)n
ARIH1
UBE2R2
[UCE], μM
Sil1 | CRL2FEM1C
Fraction S0
UBE2R2
ARIH1
Time (s)
F
Sil1 | CRL2FEM1C
Fraction Sil1-(Ub)n
UBE2D3
UBE2G1
[UCE], μM
Sil1 | CRL2FEM1C
Fraction S0
UBE2D3
UBE2G1
Time (s)
G
BRD4 | CRL2VHL | MZ1
Fraction BRD4BD2-(Ub)n
UBE2R2
ARIH1
[UCE], μM
BRD4 | CRL2VHL | MZ1
Fraction S0
UBE2R2
ARIH1
Time (s)
H
BRD4 | CRL2VHL | ARV-771
Fraction BRD4BD2-(Ub)n
UBE2R2
ARIH1
[UCE], μM
BRD4 | CRL2VHL | ARV-771
Fraction S0
UBE2R2
ARIH1
Time (s)
I
K368R BRD4 | CRL2VHL | ARV-771
Fraction S0
UBE2R2
ARIH1
Time (s)
K368R BRD4 | CRL2VHL | MZ1
Fraction S0
UBE2R2
ARIH1
Time (s)

Figure S1 – Estimation of the K_m and k_{obs} for various ubiquitin-carrying enzymes, CRL2s and substrates (related to Figure 1, Table S1, and STAR methods)

(A) The top graph shows the relationship between product formation and various ubiquitin-carrying enzyme (UCE) levels with neddylated CRL2^{VHL} and Hif1α peptide substrate. The data were fit to the Michaelis-Menten model using non-linear regression (GraphPad Prism software v10). The bottom graph shows the time course for Hif1α peptide substrate (S0) depletion in the presence of the indicated UCEs. The data were fit to previously described closed form solutions [S2] to estimate the rates of ubiquitin transfer (Mathematica v12).

(B) Autoradiogram showing product formation from pre-steady state ubiquitylation reactions with neddylated CRL2^{VHL}, UBE2R2, and radiolabeled Hif1α peptide substrate (see STAR methods). While ubiquitin mutants were typically employed to focus on substrate priming and to suppress poly-ubiquitin chain formation, here wild-type (WT) ubiquitin was used to assess UBE2R2's chain forming activity with a CUL2-based CRL. S0 represents unmodified Hif1α peptide, S1 represents ubiquitin-primed Hif1α peptide, and S2 is Hif1α peptide with a di-ubiquitin chain.

(C) Graph showing depletion of S0 substrate as a function of time for the reactions shown in (B) and the fit of the data to the model.

(D) Same as (C) except showing S1 product levels.

(E) Same as (A) but with neddylated CRL2^{FEM1C} and Sil1 peptide substrate.

(F) Same as (E) but with UBE2D3 and UBE2G1.

(G) Same as (A) but with recombinant BRD4 (346-460) and the PROTAC MZ1.

(H) Same as (G) but with the PROTAC ARV-771.

(I) Same as the graph for k_{obs} in (G) but with recombinant K368R BRD4 (346-460) and ARV-771 (top) or MZ1 (bottom).

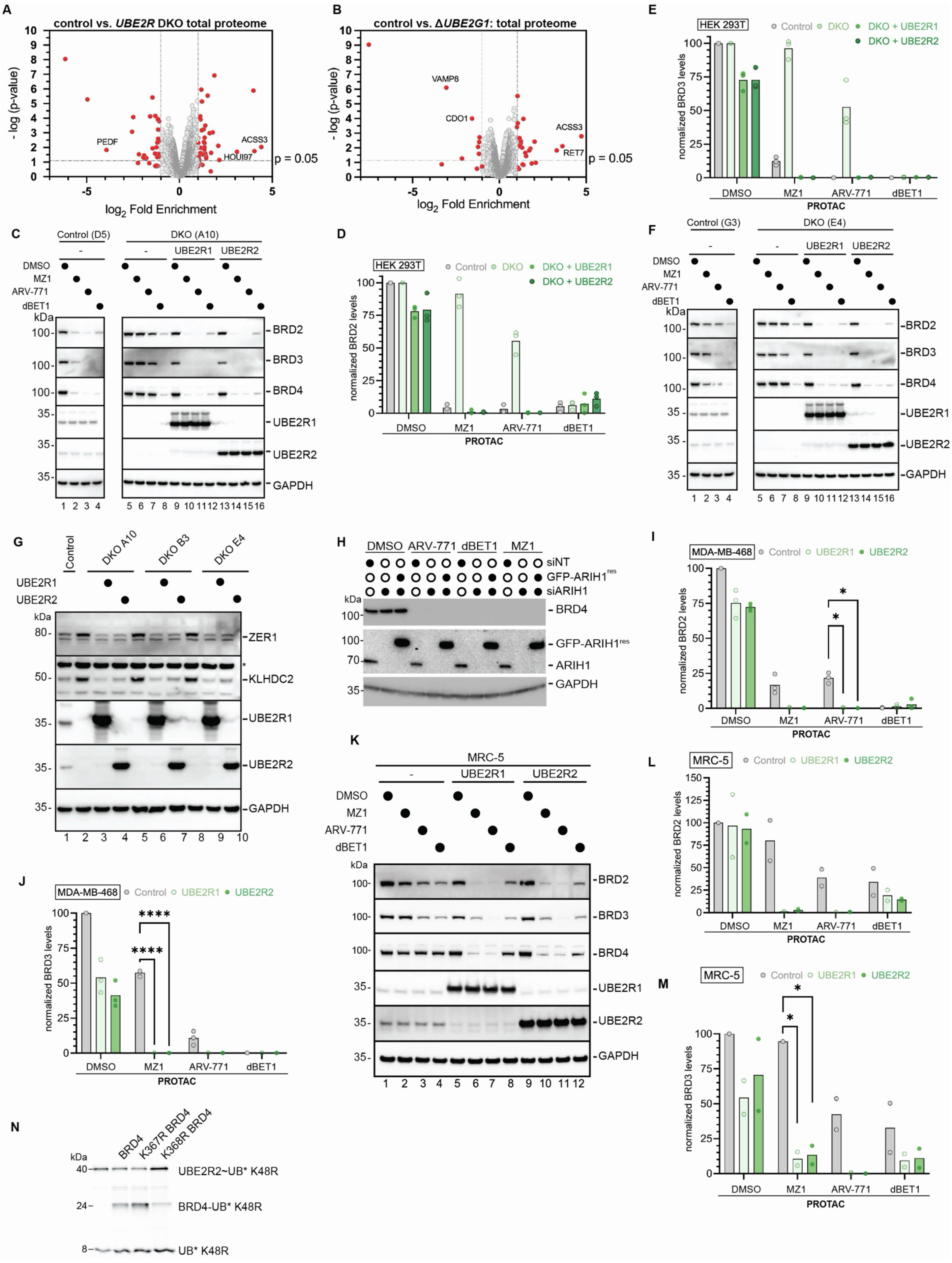
A
control vs. UBE2R DKO total proteome
- log (p-value)
log2 Fold Enrichment
PEDF
ACSS3
HOUI97
p = 0.05
B
control vs. ΔUBE2G1: total proteome
VAMP8
CDO1
ACSS3
RET7
p = 0.05
C
Control (D5)
DKO (A10)
UBE2R1
UBE2R2
DMSO
MZ1
ARV-771
dBET1
kDa
BRD2
BRD3
BRD4
UBE2R1
UBE2R2
GAPDH
1 2 3 4 5 6 7 8 9 10 11 12 13 14 15 16
D
HEK 293T
Control
DKO
DKO + UBE2R1
DKO + UBE2R2
normalized BRD2 levels
DMSO
MZ1
ARV-771
dBET1
PROTAC
E
HEK 293T
Control
DKO
DKO + UBE2R1
DKO + UBE2R2
normalized BRD3 levels
DMSO
MZ1
ARV-771
dBET1
PROTAC
F
Control (G3)
DKO (E4)
UBE2R1
UBE2R2
DMSO
MZ1
ARV-771
dBET1
kDa
BRD2
BRD3
BRD4
UBE2R1
UBE2R2
GAPDH
1 2 3 4 5 6 7 8 9 10 11 12 13 14 15 16
G
Control
DKO A10
DKO B3
DKO E4
UBE2R1
UBE2R2
kDa
ZER1
KLHDC2
UBE2R1
UBE2R2
GAPDH
1 2 3 4 5 6 7 8 9 10
H
DMSO
ARV-771
dBET1
MZ1
siNT
GFP-ARIH1res
siARIH1
kDa
BRD4
GFP-ARIH1res
ARIH1
GAPDH
I
MDA-MB-468
Control
UBE2R1
UBE2R2
normalized BRD2 levels
DMSO
MZ1
ARV-771
dBET1
PROTAC
J
MDA-MB-468
Control
UBE2R1
UBE2R2
normalized BRD3 levels
DMSO
MZ1
ARV-771
dBET1
PROTAC
K
MRC-5
UBE2R1
UBE2R2
DMSO
MZ1
ARV-771
dBET1
kDa
BRD2
BRD3
BRD4
UBE2R1
UBE2R2
GAPDH
1 2 3 4 5 6 7 8 9 10 11 12
L
MRC-5
Control
UBE2R1
UBE2R2
normalized BRD2 levels
DMSO
MZ1
ARV-771
dBET1
PROTAC
M
MRC-5
Control
UBE2R1
UBE2R2
normalized BRD3 levels
DMSO
MZ1
ARV-771
dBET1
PROTAC
N
BRD4
K367R BRD4
K368R BRD4
kDa
UBE2R2~UB* K48R
BRD4-UB* K48R
UB* K48R

Figure S2. UBE2R1 and UBE2R2 are necessary for efficient BRD2-4 degradation with CRL2-dependent PROTACs (related to Figures 2, 3 and 4 and Excel File S1)
(A) Total proteome volcano plot for the comparison of control and *UBE2R1*/*UBE2R2* double knockout (DKO) HEK 293T cells. Proteins that were enriched or depleted by 2-fold or greater have been highlighted red.
(B) Same as (A) but comparing control and Δ*UBE2G1* knockout cells.
(C) Representative Western blots comparing BRD2-4 levels upon treatment with PROTACs in control or *UBE2R1*/*UBE2R2* DKO HEK 293T cells.
(D) Graph of BRD2 protein levels from the HEK 293T cells (Figure 2C). Datapoints reflect triplicate technical replicates performed using control (clone D5) and DKO (clone A10) cell lines.
(E) Same as in (D) except for BRD3 protein levels.
(F) Biological replicate of the experiment shown in panel (C).
(G) Representative Western blots comparing CRL2-dependent substrate receptor levels for ZER1 and KLHDC2 in the indicated cell lines. The effects of UBE2R1 or UBE2R2 expression on ZER1 and KLHDC2 levels are shown. The asterisk denotes cross-reactivity of the anti-KLHDC2 antibody with a nonspecific protein.
(H) Representative Western blots comparing the indicated protein levels upon treatment with PROTACs in control or siRNA-mediated ARIH1 knockdown Flp-In T-Rex HEK 293 cells. GFP-ARIH1^{res} indicates ARIH1 expression for protein that is not susceptible to the siRNA treatment.
(I) Graphical representation of BRD2 levels derived from MDA-MB-468 cells that had been treated with the indicated PROTACs or DMSO and upon ectopic expression of UBE2R1 or UBE2R2. * (p-value < 0.05) represents the statistical significance of the indicated comparisons as derived by an unpaired t test with Welch's correction.
(J) Same as in (I) except for BRD3. **** (p-value < 0.0001).
(K) Representative Western blots showing the indicated protein levels in the MRC-5 cell line that had been treated with the indicated PROTACs or DMSO and upon ectopic expression of UBE2R1 or UBE2R2.
(L) Same as in (I) except with MRC-5 cells.
(M) Same as in (J) except with MRC-5 cells. * (p-value < 0.05)
(N) Fluorescence scan of UBE2R2 "pulse-chase" ubiquitylation reactions containing fluorescently labeled K48R ubiquitin (UB* K48R), neddylated CRL2^{VHL}, and the indicated BRD4 (346-460) proteins in the presence of the PROTAC MZ1.

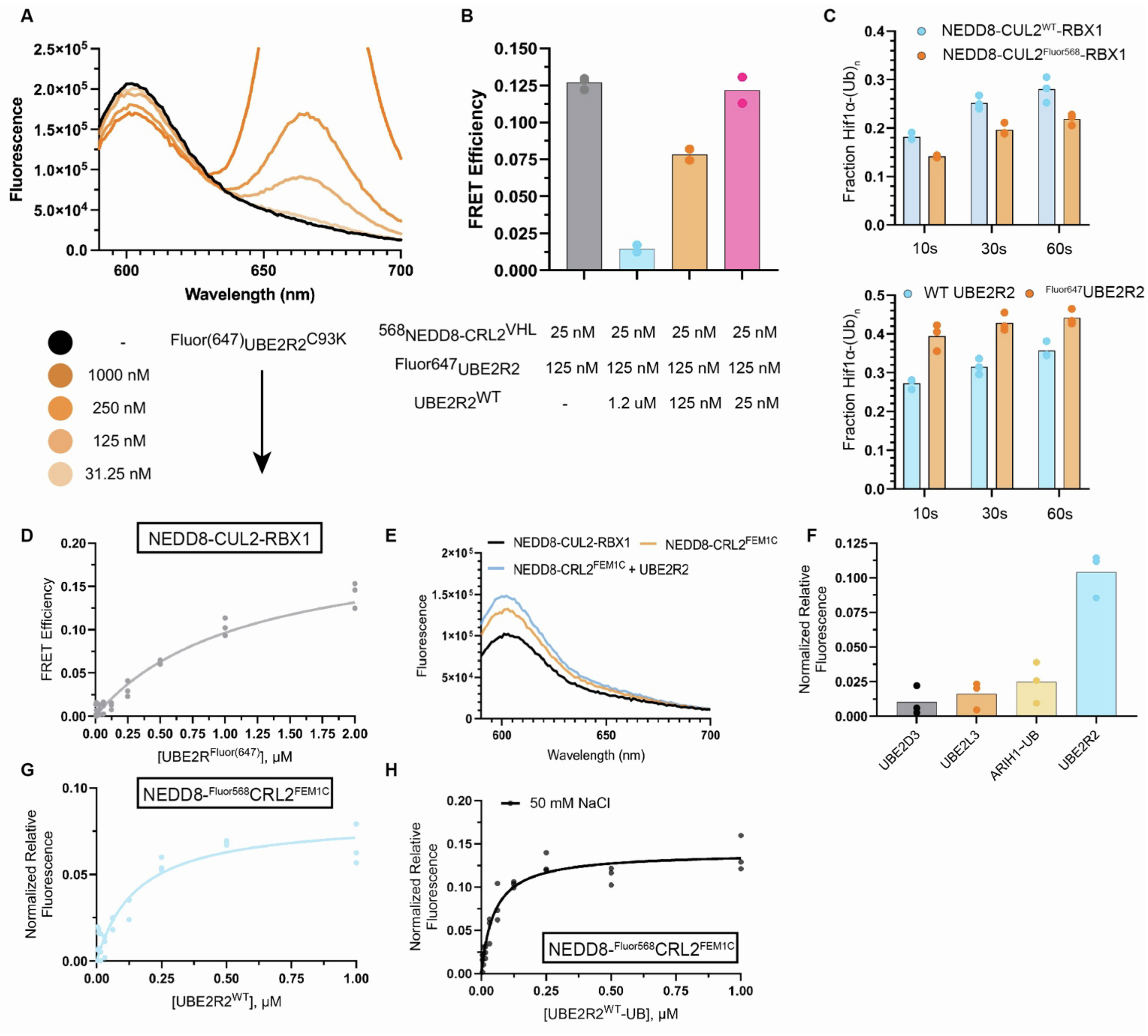

Figure S3. Development of fluorescence-based binding assays between UBE2R2 and CRL2s (related to Figure 4, Table S2, and STAR methods)

(A) Fluorescence emission scans of solutions containing either neddylated ^{Fluor568}CUL2-RBX1 (see STAR methods) alone or in combination with increasing levels of ^{Fluor647}UBE2R2^{C93K}, showing decreases in the donor fluorescence signal that correlated with increasing amounts of labeled UBE2R2.

(B) Control FRET experiments showing that increasing levels of unlabeled UBE2R2 competes with ^{Fluor647}UBE2R2^{C93K} binding to neddylated ^{Fluor568}CRL2^{VHL}, resulting in the decrease and eventual loss of FRET signal.

(C) Time courses for control ubiquitylation reactions comparing neddylated CUL2-RBX1 or neddylated ^{Fluor568}CUL2-RBX1 activities with ELONGIN B/C-VHL and ^{32}P-labeled Hif1α peptide substrate (top) or comparing wild-type (WT) UBE2R2 activity with fluorescently labeled UBE2R2^{WT} (^{Fluor647}UBE2R2; bottom).

(D) Graph of the FRET efficiency versus labeled UBE2R2 titration in the presence of fluorescently labeled, neddylated CUL2-RBX1 and reaction buffer at ionic strength of 100 mM. The data were fit to a one-site binding model by nonlinear regression (GraphPad Prism software v10).

(E) Fluorescence emission scan for neddylated ^{Fluor568}CUL2-RBX1 and the effect of adding ELONGIN B/C-FEM1C alone or in combination with unlabeled WT UBE2R2.

(F) Graph showing the normalized change in neddylated ^{Fluor568}CRL2^{FEM1C} fluorescence in the presence of various unlabeled UCEs.
(G) Graph showing the normalized change in neddylated ^{Fluor568}CRL2^{FEM1C} fluorescence upon the titration of unlabeled UBE2R2 in reaction buffer at ionic strength of 100 mM. The data were fit to a one-site binding model by nonlinear regression (GraphPad Prism software v10).
(H) Graph showing the normalized change in neddylated ^{Fluor568}CRL2^{FEM1C} fluorescence upon the titration of unlabeled, UBE2R2^{C93K}-ubiquitin protein (see STAR methods).
All experiments were performed with triplicate technical replicates, except for experiments in (F) containing WT UBE2R2, which were performed in duplicate. UB; ubiquitin.

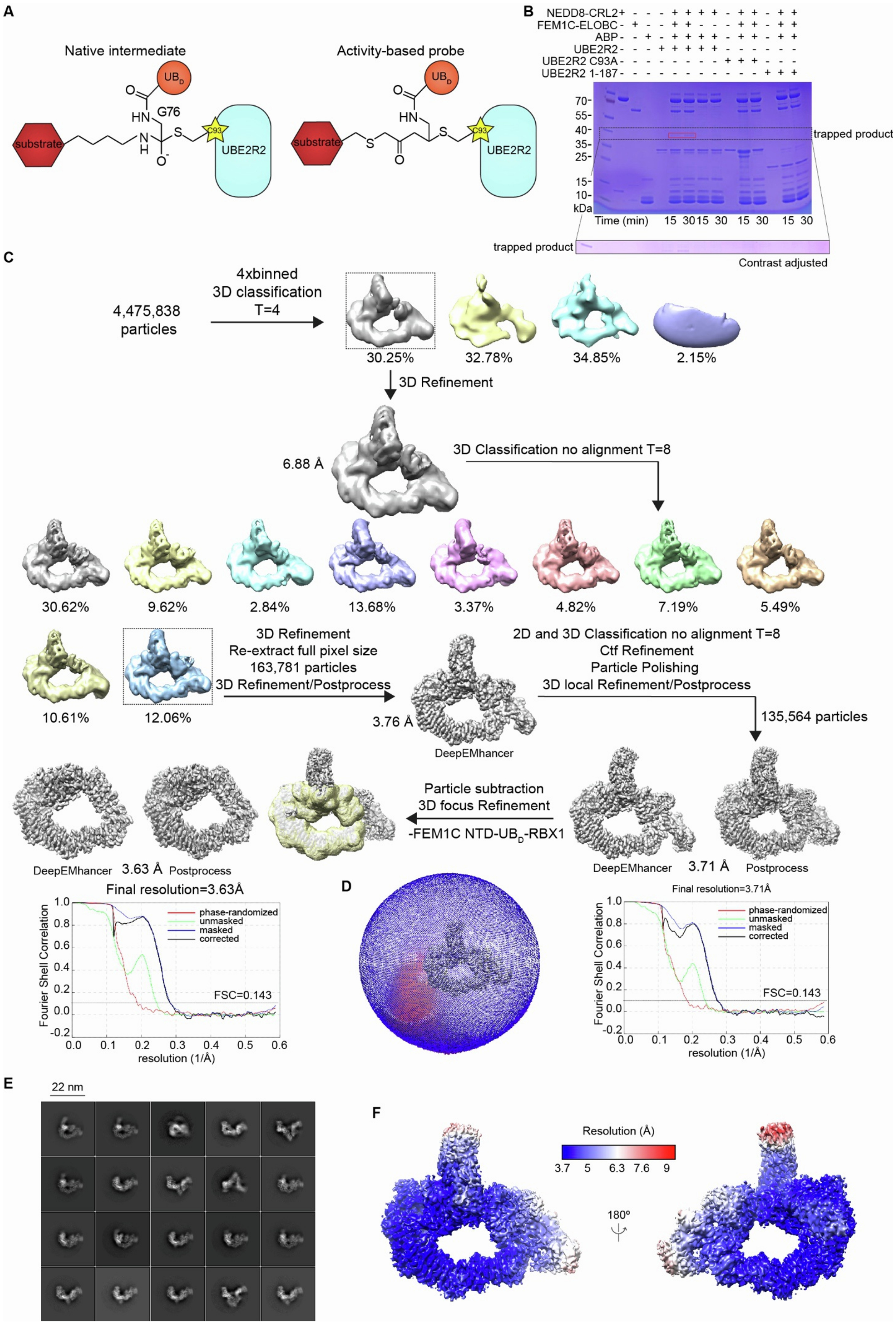
A
Native intermediate
Activity-based probe
substrate
UBE2R2
C93
G76
B
NEDD8-CRL2
FEM1C-ELOBC
ABP
UBE2R2
UBE2R2 C93A
UBE2R2 1-187
trapped product
Time (min)
kDa
Contrast adjusted
C
4,475,838 particles
4xbinned 3D classification T=4
30.25%
32.78%
34.85%
2.15%
3D Refinement
6.88 Å
3D Classification no alignment T=8
30.62%
9.62%
2.84%
13.68%
3.37%
4.82%
7.19%
5.49%
10.61%
12.06%
3D Refinement
Re-extract full pixel size
163,781 particles
3D Refinement/Postprocess
3.76 Å
DeepEMhancer
2D and 3D Classification no alignment T=8
Ctf Refinement
Particle Polishing
3D local Refinement/Postprocess
135,564 particles
Particle subtraction
3D focus Refinement
-FEM1C NTD-UB$_D$-RBX1
DeepEMhancer
3.63 Å
Postprocess
DeepEMhancer
3.71 Å
Postprocess
Final resolution=3.63Å
Final resolution=3.71Å
Fourier Shell Correlation
resolution (1/Å)
phase-randomized
unmasked
masked
corrected
FSC=0.143
D
E
22 nm
F
Resolution (Å)
3.7 5 6.3 7.6 9
180°

Figure S4. Design and implementation of the UBE2R2 activity-based probe and cryo-EM image processing flow chart (related to Figure 5, Table 1 and STAR methods)

(A) Diagram showing the transition state configuration of UBE2R2-catalyzed substrate priming, where the native geometry is shown on the left and the chemically trapped arrangement on the right.

(B) Coomassie-stained SDS-PAGE gel showing the purities of the indicated reagents before and after their reaction with the activity-based probe (see STAR methods). Notice that catalytically inactive UBE2R2 is incapable of forming the trapped complex, indicating that cross-linking occurs through the UBE2R2 active site Cys residue. Two contrast levels are shown due to the low level of trapped product (UBE2R2~ubiquitin-Sil1 peptide) relative to the reactants.

(C) Flow chart showing the stages of cryo-EM image processing, with reconstruction yielding a focused refinement at 3.63 Å resolution and a global refinement at 3.71 Å. The Fourier shell correlation (FSC) curves are shown for both refinements.

(D) Angular distribution of the reconstruction.

(E) 2-D classes representing particles used for the final reconstructions.

(F) Consensus EM density map colored by local resolution.

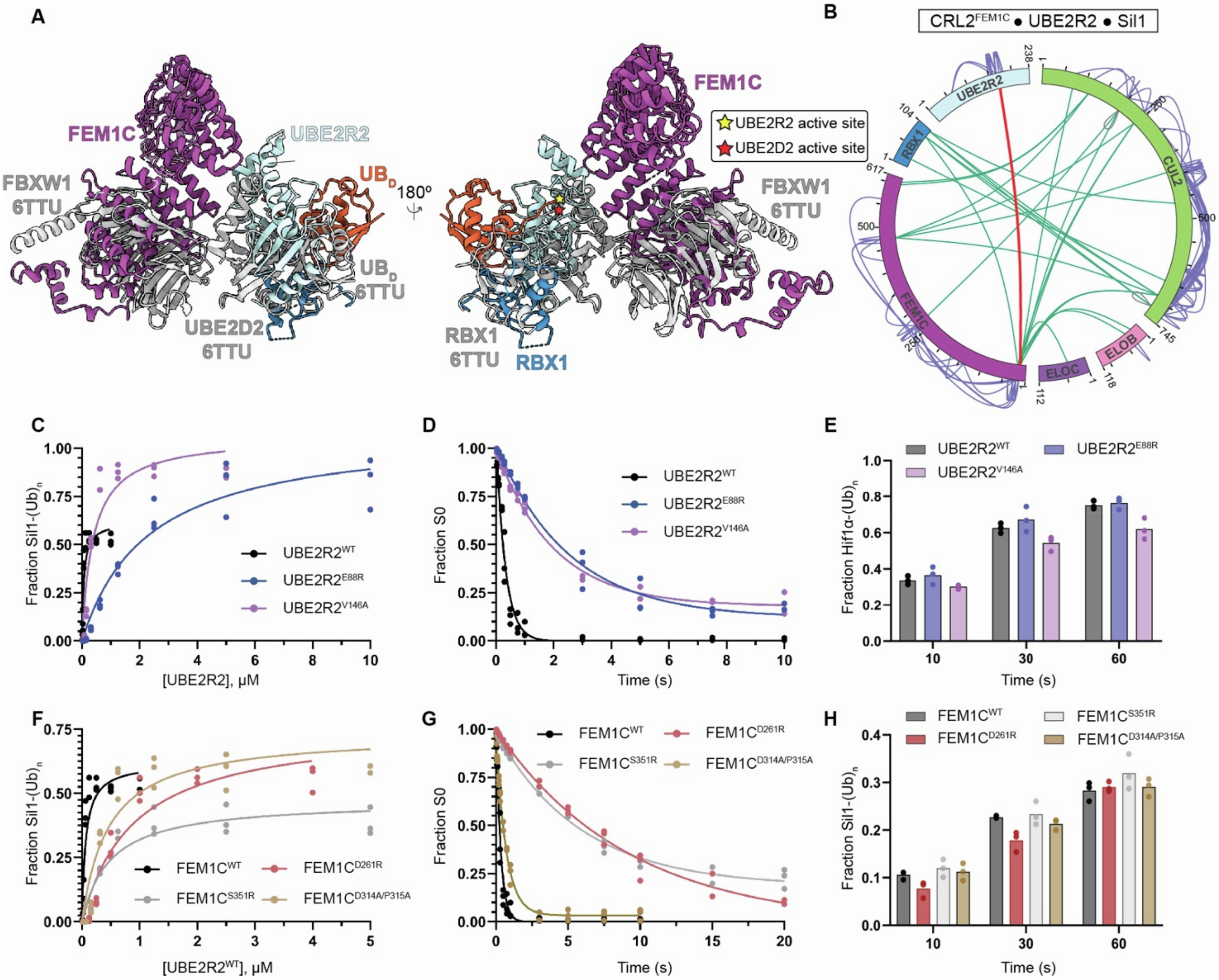

Figure S5. Biochemical validation of the UBE2R2-FEM1C interface (related to Figure 5, and Table S1)

(A) Structural comparison of neddylated CRL1^{FBXW1} with UBE2D2~ubiquitin and IKBα peptide substrate priming (gray; 6TTU) and the neddylated CRL2$^{FEM1C\text{-}Sil1}$-UBE2R2~ubiquitin priming structure. Coloring of the subunits in the priming structure are defined in Figure 5A. Donor ubiquitin (UB$_D$) is activated by UBE2R2 for priming of unmodified substrate.

(B) Diagram showing intra- (light purple) and inter-subunit (green) cross-linking of an un-neddylated CRL2^{FEM1C} complex in the presence of UBE2R2 and Sil1. Cross-links between FEM1C and UBE2R2 have been colored red to highlight their positions.

(C) Graph showing the fraction of Sil1 peptide substrate converted to ubiquitylated product in the presence of neddylated CRL2^{FEM1C} and titrations of wild-type (WT) UBE2R2 or the indicated mutants. The fit of the data to the Michaelis-Menten model are shown.

(D) Pre-steady state ubiquitylation reactions showing depletion of Sil1 peptide upon conversion to product as a function of time for various UBE2R2 mutants.

(E) Control ubiquitylation reactions with Hif1α peptide substrate, neddylated CRL2^{VHL}, and the indicated UBE2R2 proteins.

(F) Same as in (C) except with the indicated FEM1C mutants.

(G) Same as (D), except with the indicated FEM1C mutants.

(H) Control ARIH1 ubiquitylation reactions with neddylated CRL2^{FEM1C} containing WT or the indicated FEM1C mutants.

Cross-linking experiments were performed with duplicate technical replicates and ubiquitylation reactions with triplicate replicates.

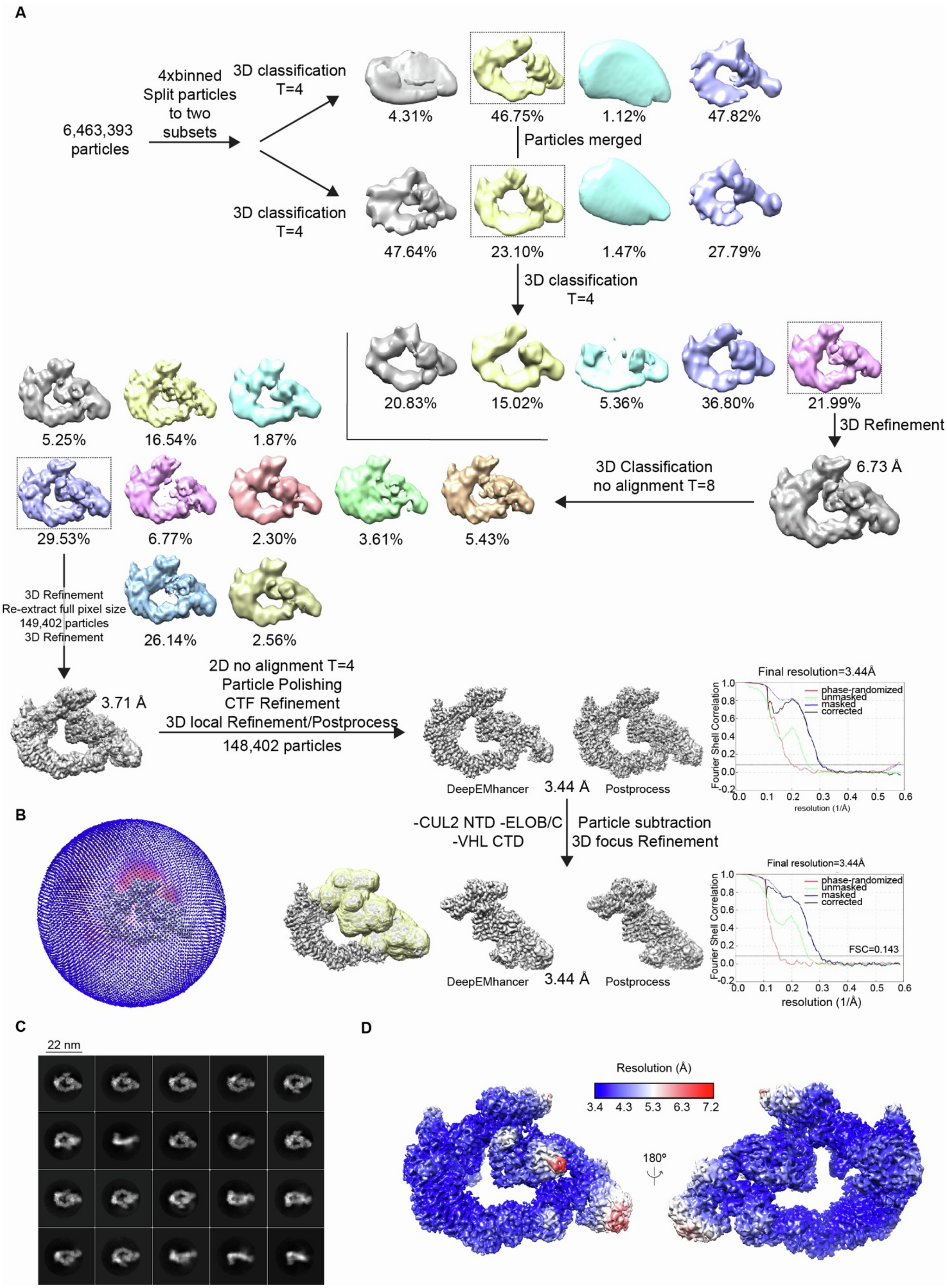

A
6,463,393 particles
4xbinned Split particles to two subsets
3D classification T=4
4.31%
46.75%
1.12%
47.82%
Particles merged
47.64%
23.10%
1.47%
27.79%
3D classification T=4
20.83%
15.02%
5.36%
36.80%
21.99%
3D Refinement
6.73 Å
3D Classification no alignment T=8
5.25%
16.54%
1.87%
29.53%
6.77%
2.30%
3.61%
5.43%
26.14%
2.56%
3D Refinement
Re-extract full pixel size
149,402 particles
3D Refinement
3.71 Å
2D no alignment T=4
Particle Polishing
CTF Refinement
3D local Refinement/Postprocess
148,402 particles
DeepEMhancer
3.44 Å
Postprocess
Final resolution=3.44Å
phase-randomized
unmasked
masked
corrected
Fourier Shell Correlation
resolution (1/Å)
-CUL2 NTD -ELOB/C -VHL CTD
Particle subtraction 3D focus Refinement
B
FSC=0.143
C
22 nm
D
Resolution (Å)
3.4 4.3 5.3 6.3 7.2
180°

Figure S6. Cryo-EM image processing flow chart for the neddylated CRL2$^{VHL\text{-}MZ1\ BRD4\ (346\text{-}460)}$-UBE2R2~ubiquitin priming structure (related to Figure 6 and Table 1)

(A) Flow chart showing the stages of cryo-EM image processing, with reconstruction yielding a global refinement at 3.44 Å. The Fourier shell correlation (FSC) curves are shown for both refinements.

(B) Angular distribution of the reconstruction.

(C) 2-D classes representing particles used for the final reconstructions.

(D) Consensus EM density map colored by local resolution.

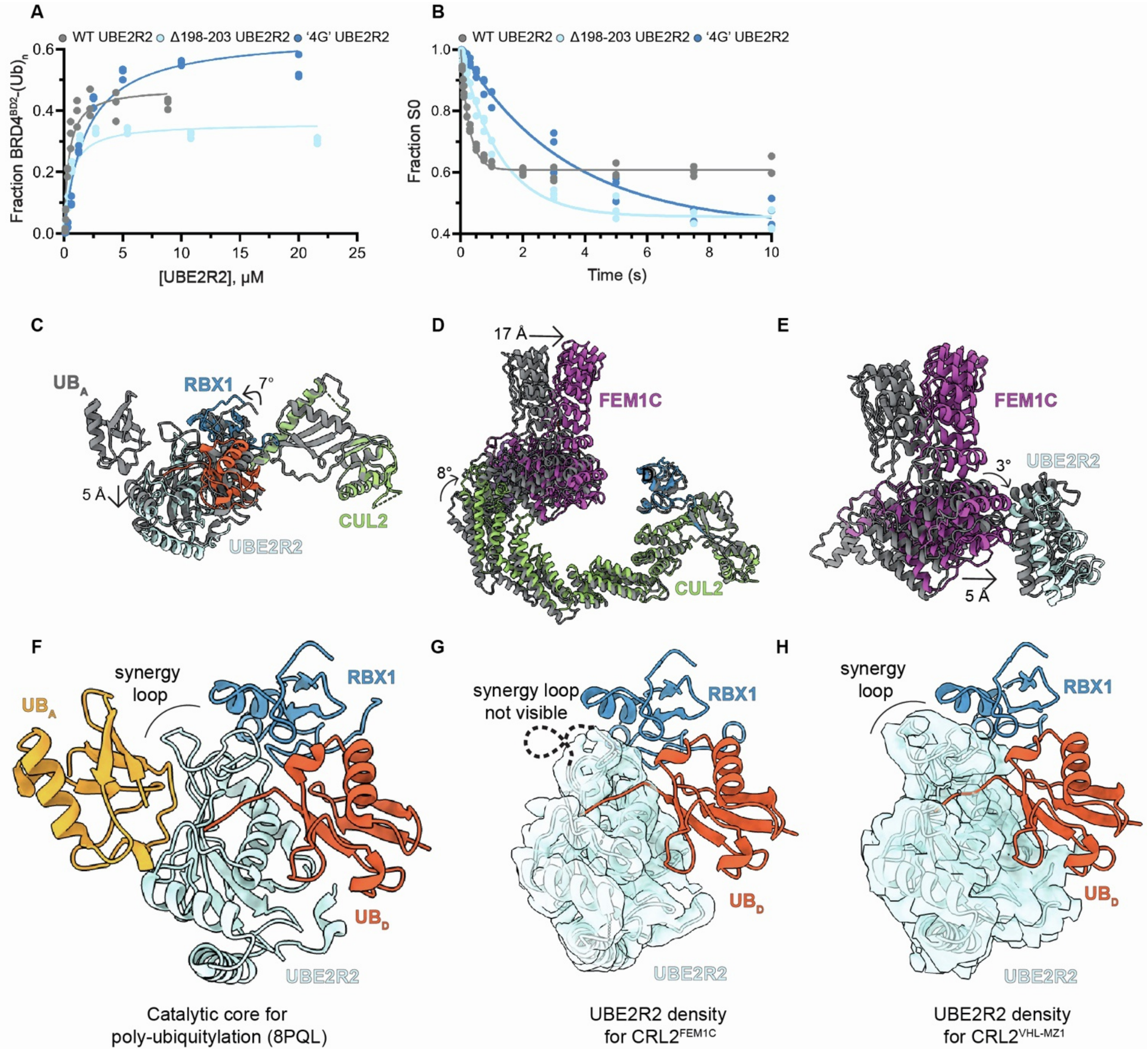

Figure S7. UBE2R2-mediated substrate priming and poly-ubiquitin chain extension structures illuminate distinct conformational states (related to Figures 6 and 7, and Table S1)

(A) Graph showing the results for the estimation of the K_m of UBE2R2 (wild-type (WT) and the indicated C-terminal acidic tail mutants) and the fit of the data to the Michaelis-Menten model. Δ198-203 UBE2R2 represents an internal deletion of residues 198 to 203 within the tail, while '4G' UBE2R2 is a L206G Y207G L210G Y211G quadruple point mutant.

(B) Graph showing the results from pre-steady state ubiquitylation reactions estimating the rate of BRD4 (346-460) priming, k_{obs}. The UBE2R2 mutants have been defined in (A).

(C) Superposition of the neddylated CRL2$^{FEM1C\text{-}Sil1}$-UBE2R2~ubiquitin priming structure and the corresponding cryo-EM poly-ubiquitin chain extension structure (gray; 8PQL). Coloring of the subunits in the priming structure are defined in Figure 5A. The alignment shows significant rotation and shifting of UBE2R2~ubiquitin relative to CUL2's C-terminal region.

(D) Same as (C) except focused on conformational changes to FEM1C relative to the middle cullin repeat on CUL2.

(E) Same as (C) except showing changes near the UBE2R2-FEM1C interface.

(F) Ribbon diagram of the UBE2R2~ubiquitin-RING catalytic core during poly-ubiquitin chain extension (8PQL), highlighting the interaction between UBE2R2's synergy loop and RBX1 as well as donor and acceptor ubiquitins (UB_D and UB_A, respectively).
(G) Same as (F) except for Sil1 peptide priming with neddylated $CRL2^{FEM1C}$. The electron density from the cryo-EM map for UBE2R2 is shown (notice a lack of density for the synergy loop region).
(H) Same as (G) except for BRD4 (346-460) priming with neddylated $CRL2^{VHL}$ in the presence of the PROTAC MZ1. Here electron density is visible for UBE2R2's synergy loop.

Supplemental References List

S1. Purser, N., Tripathi-Giesgen, I., Li, J., Scott, D.C., Horn-Ghetko, D., Baek, K., Schulman, B.A., Alpi, A.F., and Kleiger, G. (2023). Catalysis of non-canonical protein ubiquitylation by the ARIH1 ubiquitin ligase. Biochem J *480*, 1817-1831. 10.1042/BCJ20230373.

S2. Pierce, N.W., Kleiger, G., Shan, S.O., and Deshaies, R.J. (2009). Detection of sequential polyubiquitylation on a millisecond timescale. Nature *462*, 615-619. nature08595 [pii]
